# MYB Proto-Oncogene Like 2 identified as a biomarker for uterine corpus endometrial carcinoma: evidence from bioinformatics and clinical validation

**DOI:** 10.3389/fonc.2025.1595485

**Published:** 2025-05-13

**Authors:** Jiaoyun Lu, Furong Luo

**Affiliations:** ^1^ Department of Oncology, Xi’an NO.3 Hospital, The Affiliated Hospital of Northwest University, Xi’an, Shaanxi, China; ^2^ Department of Traditional Chinese Medicine, Xi’an NO.3 Hospital, The Affiliated Hospital of Northwest University, Xi’an, Shaanxi, China

**Keywords:** MYBL2, uterine corpus endometrial carcinoma, prognosis, immune infiltration, biomarker

## Abstract

**Background:**

Endometrial carcinoma (EC) is the sixth most prevalent malignancy among women globally, posing a significant clinical challenge due to limited therapeutic options for advanced or recurrent cases. The identification of novel prognostic biomarkers and therapeutic targets is crucial for improving patient outcomes. This study aimed to investigate the multifaceted roles of MYB Proto-Oncogene Like 2 (MYBL2) in uterine corpus endometrial carcinoma (UCEC).

**Methods:**

We employed multiple bioinformatics algorithms (GEPIA, TCGA, TIMER2.0) to analyze MYBL2 expression across different cancer types and in UCEC specifically. Expression patterns were validated using quantitative real-time PCR (qPCR) on clinical samples. Epigenetic analyses focused on promoter methylation status, and immune infiltration patterns were assessed using MethSurv, CIBERSORT and TIMER2.0. Drug sensitivity profiling was performed using the CPADS web platform.

**Results:**

MYBL2 was found to be significantly upregulated in UCEC tumors compared to normal tissues. Elevated MYBL2 expression correlated with advanced histologic grade and clinical stage, indicating its potential as a biomarker for disease progression. Epigenetic analysis revealed promoter hypomethylation in tumors, suggesting a regulatory mechanism driving MYBL2 overexpression. MYBL2 demonstrated dynamic interactions with the tumor immune microenvironment, including associations with immune cell infiltration patterns and co-expression with immune checkpoint molecules and chemokines. Drug sensitivity profiling highlighted differential therapeutic responses linked to MYBL2 expression levels.

**Conclusion:**

This study establishes MYBL2 as a critical regulator of UCEC progression, bridging epigenetic dysregulation, immune modulation, and clinical outcomes. The findings provide a foundation for exploring MYBL2-targeted strategies in precision immunotherapy and personalized therapeutic interventions.

## 1 Introduction

Endometrial carcinoma (EC) ranks as the sixth most prevalent malignancy among women globally, with approximately 420,000 new cases reported in 2020, posing a significant threat to female health (1). This disease is characterized by heterogeneous genetic alterations and clinical outcomes, with predominant histologic subtypes including endometrioid adenocarcinoma, serous carcinoma, and clear cell carcinoma (2). While conventional therapies such as surgery, chemotherapy, and radiotherapy have improved survival rates for early-stage EC patients (3), treatment options remain limited for advanced or recurrent cases, which are often associated with poor prognoses. Although immunotherapy has demonstrated clinical potential in various cancers by modulating anti-tumor immune responses, its application in EC remains restricted (4), underscoring the need to elucidate the molecular mechanisms underlying tumor progression and immune evasion.

MYB proto-oncogene like 2 (MYBL2), a critical transcriptional regulator of cell cycle progression, drives G2/M phase transition through E2F target gene activation (5). Aberrant MYBL2 overexpression has been implicated in tumor proliferation, invasion, and poor prognosis across multiple malignancies, including breast cancer, colorectal cancer, and glioma (6–8). Mechanistically, MYBL2 interacts with key oncogenic pathways such as Wnt/β-catenin (9) and PI3K/AKT signaling (10). Emerging evidence from single-cell sequencing and organoid models further highlights its role in shaping tumor heterogeneity and remodeling the tumor microenvironment (11, 12). While preclinical studies have demonstrated the anti-tumor potential of MYBL2-targeted therapies, including small-molecule inhibitors and CRISPR-based gene editing (13), challenges persist regarding specificity and off-target effects. Further investigation into MYBL2’s epigenetic regulatory networks and immunomodulatory functions is essential for developing precision therapeutic strategies.

Recent advances in EC research have focused on the tumor immune microenvironment and immunotherapy. EC frequently exhibits high tumor mutational burden (TMB) and microsatellite instability (MSI-H/dMMR), particularly in POLE-mutated and MSI-H subtypes (14, 15). These immunogenic “hot tumors” are characterized by increased tumor-infiltrating lymphocytes (TILs), PD-L1 expression, and upregulated immune checkpoint molecules (e.g., PD-1, LAG-3) (15). Clinically, PD-1/PD-L1 inhibitors (e.g., pembrolizumab, dostarlimab) have achieved regulatory approval for MSI-H/dMMR advanced or recurrent EC (16, 17), while combination therapies such as lenvatinib plus PD-1 inhibitors have extended clinical benefits to non-MSI-H populations (18). Novel immunotherapies including CTLA-4 bispecific antibodies, personalized neoantigen vaccines, and CAR-T cell therapies are under early clinical investigation (19). However, challenges such as therapeutic resistance and immune-related adverse events persist, necessitating multi-omics approaches to decipher immunosuppressive mechanisms (e.g., Treg infiltration, M2 macrophage polarization) and develop next-generation checkpoint inhibitors (20).

This study employed integrated bioinformatics analysis combined with clinical sample validation to assess MYBL2 as a prognostic biomarker for uterine corpus endometrial carcinoma (UCEC) and to elucidate its associations with methylation modification, immune infiltration patterns, and sensitivity to antitumor drugs. Additionally, we investigated MYBL2-related functional pathways to identify MYBL2 as a potential target for novel immunotherapy strategies in UCEC.

## 2 Method

### 2.1 Data sources and differential analysis of MYBL2 expression

Transcriptome data for 33 cancer types from TCGA (https://tcga.xenahubs.net) (21) were obtained, and the differential expression of MYBL2 was examined via the Wilcoxon test functionality within the R package. The full names and the sample sizes of the tumor abbreviations used in this study can be found in **Supplementary Table 1**. To stratify the dataset, we categorized samples into high- versus low-expression cohorts based on the median MYBL2 expression value. Statistical significance was defined as a false discovery rate (FDR) <0.05. For tissue-level validation of MYBL2 expression patterns, immunohistochemical staining data from diverse tumor specimens were analyzed via the Human Protein Atlas (HPA, https://www.proteinatlas.org) database. This revealed marked MYBL2 expression across multiple cancer types, corroborating its broad oncogenic relevance. (22).

### 2.2 Verification of expression differences in clinical samples using quantitative real-time PCR

Tissue samples, comprising both tumor and adjacent non-cancerous tissues, were collected from 10 patients diagnosed with UCEC at Xi’an No. 3 Hospital. The study protocol received approval from the Ethics Committee of Xi’an No. 3 Hospital (Approval No. SYLL-2025-025), and informed consent for sample collection was obtained from all patients, with detailed diagnostic information presented in **Supplementary Table 2**.

The primers utilized in the experiments are detailed in **Supplementary Table 3**. Total RNA was extracted from these samples, followed by cDNA synthesis. Quantitative Real-Time PCR (qPCR) was then applied to evaluate MYBL2 expression levels, using actin as the internal reference gene for normalization, thus ensuring reliable validation of MYBL2 expression patterns in UCEC. To investigate the differential expression of MYBL2 between cancer and normal tissues in UCEC, a two-sample t-test was conducted on the qPCR data. This statistical method compares the mean expression levels of the two groups to ascertain significant differences. The t-test was performed using R language, yielding a t-statistic, p-value, confidence interval for the difference in means, and sample estimates of the mean MYBL2 expression levels for both groups.

### 2.3 MBYL2 methylation status and prognosis in UCEC

The methylation levels of the MYBL2 gene in normal and primary tumor tissues were obtained from TCGA database (23). The log2 transformation (log2(value+0.001)) was applied to calculate the Pearson correlation between MYBL2 and mRNA methylation modification genes.

To analyze the methylation of the MYBL2 promoter in UCEC, MethSurv (24) was utilized. In MethSurv, the “Gene Visualization” option was selected, along with the dataset “Uterine Endometrial Cancer [UCEC] TCGA December 2024.” Additionally, by selecting the “Single CpG” option and inputting “MYBL2” within the same UCEC dataset, a survival analysis was conducted on the available methylation sites in the database.

### 2.4 MYBL2 expression is associated with the prognosis of patients with UCEC

The clinicopathological and survival data for UCEC samples were obtained from the TCGA database. To evaluate the prognostic accuracy of MYBL2, ROC analysis was performed on the data using the pROC package, with the results visualized using ggplot2. Differential analysis of clinical factors (histologic grade, clinical stage, and histological type) was performed using the “limma” R package to examine the association between MYBL2 and clinical variables. Additionally, To assess the prognostic value of MYBL2 in UCEC, all samples were classified into high and low MYBL2 expression groups based on the median expression values. Survival analysis, including overall survival (OS), disease-specific survival (DSS), progression-free interval (PFI), and disease-free interval (DFI), was conducted using the R survival package, and the results were presented as Kaplan-Meier curves with log-rank p-values.

### 2.5 Immune infiltration in UCEC

Using the CIBERSORT algorithm, the distribution of MYBL2 across 22 distinct immune cell types can be quantified (25). Additionally, the R “ESTIMATE” package was used to calculate stromal and immune scores, with their sum representing the ESTIMATE score, which indirectly reflects tumor purity (26). The CIBERSORT algorithm was used to estimate the immune cell infiltration abundance in UCEC tissue. Specifically, the Pearson correlation coefficient (Pearson R) was employed to assess the correlation between MYBL2 expression and the inferred abundance of different immune cell types. Additionally, the “Gene Module” in TIMER2.0 was utilized to further evaluate the relationship between MYBL2 and immune cell infiltration (27). This module generates scatter plots to display the Spearman correlation between MYBL2 expression and tumor purity, as well as the abundance of six immune cell types, including dendritic cells (DC), B cells, neutrophils, CD4+ T cells, macrophages, and CD8+ T cells. Additionally, the R packages (“ggplot2”, “ggpubr”, and “ggExtra”) were used for further analysis of the correlations between MYBL2 expression, stromal and immune scores, and immune cell infiltration abundance.

### 2.6 Co-expression analysis of MYBL2 and immune genes in UCEC

Chemokines, their receptors, and immune genes are key determinants of the tumor immune microenvironment. Co-expression analysis between MYBL2 expression and the expression of immunosuppressive and immune activation genes, as well as MHC,chemokine, and chemokine receptor-related genes, was performed using the R “limma” package. Expression data of immune checkpoint genes were extracted, and their expression in two groups of UCEC samples stratified by high and low MYBL2 expression was analyzed using the R “ggplot2” package.

### 2.7 Pathway enrichment analysis

Gene expression profiles related to MYBL2 were analyzed to identify differentially expressed genes based on MYBL2 expression levels. The median expression value of MYBL2 was used to divide samples into high-expression and low-expression groups. Spearman correlation analysis was performed to determine gene correlations with MYBL2 expression. The results were visualized through the generation of volcano plots and heatmaps, providing a clear view of the distribution and expression patterns of related genes. R software (version 4.2.1) was used for data analysis and visualization, specifically employing the ggplot2 package (version 3.4.4) to construct the volcano plots and heatmaps.

Following identification of the correlated genes, functional enrichment analysis was conducted to explore the biological significance of MYBL2-associated genes. Gene Ontology (GO) and Kyoto Encyclopedia of Genes and Genomes (KEGG) pathway enrichment analyses were performed using the DAVID (https://david.ncifcrf.gov/) and Metascape (https://metascape.org/) online platforms. These analyses provided insights into the key biological processes, cellular components, molecular functions, and pathways associated with MYBL2 expression.

To further investigate protein-level interactions, a protein-protein interaction (PPI) network was constructed. The STRING database (https://string-db.org/) was utilized to retrieve interaction data for MYBL2 and its related genes. The PPI network was visualized to highlight key gene interactions and interaction strength.

Subsequently, pathway clustering analysis was performed to classify the enriched pathways based on their similarities. The Jaccard similarity index (IC) was applied to calculate the pairwise similarity of enrichment terms. Hierarchical clustering was then conducted using the hclust function in R. The clustering results were visualized using the ggplot2 package (version 3.4.4) to present the relationship and grouping of the enriched pathways clearly.

### 2.8 Drug sensitivity analysis

The Comprehensive Pancancer Analysis of Drug Sensitivity (CPADS) web platform (accessible at https://smuonco.shinyapps.io/CADSP/) was utilized to investigate the drug sensitivity profiles associated with the MYBL2 gene in uterine corpus endometrial carcinoma (UCEC). CPADS integrates data from multiple sources, including the Gene Expression Omnibus (GEO), TCGA, and the Genomics of Drug Sensitivity in Cancer (GDSC) databases, enabling a comprehensive analysis of differential gene expression and drug response. Focus was placed on the MYBL2 gene to assess its impact on drug sensitivity in UCEC. By utilizing the TCGA modules within CPADS, the IC50 values (concentration required for half-maximal inhibition) of various anticancer drugs were evaluated in UCEC samples with high versus low MYBL2 expression. IC50 values, which represent the drug concentration needed to inhibit 50% of cellular growth, are commonly used as a metric to gauge drug sensitivity.

## 3 Result

### 3.1 Expression of MYBL2 in pan-cancer and UCEC

**Figure 1:**
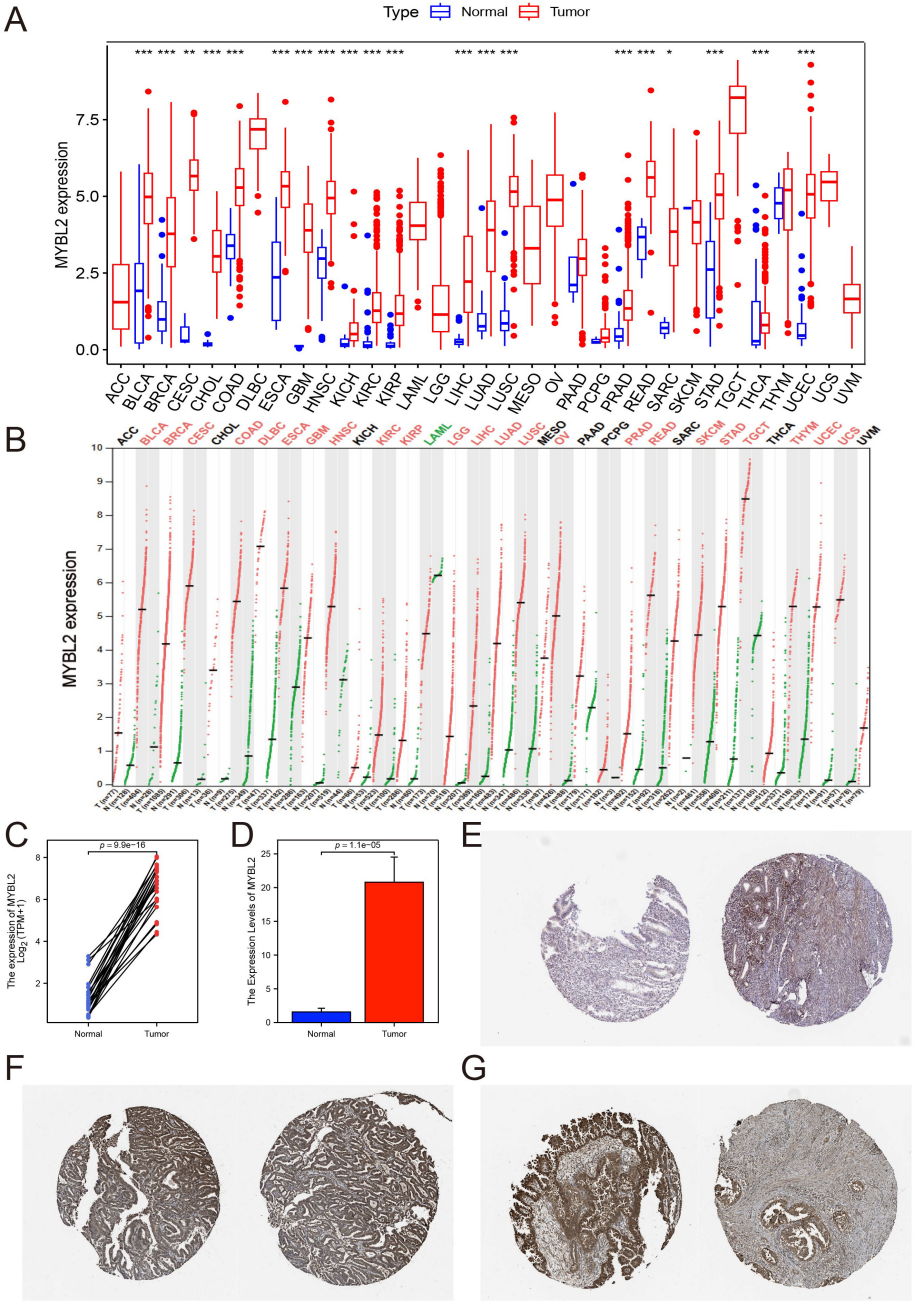
The expression of MYBL2 across pan-cancer types, with a particular focus on UCEC. **(A)** Analysis of MYBL2 expression levels in various cancer types or tumor tissues and adjacent non-tumor tissues using the R package from the TCGA databases (*p<0.05, **p<0.01, ***p<0.001); **(B)** Analysis of MYBL2 expression levels in various cancer types or tumor tissues and adjacent non-tumor tissues from the GEPIA databases; **(C)** Differential MYBL2 expression between UCEC tumor samples and normal tissue samples from TCGA and GTEx; **(D)** qPCR results; **(E)** Representative images and quantification of MYBL2 immunohistochemical staining in normal tissues; **(F, G)**. Representative images and quantification of MYBL2 immunohistochemical staining in UCEC tissues.

To evaluate the potential of MYBL2 as a tumor biomarker, its expression patterns across various cancer types were first examined by comparing tumor tissues with their corresponding normal tissues. Analysis conducted using the R package revealed elevated expression levels of MYBL2 in bladder urothelial carcinoma (BLCA), breast invasive carcinoma (BRCA), cholangiocarcinoma (CHOL), colon adenocarcinoma (COAD), esophageal carcinoma (ESCA), head and neck squamous cell carcinoma (HNSC), kidney chromophobe (KICH), kidney renal clear cell carcinoma (KIRC), kidney renal papillary cell carcinoma (KIRP), liver hepatocellular carcinoma (LIHC), lung adenocarcinoma (LUAD), lung squamous cell carcinoma (LUSC), prostate adenocarcinoma (PRAD), rectum adenocarcinoma (READ), stomach adenocarcinoma (STAD), and uterine corpus endometrial carcinoma (UCEC) compared to matched normal controls (**Figure 1A**). Furthermore, consistent results were obtained through analysis utilizing the GEPIA database (**Figure 1B**).

Paired sample analysis of TCGA data further confirmed significantly higher expression of MYBL2 in the tumor group compared to the control group (*p* = 9.9e-16, **Figure 1C**). Consistent results were obtained from qPCR experiments conducted on collected clinical samples (*p* = 1.1e-05, **Figure 1D**, **Supplementary Table 4**). To further validate the robustness of these findings, pathological slides stained for MYBL2 in healthy endometrium and UCEC patients were downloaded from the HPA database, revealing markedly darker staining in UCEC samples compared to healthy samples (**Figures 1E–G**).

### 3.2 Analysis of MBYL2 expression and mRNA levels in relation to promoter methylation status in endometrial carcinoma

**Figure 2:**
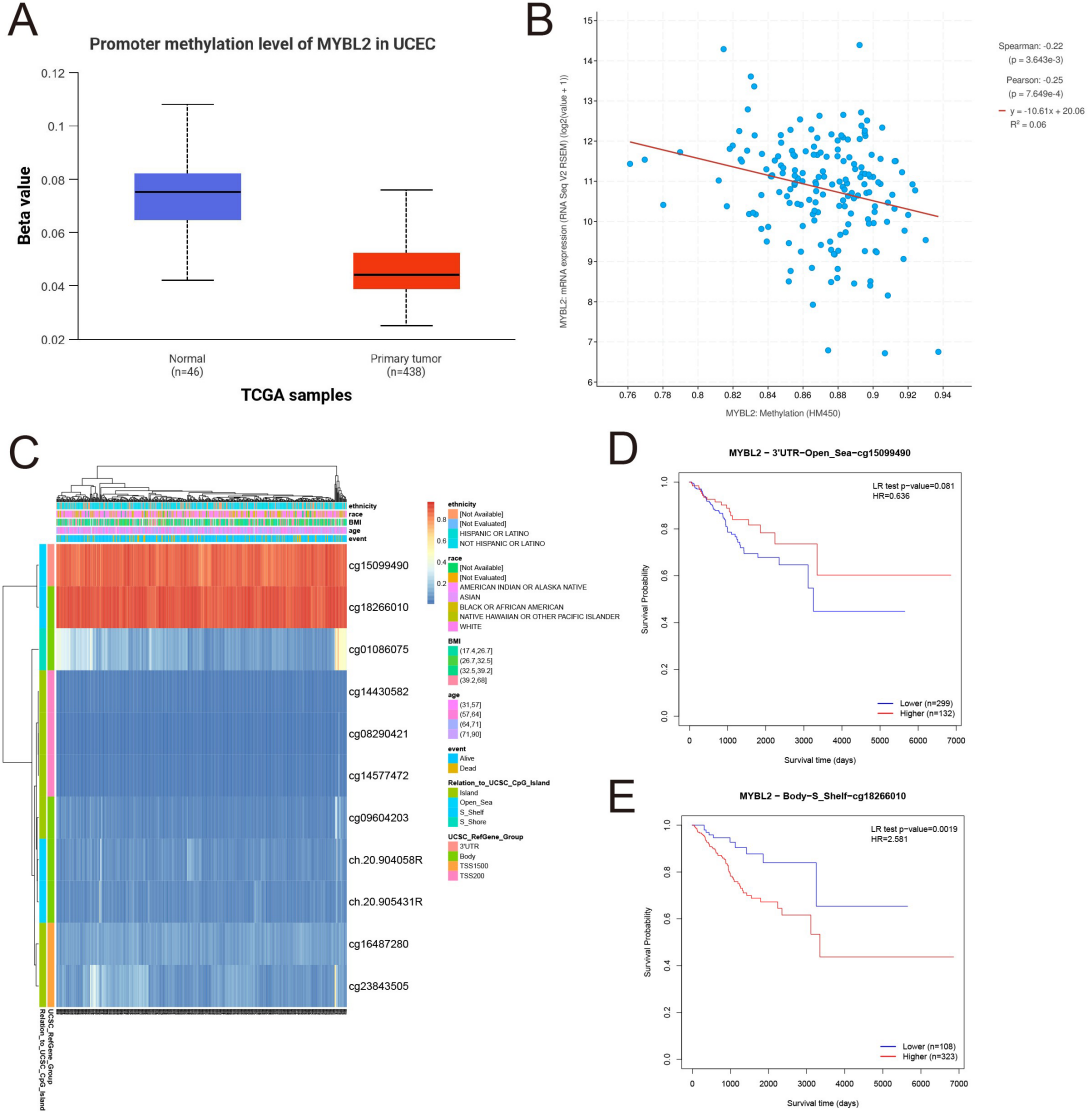
Analysis of MYBL2 methylation expression. **(A)** Expression analysis of MYBL2 in UCEC; **(B)** The relationship between MYBL2 promoter methylation and mRNA expression; **(C)** Methylation profile of MYBL2 in UCEC; **(D)** Survival analysis based on the methylation status of the cg15099490 site; **(E)** Survival analysis based on the methylation status of the cg18266010 site.

To investigate the methylation patterns of MYBL2, a methylation analysis was conducted comparing normal tissues and tumor tissues (**Figure 2A**). The results indicated that the methylation levels in normal tissues were significantly higher than those in primary tumor tissues. Subsequently, the mRNA expression levels of the MYBL2 gene were log2 transformed (log2(value + 1)) for Spearman and Pearson correlation analyses. The findings revealed a negative correlation between MYBL2 methylation levels and mRNA expression levels (**Figure 2B**), with Spearman (*p* = 3.643e-3) and Pearson (*p* = 7.649e-4) coefficients. Further analysis of MYBL2 methylation profiles in uterine endometrial cancer (UCEC) identified cg15099490 and cg18266010 as loci with elevated methylation levels (**Figure 2C**). Survival analysis focused on the methylation status of these loci, yielding corresponding survival curves. Notably, increased methylation level at cg18266010 was associated with poor prognosis (**Figures 2D, E**; cg15099490: *p* = 0.081, HR = 0.636; cg18266010: *p* = 0.0019, HR = 2.581).

### 3.3 MYBL2 expression is associated with prognosis of UCEC patients

**Figure 3:**
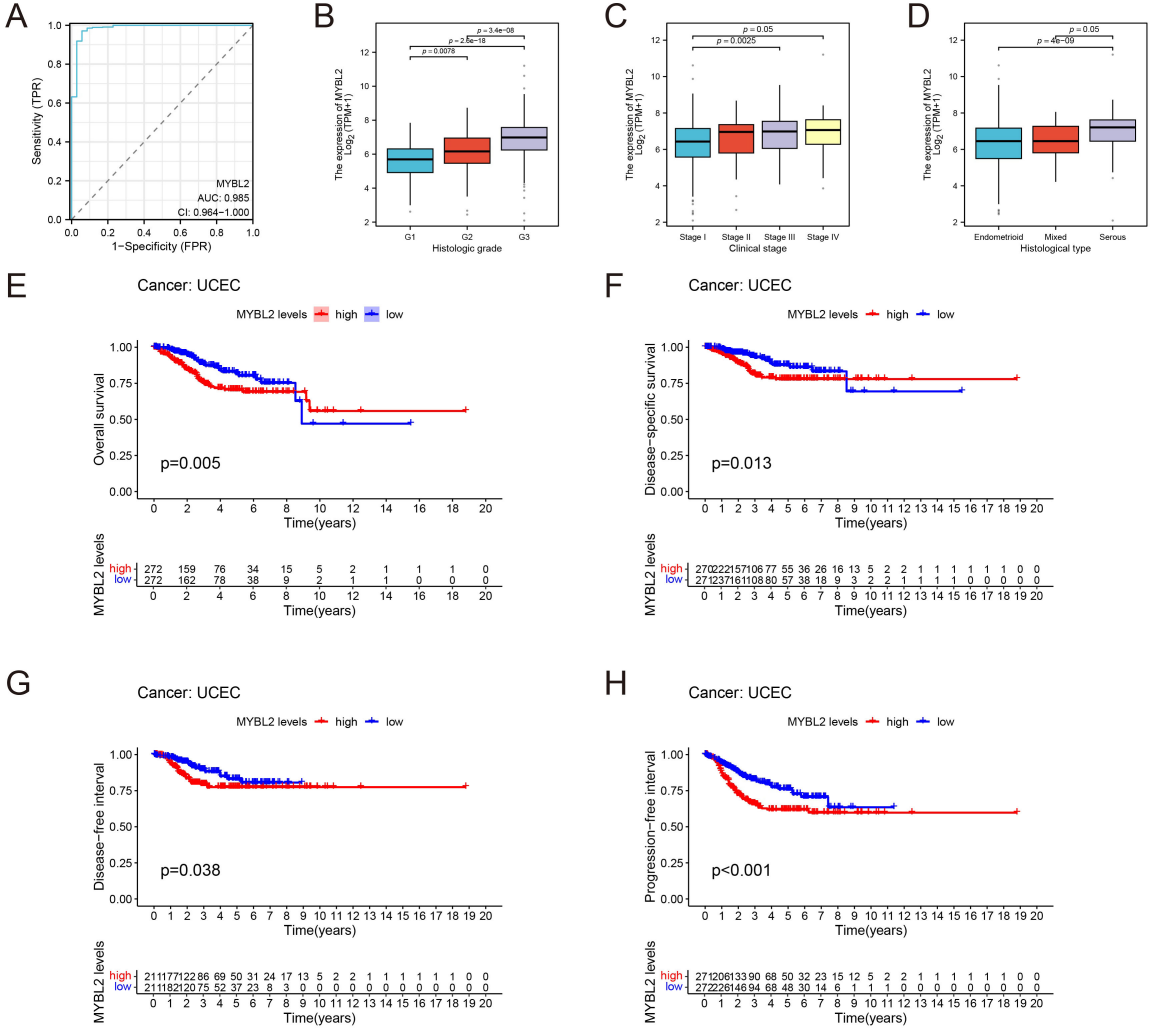
Prognostic value of MYBL2 in UCEC. **(A)** ROC curve of MYBL2 as a biomarker; **(B–D)** Correlation between MYBL2 expression differences and clinical factors, represented in a box plot; **(E)** Overall survival, **(F)** Disease-specific survival; **(G)** Progression-free interval; **(H)** Disease-free interval.

The prognostic significance of MYBL2 in UCEC was assessed through survival analysis. ROC curve analysis revealed that MYBL2 exhibited a prognostic area under the curve (AUC) of 0.985 (CI: 0.964–1.000, **Figure 3A**).

Subsequent analysis investigated the relationship between MYBL2 expression and clinical factors, including histological grade, clinical stage, and histological type (**Figures 3B–D**). Notably, MYBL2 expression was significantly higher in G3 patients compared to G1 patients (*p* = 3.4e−08). Significant differences in MYBL2 expression were also observed among the Endometrioid, Mixed, and Serous histological types (*p* = 4e−09 and *p* = 0.05, respectively). In terms of clinical stages, MYBL2 expression varied significantly, with notable differences between Stage I, Stage II, Stage III, and Stage IV (*p* = 0.05), as well as between Stage I, Stage II, and Stage III (*p* = 0.0025).

Four key clinical outcomes were evaluated, revealing that patients with high MYBL2 expression had significantly worse overall survival (OS, *p* = 0.005), disease-specific survival (DSS, *p* = 0.01), disease-free interval (DFI, *p* = 0.038), and progression-free interval (PFI, *p* = 0.001) compared to those with low MYBL2 expression. These results highlight the association between MYBL2 expression and survival outcomes in UCEC patients (**Figures 3E–H**).

### 3.4 Association of MYBL2 expression with immune infiltrates in UCEC

**Figure 4:**
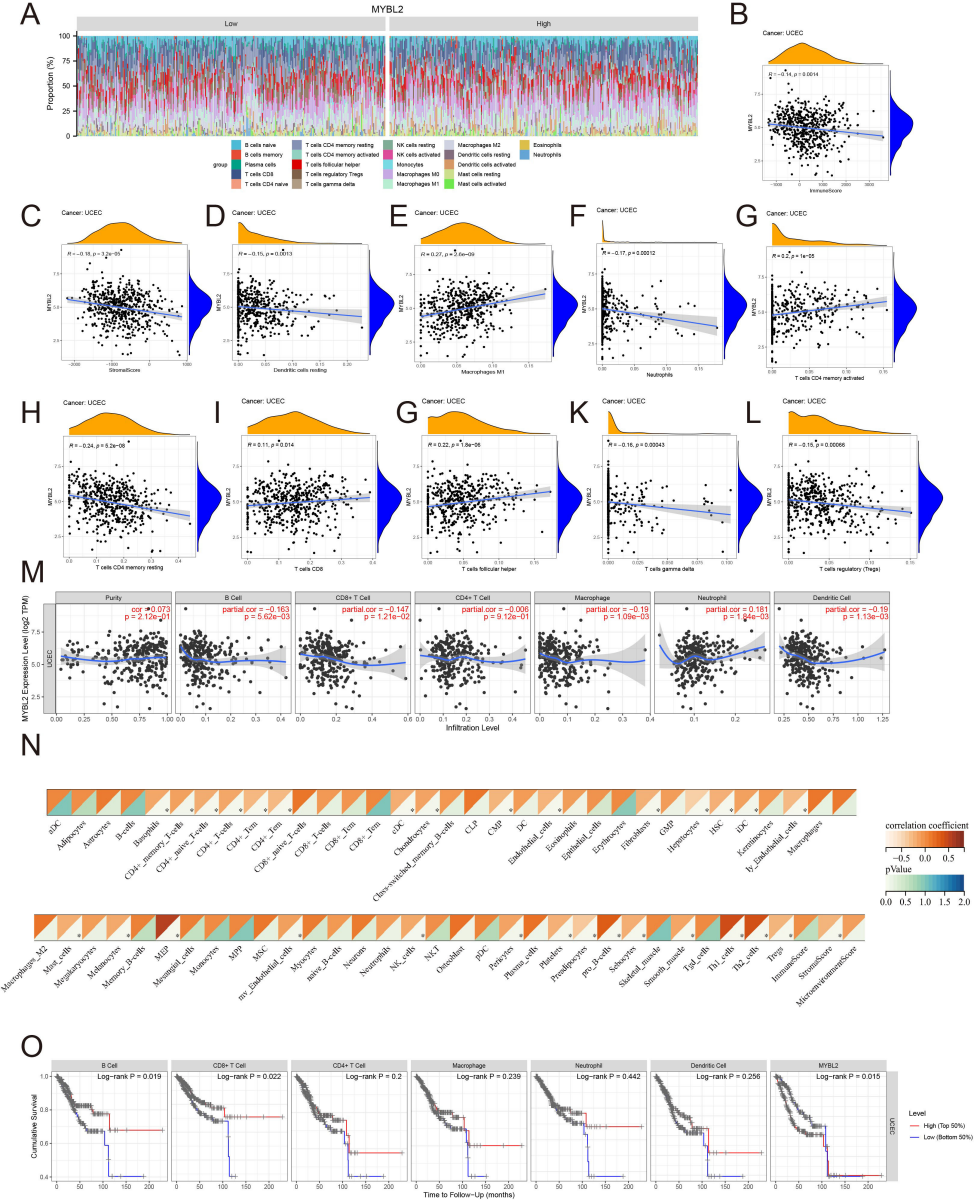
The relationship between MYBL2 and immune infiltration in UCEC. **(A)** Expression of MYBL2 in various immune cell types; **(B, C)**. Correlation between MYBL2 expression and immune and stromal scores; **(D–L)**. Correlations between MYBL2 expression and the infiltration levels of immune cells, as assessed by CIBERSORT; **(M)** Correlation between MYBL2 expression and infiltration levels of six immune cell types in TIMER2.0; **(N)** Correlation between MYBL2 expression and infiltration levels of immune cells by xCELL; **(O)** Cumulative survival of UCEC patients with high or low immune cell infiltration levels in TIMER2.0 for the six immune cell types.

Various approaches were used to further investigate the role of MYBL2 in immune infiltration within the tumor microenvironment of UCEC. The distribution of MYBL2 across different immune cell types was analyzed using CIBERSORT (**Figure 4A**). MYBL2 expression in UCEC was significantly negatively correlated with stromal (r = −0.18, *p* = 3.2e−05) and immune (r = −0.14, *p* = 0.0014) scores (**Figures 4B, C**). Additionally, correlation analysis with CIBERSORT assessed the relationship between MYBL2 expression and the infiltration levels of eight immune cell types (**Figures 4D–L**). MYBL2 expression showed a positive correlation with Macrophages M1 (r = 0.27, *p* = 2.6e−09), activated CD4 memory T cells (r = 0.2, *p* = 1e−05), CD8 T cells (r = 0.11, *p* = 0.014), and follicular helper T cells (r = 0.22, *p* = 1.8e−06). Conversely, MYBL2 expression was negatively correlated with resting dendritic cells (r = −0.15, *p* = 0.0013), neutrophils (r = −0.17, *p* = 0.00012), resting CD4 memory T cells (r = −0.24, *p* = 5.2e−08), gamma T cells (r = −0.16, *p* = 0.00043), and regulatory T cells (Tregs) (r = −0.15, *p* = 0.00066). Further analysis of the correlation between MYBL2 expression and the infiltration levels of six major immune cell types was conducted using TIMER2.0. Notably, MYBL2 expression demonstrated a significant positive correlation with neutrophils (r = 0.181, *p* = 1.84e−03) and purity (r = 0.073, *p* = 2.12e−01). In contrast, MYBL2 expression in UCEC was negatively correlated with B cells (r = −0.163, *p* = 5.62e−03), CD8^+^ T cells (r = −0.147, *p* = 1.21e−02), CD4^+^ T cells (r = −0.006, *p* = 9.12e−01), macrophages (r = −0.19, *p* = 1.09e−03), and dendritic cells (r = −0.19, *p* = 1.13e−03) (**Figure 4M**). Furthermore, the xCELL tool was used to validate the effect of MYBL2 on immune cell infiltration, and the results were consistent(**Figure 4N**).Finally, survival curve analysis revealed that patients with low MYBL2 expression in B cells and CD8+ T cells exhibited a better cumulative survival rate compared to those with high MYBL2 expression (**Figure 4O**).

### 3.5 Associations of MYBL2 expression with immune status related genes and immunotherapy

**Figure 5:**
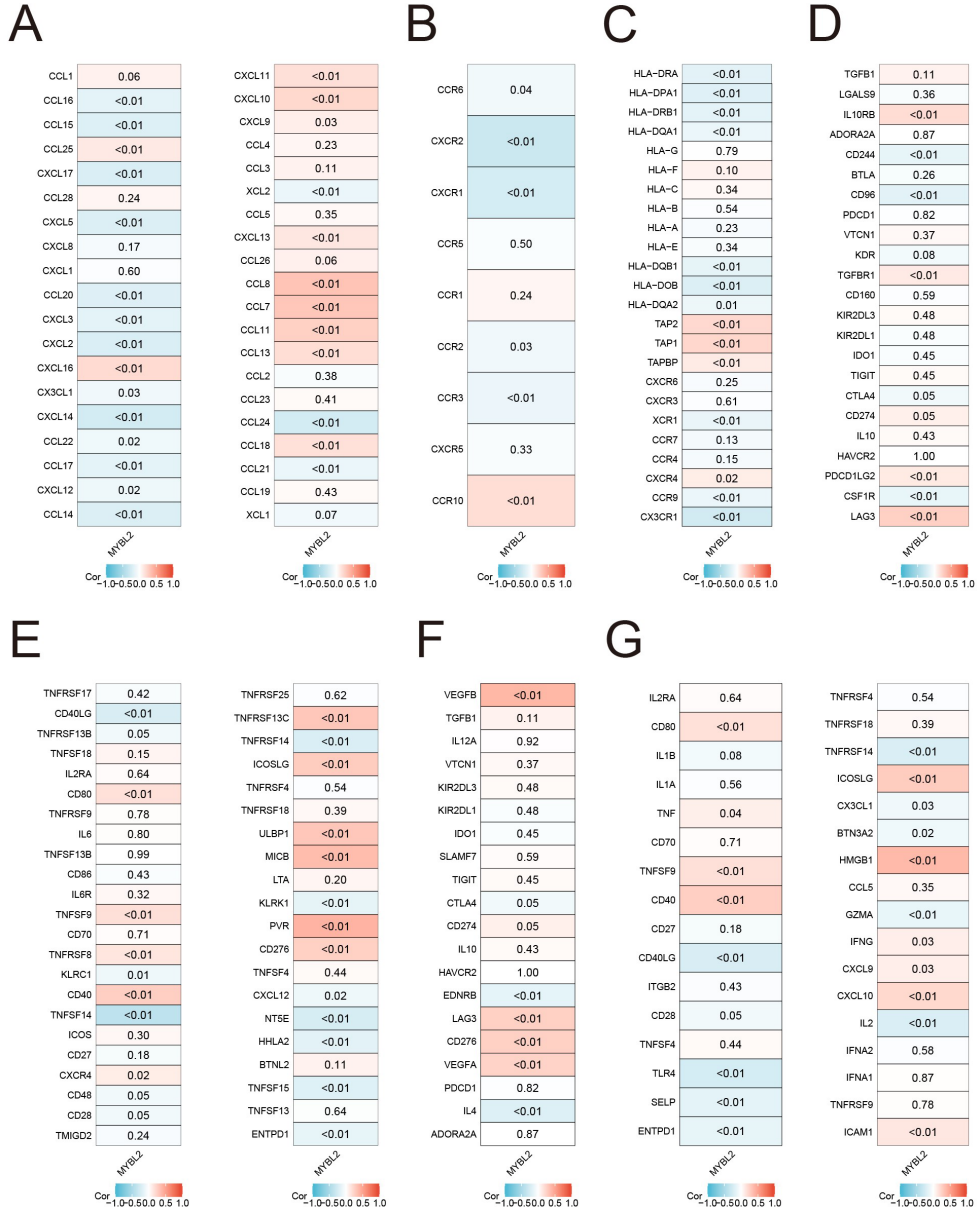
Associations of MYBL2 expression with immune status-related genes and immunotherapy in UCEC. **(A)** chemokines; **(B)** receptors; **(C)** MHC; **(D)** immunoinhibitors; **(E)** immunostimulators; **(F)** immune checkpoint suppressor genes; **(G)** immune checkpoint-promoting genes.

The expression of the MYBL2 gene shows a strong positive correlation with most chemokine genes, as illustrated in **Figure 5A** (e.g., CCL8, CCL7, CCL11). Additionally, the correlation analysis of receptor genes revealed a weaker statistical significance, as shown in **Figure 5B**. Further correlation analysis with MHC genes (**Figure 5C**) revealed a significant positive correlation with certain genes (e.g., TAP2, TAP1, TAPBP), while a significant negative correlation was observed with another set of genes (e.g., HLA-DRA, HLA-DPA1). The correlation analysis of genes encoding immune suppressors and stimulators (**Figures 5D, E**) showed that most of the statistically significant molecules were positively correlated with MYBL2, including IL10RB, TGFBR1, LAG3, CD80, and CD40. Moreover, the investigation of immune checkpoint genes (**Figures 5F, G**) demonstrated that MYBL2 was positively correlated with certain immune checkpoint inhibitors, such as VEGFB and LAG3, as well as immune checkpoint activators, including CD80, TNFSF9, CD40, ICOSLG, HMGB1, and CXCL10.

### 3.6 Pathway enrichment analysis related to MYBL2 expression

**Figure 6:**
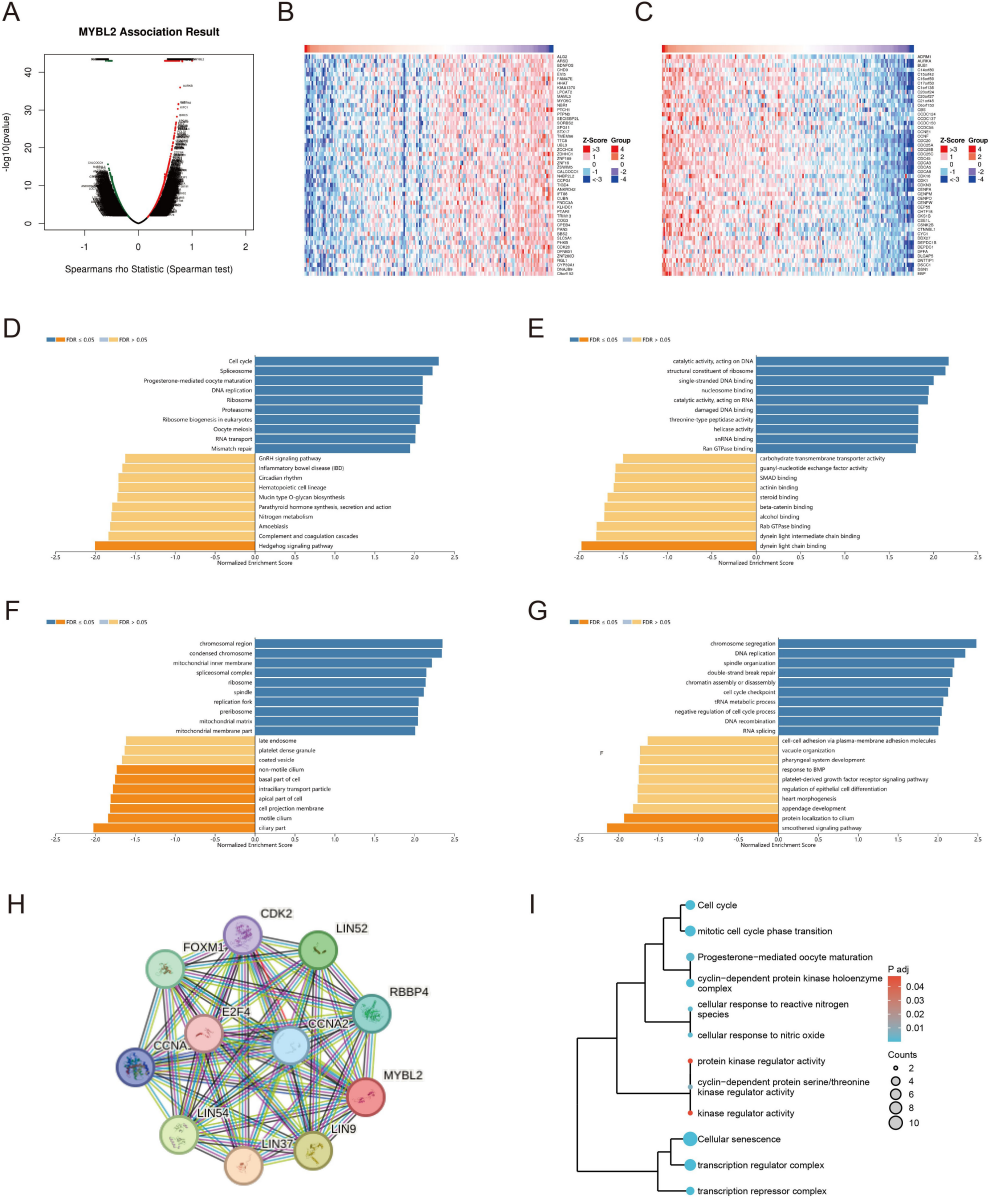
The enrichment analysis related to the MYBL2 gene. **(A)** Correlation of the MYBL2 gene; **(B, C)** Heatmap of high and low differential expression of MYBL2 and related genes; **(D–G)** KEGG/GO enrichment analysis of the interaction and associated protein network of MYBL2; **(H)** Co-expression network of the MYBL2 gene; I, The correlation of MYBL2 with various biological processes and functions.

We conducted a genome-wide transcriptomic gene correlation analysis of the MYBL2 gene in UCEC, and generated a volcano plot (**Figure 6A**) and heatmaps (**Figures 6B, C**). We identified that MYBL2 is positively correlated with genes such as ADRM1, AURKA, and BUB1, and negatively correlated with genes such as ALG2, ARSD, and BDNFOS. Gene Ontology (GO) and Kyoto Encyclopedia of Genes and Genomes (KEGG) pathway enrichment analyses were conducted to identify the functional pathways significantly associated with MYBL2 expression. The Biological Process (BP) pathway analysis in **Figure 6D** revealed that MYBL2 is positively correlated with pathways such as Cell cycle, Spliceosome, and Ribosome. The Cellular Component (CC) pathway analysis in **Figure 6E** indicated that pathways like nuosome binding, catalytic activity acting on DNA, and structural constituent of ribosome are positively correlated with MYBL2. The analysis in **Figure 6F** showed that MYBL2 is positively correlated with pathways such as chromosomal region and condensed chromosome, but negatively correlated with pathways like late endosome and coated vesicle. Finally, the KEGG pathway analysis in **Figure 6G** demonstrated that MYBL2 is negatively correlated with pathways such as chromosome segregation and DNA replication. Based on the central role of MYBL2 in cell cycle regulation and cell proliferation, a gene co-expression network was constructed, as shown in **Figure 6H**. The enrichment analysis presented in **Figure 6I** elucidated the significant roles of MYBL2 in various biological processes and functions, including transcriptional regulation, cell cycle control, cellular senescence, oxidative stress response, and germ cell development.

### 3.7 Prediction of drug sensitivity associated with expression of MYBL2 in UCEC

**Figure 7:**
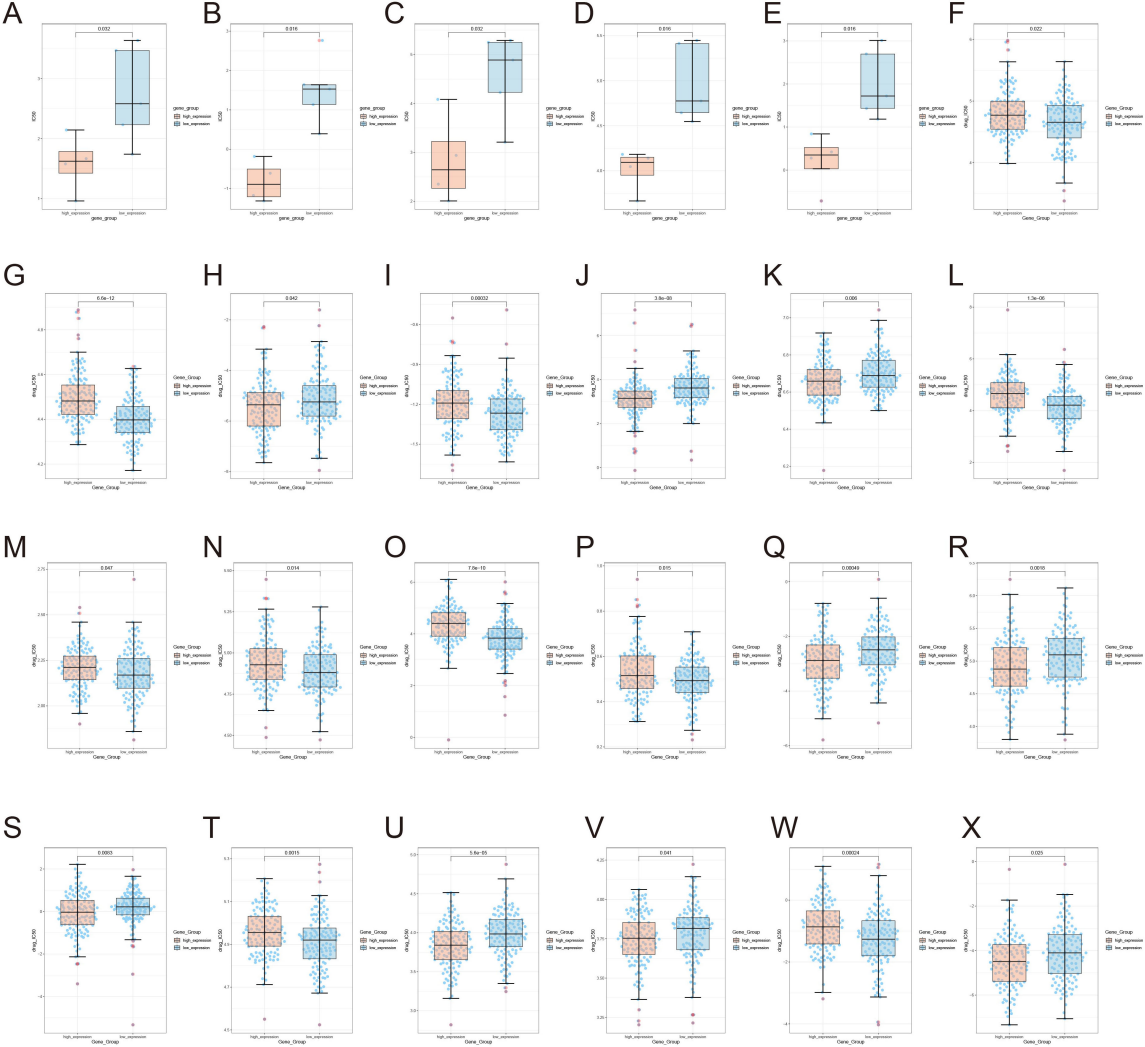
Illustrates the expression of MYBL2 in UCEC tumors under the influence of various drugs. **(A)** Afatinib; **(B)** AZD8055; **(C)** ERK_2440; **(D)** PCI-34051; **(E)** Pictilisib; **(F)** Bexarotene; **(G)** Bicalutamide; **(H)** Bortezomib; **(I)** Bryostatin; **(J)** Cisplatin; **(K)** Cyclopamine; **(L)** Erlotinib; **(M)** Gefitinib; **(N)** Imatinib; **(O)** Lapatinib; **(P)** Midostaurin; **(Q)** Paclitaxel; **(R)** Parthenolide; **(S)** Rapamycin; **(T)** Roscovitine; **(U)** Sorafenib; **(V)** Sunitinib; **(W)** Temsirolimus; **(X)** Thapsigargin.

The present study reveals significant differences in drug sensitivity profiles between MYBL2 samples based on MYBL2 expression levels. Using the TCGA module, genomic and drug sensitivity data for the MYBL2 gene in UCEC tumors were analyzed. It was observed that the high MYBL2 expression group exhibited significantly higher drug sensitivity to Afatinib (*p* = 0.032, **Figure 7A**). Similarly, in the high MYBL2 expression group, higher drug sensitivity was observed for AZD8055 (*p* = 0.016, **Figure 7B**), ERK_2440 (*p* = 0.032, **Figure 7C**), PCI−34051 (*p* = 0.016, **Figure 7D**), and Pictilisib (*p* = 0.016, **Figure 7E**). In contrast, the low MYBL2 expression group showed higher sensitivity to Bexarotene (*p* = 0.022, **Figure 7F**), Bicalutamide (*p* = 6.6e−12, **Figure 7G**), Bortezomib (*p* = 0.042, **Figure 7H**), and Bryostatin.1 (*p* = 0.00032, **Figure 7I**). Additionally, the high MYBL2 expression group displayed higher sensitivity to Cisplatin (*p* = 3.8e−08, **Figure 7J**) and Cyclopamine (*p* = 0.006, **Figure 7K**). However, the low MYBL2 expression group demonstrated greater sensitivity to Erlotinib (*p* = 1.3e−06, **Figure 7L**), Gefitinib (*p* = 0.047, **Figure 7M**), Imatinib (*p* = 0.014, **Figure 7N**), Lapatinib (*p* = 7.8e−10, **Figure 7O**), and Midostaurin (*p* = 0.015, **Figure 7P**). Furthermore, the high MYBL2 expression group exhibited higher sensitivity to Paclitaxel (*p* = 0.00049, **Figure 7Q**), Parthenolide (*p* = 0.0018, **Figure 7R**), and Rapamycin (*p* = 0.0083, **Figure 7S**). In contrast, the MYBL2 low - expression group exhibited higher sensitivity to Roscovitine (P = 0.0015, **Figure 7T**). The MYBL2 high - expression group also displayed greater sensitivity to Sorafenib (P = 5.6e−05, **Figure 7U**) and Sunitinib (P = 0.041, **Figure 7V**). Conversely, Temsirolimus (P = 0.00024, **Figure 7W**) showed higher sensitivity in the low - expression group. Moreover, the MYBL2 high - expression group demonstrated increased sensitivity to Thapsigargin (P = 0.025, **Figure 7X**).

## 4 Discussion

Although most UCEC patients can be diagnosed and treated at an early stage, approximately 15% are diagnosed at a locally advanced or occult metastatic stage, with tumor recurrence due to limited responses to surgery and radiotherapy (28). Targeted therapy and immunotherapy have shown promise in improving the prognosis of endometrial cancer patients (4, 29). Prognostic molecular biomarkers, including HER2, PD-L1, ER, PR, and MMR/MSI, have gained significant attention due to their relevance in clinical practice (30). However, there is still a lack of highly specific and sensitive biomarkers to predict the efficacy of immunotherapy. Therefore, identifying new prognostic molecular markers and exploring novel therapeutic strategies to optimize the management of endometrial cancer is imperative.

This study analyzed the expression of MYBL2 across various tumors and found that MYBL2 was significantly upregulated in 20 different cancer types, consistent with previous findings in breast cancer, hepatocellular carcinoma, cervical squamous cell carcinoma, and prostate cancer (31–34). Furthermore, Kaplan-Meier survival analysis indicated that the high MYBL2 expression group had significantly poorer outcomes in overall survival, disease-specific survival, disease-free interval, and progression-free interval compared to the low expression group, suggesting a close association between high MYBL2 expression and poor prognosis in endometrial cancer. Additional clinical data analysis revealed that MYBL2 expression levels progressively increased with advancing clinical stage and histological grade, which may explain the poorer survival rates observed in patients with high MYBL2 expression. Effective prognostic biomarkers are crucial for clinical management and treatment decision-making. According to the Receiver Operating Characteristic (ROC) curve analysis, MYBL2 demonstrated excellent accuracy in predicting the prognosis of endometrial cancer patients. Therefore, MYBL2 could serve as a prognostic marker for endometrial cancer and is associated with disease stage and survival outcomes.

DNA methylation may exhibit dual roles in tumors (35). We observed that the methylation level of the MYBL2 promoter was higher in normal tissues than in primary tumor tissues. This finding is consistent with previous studies, where methylation of the promoter region prevents transcription factor binding, thereby reducing gene expression. Research by Tianyi Wu et al. (36) found that transcription factors, such as E2F1 and NF-κB, may promote tumorigenesis by binding to the MYBL2 gene promoter. E2F1, through its interaction with the DREAM complex (Dimerization partner, RB-like proteins, E2Fs, and MuvB core), directly binds to the MYBL2 promoter region. During the G1/S phase of the cell cycle, E2F1 acts as an activating E2F, replacing the repressive E2Fs in the DREAM complex (e.g., E2F4 or E2F5), thus activating MYBL2 expression (37). Moreover, studies by Maddalena Frau et al. (37, 38) have shown that MYBL2 can regulate the expression or activity of key proteins, such as PI3K and AKT, further activating the PI3K/AKT signaling pathway. Activation of AKT promotes the phosphorylation of E2F1, enhancing its transcriptional activity and thereby further increasing MYBL2 expression (39). This feedback mechanism creates an interdependent and mutually reinforcing relationship between E2F1, MYBL2 expression, and the PI3K/AKT pathway activity, forming a synergistic oncogenic network that promotes tumor cell proliferation, inhibits apoptosis, and enhances tumor cell invasion and metastasis.

The tumor immune microenvironment has become a major focus in cancer research, encompassing tumor cells, immune cells, and cytokines, which collectively influence the anti-tumor immune response (40). Immune checkpoint inhibitors targeting various immune checkpoints, such as CTLA-4, PD-1, PD-L1, and B7-H4, have been developed for endometrial cancer and have shown efficacy in improving patient survival rates (30, 41, 42). However, immune resistance remains a concern (43), possibly due to the upregulation of immune checkpoint proteins on tumor cells (44). Additionally, immune therapy may lead to various immune-related adverse events (irAEs) (45), which are associated with immune system overactivation, resulting in cytokine storms and organ-specific inflammation or dysfunction, such as thyroid dysfunction caused by the activation of autoreactive T cells (46, 47). The effectiveness of immunotherapy largely depends on the infiltration of immune cells within the TIME, as these cells play a critical role in regulating tumor progression through various mechanisms.

The study explores the expression pattern and immune-related significance of MYBL2 in UCEC, providing a foundation for investigating its potential immunoregulatory role. The focus is on examining the relationship between MYBL2 expression, immune cell infiltration, and checkpoint molecules. MYBL2 expression shows a positive correlation with the infiltration of immune cells (such as M1 macrophages, CD4 memory activated T cells, and follicular helper T cells), suggesting that MYBL2 may partially regulate the recruitment and activation of immune cells in the tumor microenvironment of UCEC. Additionally, high MYBL2 expression is strongly correlated with several immune checkpoint molecules, immune status-related genes, and chemokine-related genes. These findings indicate that MYBL2 may play a key role in modulating the immune microenvironment in UCEC. Specifically, MYBL2 may influence immune cell infiltration and activation by regulating the expression of chemokines, cytokines, and other immune modulators. Furthermore, the association of MYBL2 with immune checkpoint molecules (such as CTLA-4 and PD-1) suggests that MYBL2 may impact immune therapy efficacy by regulating immune cell exhaustion and activation. To validate these hypotheses, further functional studies, including *in vitro* cell culture experiments and *in vivo* animal models, are needed to assess the direct effects of MYBL2 on immune cell function and UCEC progression. These studies will provide crucial insights into the molecular mechanisms of MYBL2’s immunoregulatory role in UCEC and may inform the development of novel immune therapeutic strategies targeting MYBL2.

This study employs bioinformatics techniques to comprehensively investigate the role of MYBL2 in UCEC, demonstrating a correlation between MYBL2 expression and disease prognosis. While the utilization of diverse tools and databases yields informative insights, the study is constrained by several limitations. Notably, the sample size, while sufficient for initial exploration, may not fully represent the broader population, and sampling biases could influence the results. Additionally, the underlying molecular mechanisms by which MYBL2 influences tumor progression and immune evasion are not fully elucidated, requiring functional studies to bridge this gap. Lastly, the correlational nature of the immune infiltration and checkpoint molecule analysis does not establish causality, underscoring the need for knockout or knockdown models to clarify MYBL2’s specific role. These limitations emphasize the need for future research with larger sample sizes, robust validation strategies, and deeper mechanistic inquiries to solidify MYBL2’s potential as a biomarker for immunity and prognosis in UCEC.

## 5 Conclusions

In summary, this study has preliminarily investigated the expression discrepancy, prognostic significance, methylation modification, and immunomodulatory role of MYBL2 in UCEC. The study provides evidence for the overexpression of MYBL2 in tumor tissues of UCEC patients and suggests that it can serve as a prognostic marker for patient survival. Additionally, the methylation level at the cg18266010 locus of MYBL2 shows a statistically significant correlation with survival prognosis, laying the groundwork for future in-depth epigenetics research. There is a strong association between MYBL2 and the immune status of UCEC, particularly its correlation with various immune cells such as Macrophages M1, activated CD4 memory T cells, and follicular helper T cells, highlighting its potential as a biomarker or specific target for immunotherapy in UCEC. The role of MYBL2 in UCEC warrants further in-depth mechanistic investigation.

## Data Availability

The original contributions presented in the study are included in the article/**Supplementary Material**. Further inquiries can be directed to the corresponding author.
