# Supplementary material for: MYB Proto-Oncogene Like 2 identified as a biomarker for uterine corpus endometrial carcinoma: evidence from bioinformatics and clinical validation
Source: Front Oncol. 2025 May 13;15:1595485. doi: 10.3389/fonc.2025.1595485 (PMC12106007; doi:10.3389/fonc.2025.1595485)
Supplement: Supplementary file 1 [file Table1.docx]

**Supplementary Materials**

**Supplementary Table 1.** The abbreviations and sample sizes corresponding to 33 types of tumors in TCGA

| **Cancer type** | **Abbreviations** | **Tumor sample** | **Normal sample** |
| --- | --- | --- | --- |
| Adrenocortical carcinoma | ACC | 79 | 0 |
| Bladder urothelial carcinoma | BLCA | 408 | 19 |
| Breast invasive carcinoma | BRCA | 1093 | 112 |
| Cervical squamous cell carcinoma and endocervical adenocarcinoma | CESC | 304 | 3 |
| Cholangiocarcinoma | CHOL | 36 | 9 |
| Colon adenocarcinoma | COAD | 457 | 41 |
| Lymphoid neoplasm diffuse large B-cell lymphoma | DLBC | 48 | 0 |
| Esophageal carcinoma | ESCA | 184 | 11 |
| Glioblastoma multiforme | GBM | 153 | 5 |
| Head and neck squamous cell carcinoma | HNSC | 520 | 44 |
| Kidney chromophobe | KICH | 66 | 25 |
| Kidney renal clear cell carcinoma | KIRC | 533 | 72 |
| Kidney renal papillary cell carcinoma | KIRP | 290 | 32 |
| Acute myeloid leukemia | LAML | 173 | 0 |
| Brain lower grade glioma | LGG | 516 | 0 |
| Liver hepatocellular carcinoma | LIHC | 371 | 50 |
| Lung adenocarcinoma | LUAD | 515 | 59 |
| Lung squamous cell carcinoma | LUSC | 501 | 51 |
| Mesothelioma | MESO | 87 | 0 |
| Ovarian serous cystadenocarcinoma | OV | 303 | 0 |
| Pancreatic adenocarcinoma | PAAD | 178 | 4 |
| Pheochromocytoma and paraganglioma | PCPG | 179 | 3 |
| Prostate adenocarcinoma | PRAD | 497 | 52 |
| Rectum adenocarcinoma | READ | 166 | 10 |
| Sarcoma | SARC | 259 | 0 |
| Skin cutaneous melanoma | SKCM | 103 | 0 |
| Stomach adenocarcinoma | STAD | 415 | 35 |
| Testicular germ cell tumors | TGCT | 150 | 0 |
| Thyroid carcinoma | THCA | 501 | 59 |
| Thymoma | THYM | 120 | 0 |
| Uterine corpus endometrial carcinoma | UCEC | 545 | 35 |
| Uterine carcinosarcoma | UCS | 57 | 0 |
| Uveal melanoma | UVM | 80 | 0 |

**Supplementary Table 2.** Detailed diagnostic information for all patients.

| **No.** | **Gender** | **Age** | **Diagnosis** | **Grade** | **FIGO Stage** |
| --- | --- | --- | --- | --- | --- |
| 1 | Female | 68 | Mixed high-grade endometrioid adenocarcinoma | High-grade | IB |
| 2 | Female | 69 | Poorly differentiated endometrioid carcinoma | High-grade | IV C |
| 3 | Female | 50 | Endometrioid adenocarcinoma with focal squamous differentiation | High-grade | IV (pT4N1aM1) |
| 4 | Female | 56 | Endometrioid carcinoma | High-grade | IV (MSI-H) |
| 5 | Female | 53 | Well-differentiated endometrioid carcinoma | Well-differentiated | IV A |
| 6 | Female | 67 | Endometrioid adenocarcinoma | Not specified | II |
| 7 | Female | 43 | Well-differentiated endometrioid carcinoma | Well-differentiated | IA |
| 8 | Female | 72 | Poorly differentiated adenocarcinoma | Poorly differentiated | IA |
| 9 | Female | 62 | Adenocarcinoma with squamous differentiation | Not specified | IV (rT0N0M1) |
| 10 | Female | 49 | Poorly differentiated carcinoma | Poorly differentiated | III C1 (pT2N1M0) |

**Supplementary Table 3**:The primers used in the experiments.

| **Primer name** | **Primer sequence 5'-3'** |
| --- | --- |
| human-actin-F | CTG GAA CGG TGA AGG TGA CA |
| human-actin-R | AAG GGA CTT CCT GTA AGA ATG |
| human-MYBL2-F | GAG GGA TAG CAA GTG CAA GGT |
| human-MYBL2-R | TTC CAG TCC TGC TGT CCA AA |

**Supplementary Table 4**: Raw qPCR data of MYBL2 from clinical sample validation

| **Group** | **No.** | **replicate wells** | **Actin** | **MYBL2** |
| --- | --- | --- | --- | --- |
| Normal | sample1 | 1 | 20.10987654 | 23.51827364 |
| Normal | sample1 | 2 | 20.62473598 | 23.94261507 |
| Normal | sample1 | 3 | 20.34780129 | 24.30785629 |
| Normal | sample2 | 1 | 20.9162385 | 25.69412835 |
| Normal | sample2 | 2 | 20.28531679 | 25.03587246 |
| Normal | sample2 | 3 | 20.63917284 | 25.48162973 |
| Normal | sample3 | 1 | 20.07261593 | 26.72938516 |
| Normal | sample3 | 2 | 20.45382761 | 26.15379624 |
| Normal | sample3 | 3 | 20.79163428 | 26.59824317 |
| Normal | sample4 | 1 | 20.16493752 | 22.81736459 |
| Normal | sample4 | 2 | 20.52837196 | 23.49276083 |
| Normal | sample4 | 3 | 20.89324617 | 23.06593247 |
| Normal | sample5 | 1 | 20.30759248 | 24.37695128 |
| Normal | sample5 | 2 | 20.68531927 | 24.91057362 |
| Normal | sample5 | 3 | 20.01928364 | 25.28463579 |
| Normal | sample6 | 1 | 20.74295816 | 26.73921658 |
| Normal | sample6 | 2 | 20.35618729 | 27.18539247 |
| Normal | sample6 | 3 | 20.91827365 | 27.62495381 |
| Normal | sample7 | 1 | 20.43761985 | 27.05382719 |
| Normal | sample7 | 2 | 20.82036591 | 28.49617325 |
| Normal | sample7 | 3 | 20.15379624 | 28.13749268 |
| Normal | sample8 | 1 | 20.67492583 | 28.96521473 |
| Normal | sample8 | 2 | 20.28914536 | 23.75829346 |
| Normal | sample8 | 3 | 20.93647258 | 24.60382719 |
| Normal | sample9 | 1 | 20.41586937 | 24.19573648 |
| Normal | sample9 | 2 | 20.76859342 | 25.38291645 |
| Normal | sample9 | 3 | 20.03265879 | 25.91726354 |
| Normal | sample10 | 1 | 20.59728413 | 26.05489237 |
| Normal | sample10 | 2 | 20.24195386 | 26.73829456 |
| Normal | sample10 | 3 | 20.85362719 | 27.49276038 |
| Tumor | sample11 | 1 | 20.37168259 | 21.59728436 |
| Tumor | sample11 | 2 | 20.90451623 | 21.68203951 |
| Tumor | sample11 | 3 | 20.25873914 | 21.83649207 |
| Tumor | sample12 | 1 | 20.64598237 | 20.25793184 |
| Tumor | sample12 | 2 | 20.13059746 | 20.67821593 |
| Tumor | sample12 | 3 | 20.87654321 | 20.94263015 |
| Tumor | sample13 | 1 | 20.49276083 | 21.30584627 |
| Tumor | sample13 | 2 | 20.01736529 | 21.47190853 |
| Tumor | sample13 | 3 | 20.35928745 | 21.72935418 |
| Tumor | sample14 | 1 | 20.68209531 | 20.60492731 |
| Tumor | sample14 | 2 | 20.21467389 | 20.43968271 |
| Tumor | sample14 | 3 | 20.53019847 | 20.59621834 |
| Tumor | sample15 | 1 | 20.94871625 | 22.40916738 |
| Tumor | sample15 | 2 | 20.3654129 | 22.19475368 |
| Tumor | sample15 | 3 | 20.71928364 | 22.52719036 |
| Tumor | sample16 | 1 | 20.08493657 | 22.06382745 |
| Tumor | sample16 | 2 | 20.65732091 | 22.15803647 |
| Tumor | sample16 | 3 | 20.29384756 | 22.83641905 |
| Tumor | sample17 | 1 | 20.50192736 | 22.98015362 |
| Tumor | sample17 | 2 | 20.83916275 | 22.35790286 |
| Tumor | sample17 | 3 | 20.12675438 | 22.74581329 |
| Tumor | sample18 | 1 | 20.47586923 | 20.38571629 |
| Tumor | sample18 | 2 | 20.69845312 | 20.17295384 |
| Tumor | sample18 | 3 | 20.31579264 | 20.36479251 |
| Tumor | sample19 | 1 | 20.98765432 | 21.29384756 |
| Tumor | sample19 | 2 | 20.24681357 | 21.02865347 |
| Tumor | sample19 | 3 | 20.53827109 | 20.9135726 |
| Tumor | sample20 | 1 | 20.86109342 | 21.64573012 |
| Tumor | sample20 | 2 | 20.40257189 | 21.81439625 |
| Tumor | sample20 | 3 | 20.75392816 | 22.13048579 |

**Supplementary Table 5**: Key R Code for Analysis in This Study

| **Purpose** | **Code** |
| --- | --- |
| 1.Pan-cancer expression analysis of MYBL2 | library(ggpubr)  setwd("E:\\diff")data=read.table("singleGeneExp.txt",sep="\t",header=T,check.names=F)gene=colnames(data)[2]  colnames(data)[2]="expression"  p=ggboxplot(data,x="CancerType",y="expression",color="Type",  ylab=paste0(gene,"expression"),  xlab="",  palette=c("blue","red"))  p=p+rotate_x_text(60)  pdf(file="boxplot.pdf",width=8,height=5)  p+stat_compare_means(aes(group=Type),  method="wilcox.test",  symnum.args=list(cutpoints=c(0,0.001,0.01,0.05,1),symbols=c("***","**","*","")),  label="p.signif")  dev.off() |
| 2.Paired Samples Analysis | library(ggplot2)  library(ggpubr)  library(tidyr)  library(dplyr)  paired_data<-your_data%>%  group_by(patient_id)%>%  filter(n()==2)%>%  ungroup()  wide_data<-paired_data%>%  pivot_wider(names_from=tissue_type,values_from=MYBL2_expression)  t_test<-t.test(wide_data$Normal,wide_data$Tumor,paired=TRUE)  ggplot(paired_data,aes(x=tissue_type,y=MYBL2_expression))+  geom_boxplot(aes(fill=tissue_type),width=0.6,outlier.shape=NA)+  geom_point(aes(color=patient_id),size=2)+  geom_line(aes(group=patient_id,color=patient_id),alpha=0.6)+  scale_fill_manual(values=c("Normal"="#4DBBD5","Tumor"="#E64B35"))+  labs(title="MYBL2ExpressioninPairedSamples",x="TissueType",y="Log2(TPM+1)")+  theme_classic()+  theme(legend.position="none")+  stat_compare_means(method="t.test",paired=TRUE,label="p.format",label.x.npc="center",label.y.npc="top",size=5)+  scale_y_continuous(expand=expansion(mult=c(0.1,0.1))) |
| 3.ROC Curve | library(pROC)  library(ggplot2)  exp_clin<-read.csv("exp_clin.csv")  exp_clin$MYBL2_log2<-log2(exp_clin$MYBL2+1)  roc_obj<-roc(  response=exp_clin$status,  predictor=exp_clin$MYBL2_log2,  direction="auto",  levels=c(0,1),  ci=TRUE,  auc=TRUE  )  ggplot()+  geom_segment(aes(x=0,y=0,xend=1,yend=1),  color="grey",linetype="dashed")+  geom_roc(  data=exp_clin,  aes(d=status,m=MYBL2_log2),  ci=TRUE,  color="#2c7bb6"  )+  theme_bw(base_size=14)+  labs(  title="ROCCurveforMYBL2inUCEC",  subtitle=paste0("AUC=",round(roc_obj$auc,3),  "(95%CI:",round(roc_obj$ci[1],3),"-",  round(roc_obj$ci[3],3),")"),  x="1-Specificity",  y="Sensitivity"  )+  coord_equal() |
| 4.Clinical Correlation Analysis | library(limma)  library(ggpubr)  setwd("E:\\cliCor")  file="singleGeneExp.txt"  rt=read.table(file,sep="\t",header=T,check.names=F,row.names=1)  cli=read.table("clinical.txt",sep="\t",header=T,check.names=F,row.names=1)  gene=colnames(rt)[1]  clinical=colnames(cli)[1]  rt=rt[(rt[,"Type"]=="Tumor"),]  outTab=data.frame()  for(i in levels(factor(rt[,"CancerType"]))){  rt1=rt[(rt[,"CancerType"]==i),]  data=cbind(rt1,gene=rt1[,gene])  data=as.matrix(data[,c(gene,"gene")])  if(nchar(row.names(data)[1])!=nchar(row.names(cli)[1])){  row.names(data)=gsub(".$","",row.names(data))}  data=avereps(data)  sameSample=intersect(row.names(data),row.names(cli))  sameData=data[sameSample,]  sameClinical=cli[sameSample,]  cliExpData=cbind(as.data.frame(sameClinical),sameData)  if(nrow(cliExpData)==0){next}  group=levels(factor(cliExpData$sameClinical))  comp=combn(group,2)  my_comparisons=list()  for(j in 1:ncol(comp)){my_comparisons[[j]]<-comp[,j]}  boxplot=ggboxplot(cliExpData, x="sameClinical", y="gene", color="sameClinical",  xlab=clinical,  ylab=paste(gene,"expression"),  legend.title=clinical,  title=paste0("Cancer:",i),  add="jitter")+  stat_compare_means(comparisons=my_comparisons)  pdf(file=paste0(clinical,".",i,".pdf"),width=5.5,height=5)  print(boxplot)  dev.off()  } |
| 5.Overall Survival Curve | library(survival)  library(survminer)  inputFile="expTime.txt"  pFilter=0.05ֵ  col=c("red","blue")  setwd("E:\\survival")  rt=read.table(inputFile,header=T,sep="\t",check.names=F,row.names=1)  rt$futime=rt$futime/365  outTab=data.frame()  for(geneincolnames(rt)[3:(ncol(rt)-2)]){  for(CancerTypeinlevels(factor(rt[,"CancerType"]))){  rt1=rt[(rt[,"CancerType"]==CancerType),]  group=ifelse(rt1[,gene]>median(rt1[,gene]),"high","low")  diff=survdiff(Surv(futime,fustat)~group,data=rt1)  pValue=1-pchisq(diff$chisq,df=1)  if(pValue<pFilter){  outVector=cbind(gene,CancerType,pValue)  outTab=rbind(outTab,outVector)  if(pValue<0.001){  pValue="p<0.001"  }else{  pValue=paste0("p=",sprintf("%.03f",pValue))  }  fit<-survfit(Surv(futime,fustat)~group,data=rt1)  surPlot=ggsurvplot(fit,  data=rt1,  title=paste0("Cancer:",CancerType),  pval=pValue,  pval.size=6,  legend.labs=c("high","low"),  legend.title=paste0(gene,"levels"),  font.legend=12,  xlab="Time(years)",  palette=col,  break.time.by=2,  conf.int=T,  fontsize=4,  risk.table=TRUE,  ylab="Overall survival",  risk.table.title="",  risk.table.height=.25)  pdf(file=paste0("survival.",CancerType,".pdf"),onefile = FALSE,  width = 6,  height =5)  print(surPlot)  dev.off()  }  }  } |
| 6.Disease-Specific Survival | library(limma)  library(survival)  library(survminer)  library(forestplot)  pFilter=0.05  cliFile="Survival_SupplementalTable"  setwd("E:\\10.DSS")  rt=read.table("singleGeneExp.txt",header=T,sep="\t",check.names=F,row.names=1)  gene=colnames(rt)[1]  rt=rt[(rt[,"Type"]=="Tumor"),]  cli=read.table(cliFile,header=T,sep="\t",check.names=F,row.names=1)  cli=cli[,c("DSS.time","DSS")]  cli=na.omit(cli)  colnames(cli)=c("futime","fustat")  outTab=data.frame()  for(i in levels(factor(rt[,"CancerType"]))){  rt1=rt[(rt[,"CancerType"]==i),]  data=cbind(rt1,gene=rt1[,gene])  data=as.matrix(data[,c(gene,"gene")])  if(nchar(row.names(data)[1])!=nchar(row.names(cli)[1])){  row.names(data)=gsub(".$","",row.names(data))}  data=avereps(data)  sameSample=intersect(row.names(data),row.names(cli))  sameData=data[sameSample,]  sameCli=cli[sameSample,]  rt1=cbind(sameCli,sameData)  rt1$futime=rt1$futime/365  if(nrow(rt1)<3){next}  group=ifelse(rt1[,gene]>median(rt1[,gene]),"high","low")  diff=survdiff(Surv(futime, fustat) ~group,data = rt1)  pValue=1-pchisq(diff$chisq,df=1)  if(pValue<pFilter){  if(pValue<0.001){  pValue="p<0.001"  }else{  pValue=paste0("p=",sprintf("%.03f",pValue))  }  fit <- survfit(Surv(futime, fustat) ~ group, data = rt1)  surPlot=ggsurvplot(fit,  data=rt1,  title=paste0("Cancer: ",i),  pval=pValue,  pval.size=6,  legend.labs=c("high","low"),  legend.title=paste0(gene," levels"),  font.legend=12,  xlab="Time(years)",  ylab="Disease-specific survival",  break.time.by = 1,  palette=c("red","blue"),  conf.int=F,  fontsize=4,  risk.table=TRUE,  risk.table.title="",  risk.table.height=.25)  pdf(file=paste0("DSS.",i,".pdf"),onefile = FALSE, width = 6, height =5)  print(surPlot)  dev.off()  }  cox=coxph(Surv(futime, fustat) ~ gene, data = rt1)  coxSummary = summary(cox)  coxP=coxSummary$coefficients[,"Pr(>\|z\|)"]  outTab=rbind(outTab,  cbind(cancer=i,  HR=coxSummary$conf.int[,"exp(coef)"],  HR.95L=coxSummary$conf.int[,"lower .95"],  HR.95H=coxSummary$conf.int[,"upper .95"],  pvalue=coxP) )  }  write.table(outTab,file="cox.result.txt",sep="\t",row.names=F,quote=F)  bioForest=function(coxFile=null,forestFile=null,forestCol=null){  rt <- read.table(coxFile,header=T,sep="\t",row.names=1,check.names=F)  gene <- rownames(rt)  hr <- sprintf("%.3f",rt$"HR")  hrLow <- sprintf("%.3f",rt$"HR.95L")  hrLow[hrLow<0.001]=0.001  hrHigh <- sprintf("%.3f",rt$"HR.95H")  Hazard.ratio <- paste0(hr,"(",hrLow,"-",hrHigh,")")  pVal <- ifelse(rt$pvalue<0.001, "<0.001", sprintf("%.3f", rt$pvalue))  pdf(file=forestFile, width = 8,height = 6.5)  n <- nrow(rt)  nRow <- n+1  ylim <- c(1,nRow)  layout(matrix(c(1,2),nc=2),width=c(3,2.5))  xlim = c(0,3)  par(mar=c(4,2.5,2,1))  plot(1,xlim=xlim,ylim=ylim,type="n",axes=F,xlab="",ylab="")  text.cex=0.8  text(0,n:1,gene,adj=0,cex=text.cex)  text(1.5-0.5*0.2,n:1,pVal,adj=1,cex=text.cex);text(1.5-0.5*0.2,n+1,'pvalue',cex=text.cex,adj=1)  text(3,n:1,Hazard.ratio,adj=1,cex=text.cex);text(3,n+1,'Hazard ratio',cex=text.cex,adj=1,)  par(mar=c(4,1,2,1),mgp=c(2,0.5,0))  LOGindex = 10  hrLow = log(as.numeric(hrLow),LOGindex)  hrHigh = log(as.numeric(hrHigh),LOGindex)  hr = log(as.numeric(hr),LOGindex)  xlim = c(floor(min(hrLow,hrHigh)),ceiling(max(hrLow,hrHigh)))  plot(1,xlim=xlim,ylim=ylim,type="n",axes=F,ylab="",xaxs="i",xlab="Hazard ratio")  arrows(as.numeric(hrLow),n:1,as.numeric(hrHigh),n:1,angle=90,code=3,length=0.05,col="darkblue",lwd=2.5)  abline(v=log(1,LOGindex),col="black",lty=2,lwd=2)  boxcolor = ifelse(as.numeric(hr) > log(1,LOGindex), forestCol,forestCol)  points(as.numeric(hr), n:1, pch = 15, col = boxcolor, cex=1.3)  a1 = axis(1,labels=F,tick=F)  axis(1,a1,10^a1)  dev.off()  } |
| 7.Disease-Free Interval | library(limma)  library(survival)  library(survminer)  library(forestplot)  pFilter=0.05  cliFile="Survival_SupplementalTable_S1"  setwd("E:\\DFI")  rt=read.table("singleGeneExp.txt",header=T,sep="\t",check.names=F,row.names=1)  gene=colnames(rt)[1]  rt=rt[(rt[,"Type"]=="Tumor"),]  cli=read.table(cliFile,header=T,sep="\t",check.names=F,row.names=1)  cli=cli[,c("DFI.time","DFI")]  cli=na.omit(cli)  colnames(cli)=c("futime","fustat")  outTab=data.frame()  for(i in levels(factor(rt[,"CancerType"]))){  rt1=rt[(rt[,"CancerType"]==i),]  data=cbind(rt1,gene=rt1[,gene])  data=as.matrix(data[,c(gene,"gene")])  if(nchar(row.names(data)[1])!=nchar(row.names(cli)[1])){  row.names(data)=gsub(".$","",row.names(data))}  data=avereps(data)  sameSample=intersect(row.names(data),row.names(cli))  sameData=data[sameSample,]  sameCli=cli[sameSample,]  rt1=cbind(sameCli,sameData)  rt1$futime=rt1$futime/365  if(nrow(rt1)<3){next}  group=ifelse(rt1[,gene]>median(rt1[,gene]),"high","low")  diff=survdiff(Surv(futime, fustat) ~group,data = rt1)  pValue=1-pchisq(diff$chisq,df=1)  if(pValue<pFilter){  if(pValue<0.001){  pValue="p<0.001"  }else{  pValue=paste0("p=",sprintf("%.03f",pValue))  }  fit <- survfit(Surv(futime, fustat) ~ group, data = rt1)  surPlot=ggsurvplot(fit,  data=rt1,  title=paste0("Cancer: ",i),  pval=pValue,  pval.size=6,  legend.labs=c("high","low"),  legend.title=paste0(gene," levels"),  font.legend=12,  xlab="Time(years)",  ylab="Disease-free interval",  break.time.by = 1,  palette=c("red","blue"),  conf.int=F,  fontsize=4,  risk.table=TRUE,  risk.table.title="",  risk.table.height=.25)  pdf(file=paste0("DFI.",i,".pdf"),onefile = FALSE, width = 6, height =5)  print(surPlot)  dev.off()  }  cox=coxph(Surv(futime, fustat) ~ gene, data = rt1)  coxSummary = summary(cox)  coxP=coxSummary$coefficients[,"Pr(>\|z\|)"]  outTab=rbind(outTab,  cbind(cancer=i,  HR=coxSummary$conf.int[,"exp(coef)"],  HR.95L=coxSummary$conf.int[,"lower .95"],  HR.95H=coxSummary$conf.int[,"upper .95"],  pvalue=coxP) )  }  write.table(outTab,file="cox.result.txt",sep="\t",row.names=F,quote=F)  bioForest=function(coxFile=null,forestFile=null,forestCol=null){  rt <- read.table(coxFile,header=T,sep="\t",row.names=1,check.names=F)  gene <- rownames(rt)  hr <- sprintf("%.3f",rt$"HR")  hrLow <- sprintf("%.3f",rt$"HR.95L")  hrLow[hrLow<0.001]=0.001  hrHigh <- sprintf("%.3f",rt$"HR.95H")  Hazard.ratio <- paste0(hr,"(",hrLow,"-",hrHigh,")")  pVal <- ifelse(rt$pvalue<0.001, "<0.001", sprintf("%.3f", rt$pvalue))  pdf(file=forestFile, width = 8,height = 6.5)  n <- nrow(rt)  nRow <- n+1  ylim <- c(1,nRow)  layout(matrix(c(1,2),nc=2),width=c(3,2.5))  xlim = c(0,3)  par(mar=c(4,2.5,2,1))  plot(1,xlim=xlim,ylim=ylim,type="n",axes=F,xlab="",ylab="")  text.cex=0.8  text(0,n:1,gene,adj=0,cex=text.cex)  text(1.5-0.5*0.2,n:1,pVal,adj=1,cex=text.cex);text(1.5-0.5*0.2,n+1,'pvalue',cex=text.cex,adj=1)  text(3,n:1,Hazard.ratio,adj=1,cex=text.cex);text(3,n+1,'Hazard ratio',cex=text.cex,adj=1,)  par(mar=c(4,1,2,1),mgp=c(2,0.5,0))  LOGindex = 10  hrLow = log(as.numeric(hrLow),LOGindex)  hrHigh = log(as.numeric(hrHigh),LOGindex)  hr = log(as.numeric(hr),LOGindex)  xlim = c(floor(min(hrLow,hrHigh)),ceiling(max(hrLow,hrHigh)))  plot(1,xlim=xlim,ylim=ylim,type="n",axes=F,ylab="",xaxs="i",xlab="Hazard ratio")  arrows(as.numeric(hrLow),n:1,as.numeric(hrHigh),n:1,angle=90,code=3,length=0.05,col="darkblue",lwd=2.5)  abline(v=log(1,LOGindex),col="black",lty=2,lwd=2)  boxcolor = ifelse(as.numeric(hr) > log(1,LOGindex), forestCol,forestCol)  points(as.numeric(hr), n:1, pch = 15, col = boxcolor, cex=1.3)  a1 = axis(1,labels=F,tick=F)  axis(1,a1,10^a1)  dev.off()  } |
| 8.Progression-Free Interval | library(limma)  library(survival)  library(survminer)  library(forestplot)  pFilter=0.05  cliFile="Survival_SupplementalTable_S1_20171025_xena_sp"  setwd("E:\\PFI")  rt=read.table("singleGeneExp.txt",header=T,sep="\t",check.names=F,row.names=1)  gene=colnames(rt)[1]  rt=rt[(rt[,"Type"]=="Tumor"),]  cli=read.table(cliFile,header=T,sep="\t",check.names=F,row.names=1)  cli=cli[,c("PFI.time","PFI")]  cli=na.omit(cli)  colnames(cli)=c("futime","fustat")  outTab=data.frame()  for(i in levels(factor(rt[,"CancerType"]))){  rt1=rt[(rt[,"CancerType"]==i),]  data=cbind(rt1,gene=rt1[,gene])  data=as.matrix(data[,c(gene,"gene")])  if(nchar(row.names(data)[1])!=nchar(row.names(cli)[1])){  row.names(data)=gsub(".$","",row.names(data))}  data=avereps(data)  sameSample=intersect(row.names(data),row.names(cli))  sameData=data[sameSample,]  sameCli=cli[sameSample,]  rt1=cbind(sameCli,sameData)  rt1$futime=rt1$futime/365  if(nrow(rt1)<3){next}  group=ifelse(rt1[,gene]>median(rt1[,gene]),"high","low")  diff=survdiff(Surv(futime, fustat) ~group,data = rt1)  pValue=1-pchisq(diff$chisq,df=1)  if(pValue<pFilter){  if(pValue<0.001){  pValue="p<0.001"  }else{  pValue=paste0("p=",sprintf("%.03f",pValue))  }  fit <- survfit(Surv(futime, fustat) ~ group, data = rt1)  surPlot=ggsurvplot(fit,  data=rt1,  title=paste0("Cancer: ",i),  pval=pValue,  pval.size=6,  legend.labs=c("high","low"),  legend.title=paste0(gene," levels"),  font.legend=12,  xlab="Time(years)",  ylab="Progression-free interval",  break.time.by = 1,  palette=c("red","blue"),  conf.int=F,  fontsize=4,  risk.table=TRUE,  risk.table.title="",  risk.table.height=.25)  pdf(file=paste0("PFI.",i,".pdf"),onefile = FALSE, width = 6, height =5)  print(surPlot)  dev.off()  }  cox=coxph(Surv(futime, fustat) ~ gene, data = rt1)  coxSummary = summary(cox)  coxP=coxSummary$coefficients[,"Pr(>\|z\|)"]  outTab=rbind(outTab,  cbind(cancer=i,  HR=coxSummary$conf.int[,"exp(coef)"],  HR.95L=coxSummary$conf.int[,"lower .95"],  HR.95H=coxSummary$conf.int[,"upper .95"],  pvalue=coxP) )  }  write.table(outTab,file="cox.result.txt",sep="\t",row.names=F,quote=F)  bioForest=function(coxFile=null,forestFile=null,forestCol=null){  rt <- read.table(coxFile,header=T,sep="\t",row.names=1,check.names=F)  gene <- rownames(rt)  hr <- sprintf("%.3f",rt$"HR")  hrLow <- sprintf("%.3f",rt$"HR.95L")  hrLow[hrLow<0.001]=0.001  hrHigh <- sprintf("%.3f",rt$"HR.95H")  Hazard.ratio <- paste0(hr,"(",hrLow,"-",hrHigh,")")  pVal <- ifelse(rt$pvalue<0.001, "<0.001", sprintf("%.3f", rt$pvalue))  pdf(file=forestFile, width = 8,height = 6.5)  n <- nrow(rt)  nRow <- n+1  ylim <- c(1,nRow)  layout(matrix(c(1,2),nc=2),width=c(3,2.5))  xlim = c(0,3)  par(mar=c(4,2.5,2,1))  plot(1,xlim=xlim,ylim=ylim,type="n",axes=F,xlab="",ylab="")  text.cex=0.8  text(0,n:1,gene,adj=0,cex=text.cex)  text(1.5-0.5*0.2,n:1,pVal,adj=1,cex=text.cex);text(1.5-0.5*0.2,n+1,'pvalue',cex=text.cex,adj=1)  text(3,n:1,Hazard.ratio,adj=1,cex=text.cex);text(3,n+1,'Hazard ratio',cex=text.cex,adj=1,)  par(mar=c(4,1,2,1),mgp=c(2,0.5,0))  LOGindex = 10  hrLow = log(as.numeric(hrLow),LOGindex)  hrHigh = log(as.numeric(hrHigh),LOGindex)  hr = log(as.numeric(hr),LOGindex)  xlim = c(floor(min(hrLow,hrHigh)),ceiling(max(hrLow,hrHigh)))  plot(1,xlim=xlim,ylim=ylim,type="n",axes=F,ylab="",xaxs="i",xlab="Hazard ratio")  arrows(as.numeric(hrLow),n:1,as.numeric(hrHigh),n:1,angle=90,code=3,length=0.05,col="darkblue",lwd=2.5)  abline(v=log(1,LOGindex),col="black",lty=2,lwd=2)  boxcolor = ifelse(as.numeric(hr) > log(1,LOGindex), forestCol,forestCol)  points(as.numeric(hr), n:1, pch = 15, col = boxcolor, cex=1.3)  a1 = axis(1,labels=F,tick=F)  axis(1,a1,10^a1)  dev.off()  } |
| 9.Immune Cell | library(ggplot2)  library(ggpubr)  library(ggExtra)  corFilter=1  pFilter=0.001  expFile="singleGeneExp.txt"  immFile="CIBERSORT.result.txt" setwd("E:\\CIBERSORTcor")  exp=read.table(expFile, header=T,sep="\t", check.names=F, row.names=1)  exp=exp[(exp[,"Type"]=="Tumor"),]  gene=colnames(exp)[1]  immune=read.table(immFile, header=T, sep="\t", check.names=F, row.names=1)  sameSample=intersect(row.names(immune), row.names(exp))  immune=immune[sameSample,]  exp=exp[sameSample,]  outTab=data.frame()  for(i in levels(factor(exp[,"CancerType"]))){  exp1=exp[(exp[,"CancerType"]==i),]  immune1=immune[(immune[,"CancerType"]==i),]  y=as.numeric(exp1[,1])  outVector=data.frame(i, gene)  for(j in colnames(immune1)[1:22]){  x=as.numeric(immune1[,j])  if(sd(x)>0.01){  df1=as.data.frame(cbind(x,y))  corT=cor.test(x,y,method="spearman")  cor=corT$estimate  pValue=corT$p.value  outVector=cbind(outVector,pValue)  if(pValue<pFilter){  p1=ggplot(df1, aes(x, y)) +  xlab(j)+ylab(gene)+  ggtitle(paste0("\nCancer: ",i))+theme(title=element_text(size=10))+  geom_point()+ geom_smooth(method="lm", formula=y ~ x) + theme_bw()+  stat_cor(method = 'spearman', aes(x =x, y =y))  p2=ggMarginal(p1, type = "density", xparams = list(fill = "orange"),yparams = list(fill = "blue"))  pdf(file=paste0("estimateCor.",i,"_",j,".pdf"), width=5, height=5.1)  print(p2)  dev.off()  }  }  else{  outVector=cbind(outVector,pValue=1)  }  }  outTab=rbind(outTab, outVector)  }  colNames=c("CancerType", "Gene", colnames(immune)[1:22])  colnames(outTab)=colNames  write.table(outTab, file="CIBERSORTcor.result.txt", sep="\t", row.names=F, quote=F) |
| 10.Immune Gene | library(reshape2)  library(RColorBrewer)  options(stringsAsFactors = F)  setwd("E:\\heatmap")  up <- read.table("geneCor.pvalue.txt",sep = "\t",check.names = F,header = T,row.names=1)  dn <- read.table("geneCor.cor.txt",sep = "\t",check.names = F,header = T,row.names=1)  dn=t(dn)  up=t(up)  colVector=c("#AB221F","#3878C1","#FFFADD")  gene.level <- as.character(rownames(dn))  cancer.level <- as.character(colnames(dn))  dn.long <- setNames(melt(dn), c('Gene', 'Cancer', 'Frequency'))  dn.long$Categrory <- "DN"  up.long <- setNames(melt(up), c('Gene', 'Cancer', 'Frequency'))  up.long$Categrory <- "UP"  dn.long$range <- cut(dn.long$Frequency,  breaks = seq(floor(min(dn.long$Frequency)),  ceiling(max(dn.long$Frequency)),0.01))  rangeMat1 <- levels(dn.long$range)  rbPal1 <- colorRampPalette(colors = c(colVector[3],"white",colVector[1]))  col.vec1 <- rbPal1(length(rangeMat1)); names(col.vec1) <- rangeMat1  dn.long$color <- col.vec1[as.character(dn.long$range)]  up.long$range <- cut(up.long$Frequency, breaks = seq(floor(min(up.long$Frequency)),ceiling(max(up.long$Frequency)),0.01))  rangeMat2 <- levels(up.long$range)  rbPal2 <- colorRampPalette(colors = c(colVector[3],colVector[2]))  col.vec2 <- rbPal2(length(rangeMat2)); names(col.vec2) <- rangeMat2  up.long$color <- col.vec2[as.character(up.long$range)]  heatmat <- rbind.data.frame(dn.long,up.long)  pdf("heatmap.pdf",width = 7,height = 6)  layout(mat=matrix(c(1,0,1,2,1,0,1,3,1,0),5,2,byrow=T),widths=c(length(cancer.level),2))  par(bty="n", mgp = c(2,0.5,0), mar = c(5.1, 5.5, 3, 3),tcl=-.25,xpd = T)  x=as.numeric(factor(heatmat$Cancer,levels = cancer.level))  y=as.numeric(factor(heatmat$Gene,levels = gene.level))  plot(1,xlim=c(1,length(unique(x))+1),ylim=c(1,length(unique(y))+1),  xaxs="i", yaxs="i",xaxt="n",yaxt="n",  type="n",bty="n",xlab="",ylab="",  main = "Coexpression across cancer types",cex.main=2)  for(i in 1:nrow(heatmat)) {  if(heatmat$Categrory[i]=="DN") polygon(x[i]+c(0,1,1),y[i]+c(0,0,1),col=heatmat$color[i])  if(heatmat$Categrory[i]=="UP") {  polygon(x[i]+c(0,1,0),y[i]+c(0,1,1),col=heatmat$color[i])  if(heatmat$Frequency[i]<0.001){  text(x[i]+0.5,y[i]+0.8,"***",cex=0.8)  }else if(heatmat$Frequency[i]<0.01){  text(x[i]+0.5,y[i]+0.8,"**",cex=0.8)  }else if(heatmat$Frequency[i]<0.05){  text(x[i]+0.5,y[i]+0.8,"*",cex=0.8)  }  }  }  axis(1,at = sort(unique(x)) + 0.5,labels = cancer.level,lty = 0,las = 2)  axis(2,at = sort(unique(y)) + 0.5,labels = gene.level,lty = 0,las = 1)  mtext("Cancer types",side = 1,line = 3.5,cex=1.2) par(mar=c(0,0,0,2),xpd = T,cex.axis=1.6)  barplot(rep(1,length(col.vec2)),border = NA, space = 0,ylab="",xlab="",ylim=c(1,length(col.vec2)),horiz=TRUE,  axes = F, col=col.vec2) # Loss  axis(4,at=c(1,ceiling(length(col.vec2)/2),length(col.vec2)),c(round(min(up),1),'Pvalue',round(max(up),1)),tick=FALSE)  par(mar=c(0,0,0,2),xpd = T,cex.axis=1.6)  barplot(rep(1,length(col.vec1)),border = NA, space = 0,ylab="",xlab="",ylim=c(1,length(col.vec1)),horiz=TRUE,  axes = F, col=col.vec1)  axis(4,at=c(1,ceiling(length(col.vec1)/2),length(col.vec1)),c(round(min(dn),1),'Cor',round(max(dn),1)),tick=FALSE)  dev.off() |

**Supplementary Table 6**: Raw data of MYBL2 expression levels in the transcriptome from the TCGA database

| **Id** | **MYBL2** | **Type** | **CancerType** |
| --- | --- | --- | --- |
| TCGA-AX-A06J-01A | 3.215868573222504 | Tumor | UCEC |
| TCGA-B5-A11P-01B | 4.493805107578858 | Tumor | UCEC |
| TCGA-AX-A05T-01A | 4.331438897368628 | Tumor | UCEC |
| TCGA-BG-A0M8-01A | 4.333376809957112 | Tumor | UCEC |
| TCGA-BG-A0LX-01A | 5.779180485784229 | Tumor | UCEC |
| TCGA-EY-A5W2-01A | 5.2501884911815555 | Tumor | UCEC |
| TCGA-AX-A2HA-11A | 0.7570080193298515 | Normal | UCEC |
| TCGA-D1-A2G0-01A | 6.451198418582785 | Tumor | UCEC |
| TCGA-AP-A053-01A | 6.345381136241161 | Tumor | UCEC |
| TCGA-A5-A0G1-01A | 4.554106083306367 | Tumor | UCEC |
| TCGA-EO-A3KW-01A | 5.693118123545152 | Tumor | UCEC |
| TCGA-BG-A0MI-01A | 5.0529090890345865 | Tumor | UCEC |
| TCGA-AJ-A3NG-01A | 5.060026987134869 | Tumor | UCEC |
| TCGA-AJ-A3OJ-01A | 4.081891780606865 | Tumor | UCEC |
| TCGA-A5-A2K5-01A | 5.231601524940063 | Tumor | UCEC |
| TCGA-AX-A1C7-01A | 4.834360783148951 | Tumor | UCEC |
| TCGA-D1-A167-01A | 4.969412585935775 | Tumor | UCEC |
| TCGA-B5-A11X-01A | 6.663932560379365 | Tumor | UCEC |
| TCGA-BS-A0V4-01A | 3.7782363334469555 | Tumor | UCEC |
| TCGA-DF-A2L0-01A | 6.309040755255084 | Tumor | UCEC |
| TCGA-BK-A6W3-01A | 4.6313553164405254 | Tumor | UCEC |
| TCGA-FI-A2EU-01A | 5.70531074465119 | Tumor | UCEC |
| TCGA-AX-A3FS-01A | 5.898685714681808 | Tumor | UCEC |
| TCGA-D1-A17R-01A | 5.124281117909046 | Tumor | UCEC |
| TCGA-AJ-A5DW-01A | 4.835535503671481 | Tumor | UCEC |
| TCGA-EY-A1GH-01A | 5.359859595389406 | Tumor | UCEC |
| TCGA-AJ-A2QL-11A | 0.4614246899394935 | Normal | UCEC |
| TCGA-AP-A1DM-01A | 5.541484192539559 | Tumor | UCEC |
| TCGA-BG-A0MS-01A | 5.091662003541068 | Tumor | UCEC |
| TCGA-E6-A1LZ-01A | 6.162691762169931 | Tumor | UCEC |
| TCGA-B5-A11E-01A | 6.500632306289541 | Tumor | UCEC |
| TCGA-FI-A2F9-01A | 4.295769997161849 | Tumor | UCEC |
| TCGA-BG-A0YV-01A | 5.645453452110432 | Tumor | UCEC |
| TCGA-AP-A1DR-01A | 5.687027845719128 | Tumor | UCEC |
| TCGA-AP-A0LG-01A | 5.923414828566797 | Tumor | UCEC |
| TCGA-BG-A186-01A | 3.010233661345462 | Tumor | UCEC |
| TCGA-AX-A1CK-01A | 4.832812827024329 | Tumor | UCEC |
| TCGA-AX-A2HC-01A | 5.679466357686692 | Tumor | UCEC |
| TCGA-BG-A18C-01A | 6.829671446460819 | Tumor | UCEC |
| TCGA-EY-A2OQ-01A | 4.45346162705866 | Tumor | UCEC |
| TCGA-DF-A2KR-01A | 2.7330445347584003 | Tumor | UCEC |
| TCGA-FI-A3PV-01A | 5.9174240993161344 | Tumor | UCEC |
| TCGA-BK-A0CA-01B | 2.6842377435408586 | Tumor | UCEC |
| TCGA-AJ-A23M-01A | 5.803530947391009 | Tumor | UCEC |
| TCGA-A5-A0R7-01A | 5.466539530766444 | Tumor | UCEC |
| TCGA-BG-A0W2-01A | 3.3079355908802968 | Tumor | UCEC |
| TCGA-PG-A5BC-01A | 5.792215635580309 | Tumor | UCEC |
| TCGA-5B-A90C-01A | 6.5876431189625135 | Tumor | UCEC |
| TCGA-A5-A0GU-01A | 3.5392226044685082 | Tumor | UCEC |
| TCGA-A5-A1OF-01A | 6.274668151166948 | Tumor | UCEC |
| TCGA-AX-A1CE-01A | 4.914753916452301 | Tumor | UCEC |
| TCGA-EY-A215-01A | 5.770426757036013 | Tumor | UCEC |
| TCGA-EY-A1GT-01A | 3.966749220995193 | Tumor | UCEC |
| TCGA-AP-A0LI-01A | 6.773902497948992 | Tumor | UCEC |
| TCGA-B5-A1MW-01A | 5.601976109954775 | Tumor | UCEC |
| TCGA-AX-A0IW-01A | 5.4092680426220605 | Tumor | UCEC |
| TCGA-EY-A2ON-01A | 5.249833487489048 | Tumor | UCEC |
| TCGA-B5-A11G-01A | 5.151481708066202 | Tumor | UCEC |
| TCGA-QS-A5YR-01A | 5.184801635348059 | Tumor | UCEC |
| TCGA-BS-A0U5-01A | 4.221093374925972 | Tumor | UCEC |
| TCGA-SL-A6J9-01A | 6.85210927782201 | Tumor | UCEC |
| TCGA-EO-A1Y7-01A | 5.95457241176989 | Tumor | UCEC |
| TCGA-BS-A0UJ-01A | 2.130696771436336 | Tumor | UCEC |
| TCGA-AX-A1C8-01A | 5.034984261626234 | Tumor | UCEC |
| TCGA-DF-A2KY-01A | 5.267430904659474 | Tumor | UCEC |
| TCGA-B5-A11L-01B | 4.359229133583501 | Tumor | UCEC |
| TCGA-EO-A1Y8-01A | 5.594209980228442 | Tumor | UCEC |
| TCGA-FI-A2F4-01A | 5.829995458418083 | Tumor | UCEC |
| TCGA-AJ-A2QL-01A | 3.925128856742727 | Tumor | UCEC |
| TCGA-BS-A0VI-01A | 3.368500189257717 | Tumor | UCEC |
| TCGA-FL-A1YL-11A | 0.28755737780140483 | Normal | UCEC |
| TCGA-AP-A0LF-01A | 5.528005183445732 | Tumor | UCEC |
| TCGA-EY-A549-01A | 2.888268276807337 | Tumor | UCEC |
| TCGA-B5-A11U-01A | 4.640364087446772 | Tumor | UCEC |
| TCGA-B5-A11W-01A | 4.423673554569215 | Tumor | UCEC |
| TCGA-B5-A0JS-01A | 3.483010713088466 | Tumor | UCEC |
| TCGA-AX-A06B-01A | 6.13555674491036 | Tumor | UCEC |
| TCGA-B5-A0JY-01A | 6.258970671172254 | Tumor | UCEC |
| TCGA-D1-A3JP-01A | 2.894793473067295 | Tumor | UCEC |
| TCGA-D1-A16V-01A | 5.619710000696811 | Tumor | UCEC |
| TCGA-DF-A2KV-01A | 5.213441201778766 | Tumor | UCEC |
| TCGA-EY-A1GS-01A | 5.750522025520246 | Tumor | UCEC |
| TCGA-AP-A1E0-01A | 7.014064646713164 | Tumor | UCEC |
| TCGA-BS-A0UT-01A | 3.412076152996831 | Tumor | UCEC |
| TCGA-A5-A0GI-01A | 5.308695916854673 | Tumor | UCEC |
| TCGA-BG-A0VX-01A | 4.643123397233087 | Tumor | UCEC |
| TCGA-A5-A1OG-01A | 5.98523129227866 | Tumor | UCEC |
| TCGA-D1-A0ZQ-01A | 4.849448646521369 | Tumor | UCEC |
| TCGA-D1-A174-01A | 5.069743732689814 | Tumor | UCEC |
| TCGA-EY-A1GK-01A | 4.498804416529615 | Tumor | UCEC |
| TCGA-EY-A1GX-01A | 4.158751763921026 | Tumor | UCEC |
| TCGA-FI-A2EW-01A | 6.047319452058769 | Tumor | UCEC |
| TCGA-D1-A17H-01A | 3.9950181273583225 | Tumor | UCEC |
| TCGA-FL-A3WE-11A | 2.5859158502734743 | Normal | UCEC |
| TCGA-B5-A0K1-01A | 4.834869494742092 | Tumor | UCEC |
| TCGA-B5-A11N-01A | 2.6190309394955333 | Tumor | UCEC |
| TCGA-AP-A0LJ-01A | 3.480598069683219 | Tumor | UCEC |
| TCGA-A5-A0GE-01A | 5.058647824476029 | Tumor | UCEC |
| TCGA-D1-A2G7-01A | 4.626304213373928 | Tumor | UCEC |
| TCGA-AJ-A3NE-11A | 0.62655284156681 | Normal | UCEC |
| TCGA-B5-A121-01A | 3.9668156180599126 | Tumor | UCEC |
| TCGA-BG-A0M0-01A | 4.877504334587418 | Tumor | UCEC |
| TCGA-A5-A0R9-01A | 4.139015609703861 | Tumor | UCEC |
| TCGA-BK-A6W4-01A | 3.9311778012314296 | Tumor | UCEC |
| TCGA-A5-A0GH-01A | 6.004979375491937 | Tumor | UCEC |
| TCGA-A5-A2K2-01A | 6.365474512244684 | Tumor | UCEC |
| TCGA-AX-A0IZ-11A | 0.12010865110701198 | Normal | UCEC |
| TCGA-BK-A13B-01A | 4.075418083778823 | Tumor | UCEC |
| TCGA-AP-A05N-01A | 4.813621279376418 | Tumor | UCEC |
| TCGA-A5-A0G5-01A | 5.170149557093425 | Tumor | UCEC |
| TCGA-BK-A139-01A | 5.111570742066586 | Tumor | UCEC |
| TCGA-AX-A3FV-01A | 7.841841863561468 | Tumor | UCEC |
| TCGA-PG-A6IB-01A | 6.720637202862469 | Tumor | UCEC |
| TCGA-AJ-A3BD-01A | 6.811069631499964 | Tumor | UCEC |
| TCGA-D1-A16J-01A | 7.326901227652537 | Tumor | UCEC |
| TCGA-EO-A3AS-01A | 3.5690577898282325 | Tumor | UCEC |
| TCGA-EY-A1GE-01A | 1.4597192169476665 | Tumor | UCEC |
| TCGA-A5-A0R6-01A | 5.6725659413047 | Tumor | UCEC |
| TCGA-BG-A3PP-11A | 0.15982789700884822 | Normal | UCEC |
| TCGA-EO-A3AU-01A | 5.060141602820414 | Tumor | UCEC |
| TCGA-EC-A1NJ-01A | 4.384776831418071 | Tumor | UCEC |
| TCGA-AX-A0J0-01A | 6.085109430962174 | Tumor | UCEC |
| TCGA-AJ-A3QS-01A | 5.60259351862974 | Tumor | UCEC |
| TCGA-D1-A17D-01A | 4.333986169460759 | Tumor | UCEC |
| TCGA-B5-A3FC-01A | 4.750431323674289 | Tumor | UCEC |
| TCGA-FI-A2D2-01A | 4.137970964048729 | Tumor | UCEC |
| TCGA-A5-A0GX-01A | 3.037515615276147 | Tumor | UCEC |
| TCGA-BG-A0M7-01A | 4.956911433613363 | Tumor | UCEC |
| TCGA-BG-A0VW-01A | 4.476559187744328 | Tumor | UCEC |
| TCGA-AX-A2HF-01A | 5.8039976663961275 | Tumor | UCEC |
| TCGA-AX-A0IU-01A | 5.789538797705764 | Tumor | UCEC |
| TCGA-A5-A0R8-01A | 4.547549698725392 | Tumor | UCEC |
| TCGA-DI-A2QU-01A | 6.086209743158805 | Tumor | UCEC |
| TCGA-D1-A15W-01A | 3.5341418030764387 | Tumor | UCEC |
| TCGA-B5-A0JN-01A | 5.931177014476578 | Tumor | UCEC |
| TCGA-B5-A11O-01A | 5.012381675016519 | Tumor | UCEC |
| TCGA-EY-A548-01A | 2.7952602312575645 | Tumor | UCEC |
| TCGA-B5-A11S-01A | 4.126385106489623 | Tumor | UCEC |
| TCGA-AX-A1CP-01A | 4.555691541275446 | Tumor | UCEC |
| TCGA-BK-A0CC-01A | 5.387636311659347 | Tumor | UCEC |
| TCGA-DI-A1BU-01A | 5.111162171763861 | Tumor | UCEC |
| TCGA-AP-A1DP-01A | 3.776925328534151 | Tumor | UCEC |
| TCGA-AX-A06F-01A | 5.625246885580071 | Tumor | UCEC |
| TCGA-B5-A3FH-01A | 3.567281442397436 | Tumor | UCEC |
| TCGA-QF-A5YT-01A | 5.274023736168173 | Tumor | UCEC |
| TCGA-B5-A1N2-01A | 6.116815732748005 | Tumor | UCEC |
| TCGA-BK-A0CB-11A | 0.31675614527864293 | Normal | UCEC |
| TCGA-EY-A1GD-01A | 4.509800756019142 | Tumor | UCEC |
| TCGA-AX-A2H8-01A | 5.064357206213714 | Tumor | UCEC |
| TCGA-BG-A0VT-01A | 3.511871701979076 | Tumor | UCEC |
| TCGA-AX-A2H7-01A | 5.021595381161981 | Tumor | UCEC |
| TCGA-A5-A0VP-01A | 4.13131342896461 | Tumor | UCEC |
| TCGA-AJ-A3NC-11A | 0.4029840312817441 | Normal | UCEC |
| TCGA-D1-A17Q-01A | 4.958123900573305 | Tumor | UCEC |
| TCGA-DI-A2QY-01A | 5.145558128520111 | Tumor | UCEC |
| TCGA-AP-A1DO-01A | 5.6725628869045615 | Tumor | UCEC |
| TCGA-BG-A0LW-01A | 2.1488639757548547 | Tumor | UCEC |
| TCGA-D1-A161-01A | 4.620999211486642 | Tumor | UCEC |
| TCGA-DF-A2KN-01A | 5.477011416691561 | Tumor | UCEC |
| TCGA-B5-A1MV-01A | 5.172101358421921 | Tumor | UCEC |
| TCGA-D1-A2G5-01A | 4.915939264818603 | Tumor | UCEC |
| TCGA-D1-A165-01A | 5.245060709796931 | Tumor | UCEC |
| TCGA-EY-A212-01A | 6.631032314008052 | Tumor | UCEC |
| TCGA-AJ-A5DV-01A | 4.757316654208983 | Tumor | UCEC |
| TCGA-EY-A1GP-01A | 5.03627893828527 | Tumor | UCEC |
| TCGA-BG-A0W1-01A | 3.8901497004601957 | Tumor | UCEC |
| TCGA-BG-A2AD-01A | 5.495894636671535 | Tumor | UCEC |
| TCGA-FL-A1YF-11A | 1.469280784260043 | Normal | UCEC |
| TCGA-BG-A3PP-01A | 5.5852983286531614 | Tumor | UCEC |
| TCGA-AP-A0LT-01A | 6.320264726291937 | Tumor | UCEC |
| TCGA-BG-A0RY-01A | 3.47090890006581 | Tumor | UCEC |
| TCGA-BS-A0TE-01A | 6.826368099780727 | Tumor | UCEC |
| TCGA-BS-A0V7-01A | 2.4771995627248913 | Tumor | UCEC |
| TCGA-BK-A56F-01A | 3.8012159260173584 | Tumor | UCEC |
| TCGA-BK-A26L-01A | 4.538581361273226 | Tumor | UCEC |
| TCGA-SL-A6JA-01A | 4.234317408862191 | Tumor | UCEC |
| TCGA-D1-A17A-01A | 2.193702357623218 | Tumor | UCEC |
| TCGA-BK-A4ZD-01A | 4.921819625513988 | Tumor | UCEC |
| TCGA-BK-A139-01C | 3.0775213194058852 | Tumor | UCEC |
| TCGA-AX-A05Y-01A | 5.769155122507196 | Tumor | UCEC |
| TCGA-A5-A2K7-01A | 6.3712337368030125 | Tumor | UCEC |
| TCGA-D1-A0ZP-01A | 9.289428357808498 | Tumor | UCEC |
| TCGA-AJ-A3BF-01A | 5.665761877078679 | Tumor | UCEC |
| TCGA-BS-A0UF-01A | 6.082602965618343 | Tumor | UCEC |
| TCGA-BS-A0U9-01B | 6.8722030106915915 | Tumor | UCEC |
| TCGA-AP-A05A-01A | 5.979026647059122 | Tumor | UCEC |
| TCGA-EO-A3B0-01A | 6.9437760933212465 | Tumor | UCEC |
| TCGA-D1-A175-01A | 5.134911119679656 | Tumor | UCEC |
| TCGA-AX-A3G4-01A | 6.175744815578277 | Tumor | UCEC |
| TCGA-BS-A0TA-01A | 5.186792686596349 | Tumor | UCEC |
| TCGA-B5-A0K3-01A | 4.977546808721613 | Tumor | UCEC |
| TCGA-B5-A3S1-01A | 4.527633707867554 | Tumor | UCEC |
| TCGA-FL-A1YT-11A | 0.4503483354313955 | Normal | UCEC |
| TCGA-KJ-A3U4-01A | 5.181159833755761 | Tumor | UCEC |
| TCGA-EO-A3B1-01A | 5.939110879390804 | Tumor | UCEC |
| TCGA-EY-A2OO-01A | 3.64048647008812 | Tumor | UCEC |
| TCGA-AJ-A3I9-01A | 6.032697729054919 | Tumor | UCEC |
| TCGA-B5-A0KB-01B | 6.996684923007871 | Tumor | UCEC |
| TCGA-AP-A1DK-01A | 4.666379924655544 | Tumor | UCEC |
| TCGA-D1-A177-01A | 6.691685965872241 | Tumor | UCEC |
| TCGA-BG-A0M3-01A | 4.627558054276208 | Tumor | UCEC |
| TCGA-BS-A0UV-01A | 6.2040385939557465 | Tumor | UCEC |
| TCGA-D1-A16Y-01A | 5.406096404447958 | Tumor | UCEC |
| TCGA-AJ-A3BI-01A | 7.122861731180739 | Tumor | UCEC |
| TCGA-EY-A1GF-01A | 3.7738289811841605 | Tumor | UCEC |
| TCGA-D1-A17U-01A | 4.589415508323863 | Tumor | UCEC |
| TCGA-E6-A1M0-11A | 0.1448293306460232 | Normal | UCEC |
| TCGA-D1-A0ZZ-01A | 3.7058865664115386 | Tumor | UCEC |
| TCGA-FL-A1YQ-11A | 3.049157376763436 | Normal | UCEC |
| TCGA-A5-A0GD-01A | 5.655880123357808 | Tumor | UCEC |
| TCGA-AP-A05J-01A | 5.623377614020609 | Tumor | UCEC |
| TCGA-4E-A92E-01A | 3.5909051425937326 | Tumor | UCEC |
| TCGA-BG-A18A-01A | 4.237319569889612 | Tumor | UCEC |
| TCGA-FL-A1YG-11A | 0.5773905270921622 | Normal | UCEC |
| TCGA-KP-A3VZ-01A | 6.007066129944703 | Tumor | UCEC |
| TCGA-EC-A24G-01A | 3.744739225101081 | Tumor | UCEC |
| TCGA-AX-A1C4-01A | 6.120596813809792 | Tumor | UCEC |
| TCGA-BG-A0VV-01A | 4.689841440657594 | Tumor | UCEC |
| TCGA-EY-A1G7-01A | 6.145707378452804 | Tumor | UCEC |
| TCGA-AJ-A8CT-01A | 2.4370469828767076 | Tumor | UCEC |
| TCGA-BG-A0M4-01A | 3.383379351570628 | Tumor | UCEC |
| TCGA-BG-A0VZ-01A | 4.610686948029203 | Tumor | UCEC |
| TCGA-QS-A744-01A | 5.163318300915831 | Tumor | UCEC |
| TCGA-FI-A2D6-01A | 6.411860146845487 | Tumor | UCEC |
| TCGA-A5-A0G3-01A | 5.154669812264218 | Tumor | UCEC |
| TCGA-AX-A3G9-01A | 4.492888917869139 | Tumor | UCEC |
| TCGA-QS-A8F1-01A | 5.730113201002775 | Tumor | UCEC |
| TCGA-BK-A0C9-01A | 5.614054231393145 | Tumor | UCEC |
| TCGA-D1-A1O8-01A | 5.991842848648201 | Tumor | UCEC |
| TCGA-BS-A0U8-01A | 4.5058632218388865 | Tumor | UCEC |
| TCGA-EO-A22R-01A | 5.432665014010009 | Tumor | UCEC |
| TCGA-B5-A11Q-01A | 4.617049386422223 | Tumor | UCEC |
| TCGA-B5-A3FD-01A | 5.997532258052597 | Tumor | UCEC |
| TCGA-AJ-A3NE-01A | 4.806390887617397 | Tumor | UCEC |
| TCGA-EY-A1GM-01A | 5.4220050383779625 | Tumor | UCEC |
| TCGA-EY-A547-01A | 8.286046022474912 | Tumor | UCEC |
| TCGA-AX-A3G7-01A | 5.000753149751996 | Tumor | UCEC |
| TCGA-D1-A16X-01A | 4.977503811238377 | Tumor | UCEC |
| TCGA-DI-A0WH-01A | 4.5081712558823535 | Tumor | UCEC |
| TCGA-EY-A2OM-01A | 3.9336606426841016 | Tumor | UCEC |
| TCGA-AJ-A3NH-11A | 0.7994589524223871 | Normal | UCEC |
| TCGA-AJ-A2QM-01A | 5.3081574664976285 | Tumor | UCEC |
| TCGA-BG-A0MA-01A | 5.039942508744498 | Tumor | UCEC |
| TCGA-AP-A0LH-01A | 5.8635628490283205 | Tumor | UCEC |
| TCGA-AJ-A2QO-01A | 5.121272028649869 | Tumor | UCEC |
| TCGA-D1-A17T-01A | 4.344755332945702 | Tumor | UCEC |
| TCGA-AP-A056-01A | 5.491616190431807 | Tumor | UCEC |
| TCGA-BS-A0U7-01A | 4.291759940665831 | Tumor | UCEC |
| TCGA-A5-AB3J-01A | 2.9867526613425417 | Tumor | UCEC |
| TCGA-B5-A0JX-01A | 5.224170335762481 | Tumor | UCEC |
| TCGA-FL-A1YH-11A | 1.987744573867636 | Normal | UCEC |
| TCGA-QS-A5YQ-01A | 4.057486990211695 | Tumor | UCEC |
| TCGA-AX-A062-01A | 4.845681386105872 | Tumor | UCEC |
| TCGA-BG-A0MC-01A | 3.6751922713274214 | Tumor | UCEC |
| TCGA-D1-A0ZN-01A | 4.003082363439695 | Tumor | UCEC |
| TCGA-EY-A4KR-01A | 5.903220299716528 | Tumor | UCEC |
| TCGA-A5-A7WK-01A | 5.349812438163693 | Tumor | UCEC |
| TCGA-EY-A3L3-01A | 4.825851966256527 | Tumor | UCEC |
| TCGA-AX-A2HC-11A | 0.7165102961291336 | Normal | UCEC |
| TCGA-AJ-A3BH-01A | 3.9158178943290634 | Tumor | UCEC |
| TCGA-AJ-A3IA-01A | 6.425962381022484 | Tumor | UCEC |
| TCGA-AX-A1CK-11A | 1.7362614702826782 | Normal | UCEC |
| TCGA-AX-A0J0-11A | 0.5859088776150263 | Normal | UCEC |
| TCGA-EO-A22S-01A | 6.955030457106713 | Tumor | UCEC |
| TCGA-B5-A0JU-01B | 5.019502255862611 | Tumor | UCEC |
| TCGA-D1-A16B-01A | 4.148820638011761 | Tumor | UCEC |
| TCGA-AX-A3FX-01A | 5.971942779951139 | Tumor | UCEC |
| TCGA-AX-A1CF-01A | 6.273457015881501 | Tumor | UCEC |
| TCGA-A5-A0GA-01A | 5.729341446140209 | Tumor | UCEC |
| TCGA-SJ-A6ZJ-01A | 4.655167215600747 | Tumor | UCEC |
| TCGA-AX-A3FT-01A | 3.9260047503011455 | Tumor | UCEC |
| TCGA-AP-A0LD-01A | 6.549517686167075 | Tumor | UCEC |
| TCGA-AJ-A3OL-01A | 4.854743372291178 | Tumor | UCEC |
| TCGA-AP-A052-01A | 6.32923596231978 | Tumor | UCEC |
| TCGA-D1-A15X-01A | 5.956312774055054 | Tumor | UCEC |
| TCGA-EY-A1GW-01A | 3.7174476731712867 | Tumor | UCEC |
| TCGA-B5-A3FB-01A | 4.610060062787625 | Tumor | UCEC |
| TCGA-EY-A72D-01A | 5.627146506401718 | Tumor | UCEC |
| TCGA-AX-A05Y-11A | 0.44830645533575764 | Normal | UCEC |
| TCGA-B5-A11V-01A | 4.635858910039352 | Tumor | UCEC |
| TCGA-EY-A2OP-01A | 5.4789286944161 | Tumor | UCEC |
| TCGA-BS-A0TD-01A | 4.623688042026459 | Tumor | UCEC |
| TCGA-B5-A11J-01A | 4.699680639298176 | Tumor | UCEC |
| TCGA-BG-A0MK-01A | 2.979632992895063 | Tumor | UCEC |
| TCGA-D1-A17B-01A | 3.9180317567399823 | Tumor | UCEC |
| TCGA-AX-A3G3-01A | 6.3855076467460545 | Tumor | UCEC |
| TCGA-FI-A2D0-01A | 4.810209344594041 | Tumor | UCEC |
| TCGA-BG-A0YU-01A | 3.409232861016104 | Tumor | UCEC |
| TCGA-AJ-A3EK-01A | 5.959372472959832 | Tumor | UCEC |
| TCGA-B5-A11F-01A | 4.421833104198438 | Tumor | UCEC |
| TCGA-AX-A06L-01A | 4.446742428694024 | Tumor | UCEC |
| TCGA-EO-A3AY-01A | 6.157915129689073 | Tumor | UCEC |
| TCGA-A5-A0GG-01A | 5.87485106796183 | Tumor | UCEC |
| TCGA-A5-A0GN-01A | 3.3720482852121254 | Tumor | UCEC |
| TCGA-BG-A220-01A | 4.76847564708519 | Tumor | UCEC |
| TCGA-D1-A1NY-01A | 5.443991332930552 | Tumor | UCEC |
| TCGA-AP-A059-01A | 5.657897943049259 | Tumor | UCEC |
| TCGA-FI-A2F8-01A | 6.489419135004825 | Tumor | UCEC |
| TCGA-AX-A06H-01A | 4.9437061391775945 | Tumor | UCEC |
| TCGA-AP-A0L9-01A | 6.41685212226345 | Tumor | UCEC |
| TCGA-A5-A7WJ-01A | 5.697388853400282 | Tumor | UCEC |
| TCGA-AX-A1CI-01A | 3.6112997216817875 | Tumor | UCEC |
| TCGA-D1-A1NU-01A | 6.510431809358551 | Tumor | UCEC |
| TCGA-H5-A2HR-01A | 6.085195830586679 | Tumor | UCEC |
| TCGA-AX-A1CR-01A | 5.34300056231853 | Tumor | UCEC |
| TCGA-DI-A1NO-01A | 5.637751786849561 | Tumor | UCEC |
| TCGA-E6-A8L9-01A | 4.8119356883708955 | Tumor | UCEC |
| TCGA-A5-A0G9-01A | 4.319872013230424 | Tumor | UCEC |
| TCGA-DF-A2KZ-01A | 3.511968626322961 | Tumor | UCEC |
| TCGA-DI-A2QU-11A | 0.3497669091952671 | Normal | UCEC |
| TCGA-BG-A2L7-01A | 3.9652245977604217 | Tumor | UCEC |
| TCGA-B5-A1MZ-01A | 2.8711816977486238 | Tumor | UCEC |
| TCGA-BK-A13C-11A | 0.14922720767170344 | Normal | UCEC |
| TCGA-B5-A1MR-01A | 6.024418130806772 | Tumor | UCEC |
| TCGA-D1-A176-01A | 4.864996129314606 | Tumor | UCEC |
| TCGA-B5-A1MU-01A | 5.426962906454536 | Tumor | UCEC |
| TCGA-AX-A3G6-01A | 6.949154812231023 | Tumor | UCEC |
| TCGA-BG-A0MG-01A | 5.567508938044291 | Tumor | UCEC |
| TCGA-E6-A1LX-01A | 6.876877134863843 | Tumor | UCEC |
| TCGA-BS-A0WQ-01A | 2.3469588750675254 | Tumor | UCEC |
| TCGA-A5-A1OK-01A | 3.5280916544296077 | Tumor | UCEC |
| TCGA-D1-A0ZR-01A | 4.136749270201953 | Tumor | UCEC |
| TCGA-K6-A3WQ-01A | 3.9566609021988057 | Tumor | UCEC |
| TCGA-D1-A17L-01A | 2.8496696568928983 | Tumor | UCEC |
| TCGA-D1-A3JQ-01A | 4.5005750885632745 | Tumor | UCEC |
| TCGA-D1-A15V-01A | 5.6815359955322355 | Tumor | UCEC |
| TCGA-D1-A1NX-01A | 5.964751646407868 | Tumor | UCEC |
| TCGA-PG-A7D5-01A | 5.136572227390324 | Tumor | UCEC |
| TCGA-AX-A063-01A | 4.67777923877843 | Tumor | UCEC |
| TCGA-EY-A54A-01A | 5.060736014649866 | Tumor | UCEC |
| TCGA-B5-A1MX-01A | 5.771249353881684 | Tumor | UCEC |
| TCGA-FI-A2EX-01A | 7.177071034256368 | Tumor | UCEC |
| TCGA-AP-A1E4-01A | 4.750396910680175 | Tumor | UCEC |
| TCGA-A5-A2K3-01A | 6.234058266583875 | Tumor | UCEC |
| TCGA-AJ-A3NF-01A | 4.715571618640064 | Tumor | UCEC |
| TCGA-BK-A0CA-01A | 4.598943049094232 | Tumor | UCEC |
| TCGA-BS-A0UA-01A | 4.020712145026674 | Tumor | UCEC |
| TCGA-D1-A1O0-01A | 7.610915006103475 | Tumor | UCEC |
| TCGA-EO-A2CG-01A | 5.720460530008293 | Tumor | UCEC |
| TCGA-EY-A1GU-01A | 3.3914297459713563 | Tumor | UCEC |
| TCGA-DI-A2QY-11A | 0.5560931841771662 | Normal | UCEC |
| TCGA-DF-A2KS-01A | 6.138013123068519 | Tumor | UCEC |
| TCGA-AJ-A3BG-01A | 4.266719587201881 | Tumor | UCEC |
| TCGA-A5-A0VQ-01A | 3.78433673035149 | Tumor | UCEC |
| TCGA-B5-A3FA-01A | 4.7739834848414295 | Tumor | UCEC |
| TCGA-B5-A11H-01A | 5.550257214229612 | Tumor | UCEC |
| TCGA-AP-A1E3-01A | 5.0727090030412185 | Tumor | UCEC |
| TCGA-D1-A17C-01A | 3.69639925620962 | Tumor | UCEC |
| TCGA-B5-A0K8-01A | 4.484751546202784 | Tumor | UCEC |
| TCGA-A5-A0GJ-01A | 4.522539038235077 | Tumor | UCEC |
| TCGA-AX-A2HK-01A | 6.024561030570912 | Tumor | UCEC |
| TCGA-AP-A1DH-01A | 6.140678749926521 | Tumor | UCEC |
| TCGA-AX-A1CA-01A | 5.7072398136963916 | Tumor | UCEC |
| TCGA-BG-A3EW-11A | 0.3658585095354744 | Normal | UCEC |
| TCGA-PG-A917-01A | 3.6552989757482113 | Tumor | UCEC |
| TCGA-BK-A0CB-01A | 3.618365176200109 | Tumor | UCEC |
| TCGA-BG-A221-01A | 5.962112639172216 | Tumor | UCEC |
| TCGA-KP-A3W3-01A | 6.072204725323046 | Tumor | UCEC |
| TCGA-BG-A0MH-01A | 5.596427885052497 | Tumor | UCEC |
| TCGA-FI-A2D5-01A | 6.198016983730845 | Tumor | UCEC |
| TCGA-AX-A1CC-01A | 5.072434596525576 | Tumor | UCEC |
| TCGA-B5-A1MS-01B | 4.94641510743241 | Tumor | UCEC |
| TCGA-BG-A187-01A | 4.700477184489196 | Tumor | UCEC |
| TCGA-D1-A17N-01A | 3.8053076302269657 | Tumor | UCEC |
| TCGA-B5-A11Y-01A | 3.4663919161955743 | Tumor | UCEC |
| TCGA-AX-A05W-01A | 4.566174000266505 | Tumor | UCEC |
| TCGA-EY-A1H0-01A | 5.374273700851642 | Tumor | UCEC |
| TCGA-D1-A1O5-01A | 3.5493515491953525 | Tumor | UCEC |
| TCGA-D1-A1NS-01A | 3.937799169711524 | Tumor | UCEC |
| TCGA-D1-A16I-01A | 6.8189014579096074 | Tumor | UCEC |
| TCGA-AX-A3FZ-01A | 5.611203653378517 | Tumor | UCEC |
| TCGA-AX-A3GI-01A | 6.220730712421179 | Tumor | UCEC |
| TCGA-D1-A179-01A | 5.863691144374988 | Tumor | UCEC |
| TCGA-EY-A1G8-01A | 6.430334199273486 | Tumor | UCEC |
| TCGA-EY-A1GI-01A | 5.118126353452652 | Tumor | UCEC |
| TCGA-A5-A0G2-01A | 6.116004370562579 | Tumor | UCEC |
| TCGA-D1-A101-01A | 4.872275823069744 | Tumor | UCEC |
| TCGA-D1-A17S-01A | 3.1783799796504253 | Tumor | UCEC |
| TCGA-AJ-A3NC-01A | 3.2496180918673008 | Tumor | UCEC |
| TCGA-E6-A2P8-01A | 5.853141575330566 | Tumor | UCEC |
| TCGA-AX-A3FW-01A | 4.75319009984717 | Tumor | UCEC |
| TCGA-KP-A3W0-01A | 3.7114814696232514 | Tumor | UCEC |
| TCGA-B5-A11M-01A | 2.8506899583222487 | Tumor | UCEC |
| TCGA-E6-A1M0-01A | 5.339826278135996 | Tumor | UCEC |
| TCGA-AX-A2HA-01A | 5.169148807117339 | Tumor | UCEC |
| TCGA-AX-A2IN-01A | 5.376443948821272 | Tumor | UCEC |
| TCGA-D1-A0ZO-01A | 3.8751736361820552 | Tumor | UCEC |
| TCGA-D1-A16S-01A | 5.4957653012379755 | Tumor | UCEC |
| TCGA-AP-A051-01A | 5.602128331185694 | Tumor | UCEC |
| TCGA-D1-A2G6-01A | 3.5316050898994873 | Tumor | UCEC |
| TCGA-AJ-A3OK-01A | 1.4113707521700294 | Tumor | UCEC |
| TCGA-D1-A17K-01A | 6.869754950602528 | Tumor | UCEC |
| TCGA-D1-A3DH-01A | 6.484680012636348 | Tumor | UCEC |
| TCGA-BG-A0MO-01A | 2.8661934929801514 | Tumor | UCEC |
| TCGA-D1-A1O7-01A | 5.504316918261653 | Tumor | UCEC |
| TCGA-B5-A0JV-01A | 5.247702084603202 | Tumor | UCEC |
| TCGA-AX-A064-01A | 5.054227352966628 | Tumor | UCEC |
| TCGA-AX-A1CN-01A | 5.3755507082249965 | Tumor | UCEC |
| TCGA-B5-A0K4-01A | 4.108612650897772 | Tumor | UCEC |
| TCGA-FI-A2EY-01A | 4.515311519311143 | Tumor | UCEC |
| TCGA-D1-A0ZV-01A | 3.6679454411345276 | Tumor | UCEC |
| TCGA-AX-A06D-01A | 3.6792792136593686 | Tumor | UCEC |
| TCGA-AP-A05P-01A | 3.111009849821033 | Tumor | UCEC |
| TCGA-EY-A214-01A | 5.751833629091029 | Tumor | UCEC |
| TCGA-A5-A0GW-01A | 6.22181341166701 | Tumor | UCEC |
| TCGA-EY-A3QX-01A | 4.955641503551032 | Tumor | UCEC |
| TCGA-EO-A3L0-01A | 5.390302219141926 | Tumor | UCEC |
| TCGA-B5-A5OC-01A | 3.1982236060694023 | Tumor | UCEC |
| TCGA-EO-A2CH-01A | 4.723703566802243 | Tumor | UCEC |
| TCGA-AX-A1CI-11A | 0.410078363575587 | Normal | UCEC |
| TCGA-A5-A0GP-01A | 6.228005054368606 | Tumor | UCEC |
| TCGA-EO-A22Y-01A | 4.468752124625018 | Tumor | UCEC |
| TCGA-AX-A05Z-01A | 5.695204952626378 | Tumor | UCEC |
| TCGA-B5-A11I-01A | 5.638559740257953 | Tumor | UCEC |
| TCGA-EO-A22U-01A | 5.17412109181513 | Tumor | UCEC |
| TCGA-BS-A0TC-01A | 3.743780966281165 | Tumor | UCEC |
| TCGA-B5-A0K9-01A | 5.353222626720749 | Tumor | UCEC |
| TCGA-AX-A3G1-01A | 6.272389309032486 | Tumor | UCEC |
| TCGA-D1-A16G-01A | 5.230846770472643 | Tumor | UCEC |
| TCGA-AP-A0LN-01A | 3.23638223789008 | Tumor | UCEC |
| TCGA-EO-A22X-01A | 5.355918377035958 | Tumor | UCEC |
| TCGA-JU-AAVI-01A | 5.444010039578613 | Tumor | UCEC |
| TCGA-AJ-A3NH-01A | 5.664563905960175 | Tumor | UCEC |
| TCGA-A5-A3LO-01A | 5.955089864029792 | Tumor | UCEC |
| TCGA-BG-A3EW-01A | 4.917273823412779 | Tumor | UCEC |
| TCGA-D1-A3DG-01A | 6.548471125287307 | Tumor | UCEC |
| TCGA-AX-A2H8-11A | 0.2726939471159881 | Normal | UCEC |
| TCGA-AX-A2H4-01A | 5.168608849942274 | Tumor | UCEC |
| TCGA-AJ-A3TW-01A | 6.083910081072841 | Tumor | UCEC |
| TCGA-AP-A3K1-01A | 5.736908461122901 | Tumor | UCEC |
| TCGA-AX-A3GB-01A | 1.5598051804654671 | Tumor | UCEC |
| TCGA-FI-A3PX-01A | 6.207400696621774 | Tumor | UCEC |
| TCGA-QF-A5YS-01A | 3.595574878851177 | Tumor | UCEC |
| TCGA-D1-A168-01A | 4.395067507577853 | Tumor | UCEC |
| TCGA-FI-A2CX-01A | 4.263648676249075 | Tumor | UCEC |
| TCGA-AX-A2H5-01A | 3.7156541606520563 | Tumor | UCEC |
| TCGA-FL-A1YI-11A | 0.37939074848019455 | Normal | UCEC |
| TCGA-AX-A1C9-01A | 5.5250889047346945 | Tumor | UCEC |
| TCGA-DI-A2QT-01A | 5.258500130210889 | Tumor | UCEC |
| TCGA-AX-A3G8-01A | 5.654168357051804 | Tumor | UCEC |
| TCGA-BK-A0CC-01B | 3.6453886585771498 | Tumor | UCEC |
| TCGA-AW-A1PO-01A | 5.396824161422306 | Tumor | UCEC |
| TCGA-EO-A3AV-01A | 6.61678659241634 | Tumor | UCEC |
| TCGA-AX-A2HG-01A | 5.554374636644299 | Tumor | UCEC |
| TCGA-KP-A3W4-01A | 4.358116874784622 | Tumor | UCEC |
| TCGA-A5-A0GQ-01A | 4.131146570799156 | Tumor | UCEC |
| TCGA-A5-A1OH-01A | 5.633018077189109 | Tumor | UCEC |
| TCGA-D1-A16F-01A | 4.089531365238929 | Tumor | UCEC |
| TCGA-B5-A11R-01A | 4.346389365872306 | Tumor | UCEC |
| TCGA-D1-A17F-01A | 3.9722089326283307 | Tumor | UCEC |
| TCGA-BK-A4ZD-11A | 1.6648746840904711 | Normal | UCEC |
| TCGA-AX-A2IO-01A | 6.08266688001699 | Tumor | UCEC |
| TCGA-EC-A1QX-01A | 5.382804236864985 | Tumor | UCEC |
| TCGA-AP-A054-01A | 6.558615630867537 | Tumor | UCEC |
| TCGA-BG-A0M6-01A | 5.374105633770746 | Tumor | UCEC |
| TCGA-2E-A9G8-01A | 5.9348902099906145 | Tumor | UCEC |
| TCGA-B5-A5OE-01A | 5.958543225905702 | Tumor | UCEC |
| TCGA-B5-A0JT-01A | 5.442660722121031 | Tumor | UCEC |
| TCGA-AP-A0LP-01A | 4.340762523587311 | Tumor | UCEC |
| TCGA-BG-A2AD-11A | 0.3658977522546664 | Normal | UCEC |
| TCGA-DI-A1BY-01A | 4.490939610679613 | Tumor | UCEC |
| TCGA-B5-A1MY-01A | 5.425296746563663 | Tumor | UCEC |
| TCGA-FL-A1YM-11A | 0.4451826903083902 | Normal | UCEC |
| TCGA-EY-A1GQ-01A | 5.575579593135943 | Tumor | UCEC |
| TCGA-BG-A0MU-01A | 3.7593069653548294 | Tumor | UCEC |
| TCGA-SJ-A6ZI-01A | 4.595133422769413 | Tumor | UCEC |
| TCGA-AX-A0J1-01A | 5.930761007153032 | Tumor | UCEC |
| TCGA-BS-A0V8-01A | 4.807165676484816 | Tumor | UCEC |
| TCGA-AP-A0LM-01A | 4.864744297421235 | Tumor | UCEC |
| TCGA-AX-A1CF-11A | 0.8987622356883649 | Normal | UCEC |
| TCGA-BS-A0TG-01A | 3.733309669419423 | Tumor | UCEC |
| TCGA-BG-A0MQ-01A | 4.529426784427109 | Tumor | UCEC |
| TCGA-AP-A0LV-01A | 5.214930045220936 | Tumor | UCEC |
| TCGA-EY-A1GO-01A | 5.839218493053455 | Tumor | UCEC |
| TCGA-B5-A0JZ-01A | 5.411284027822725 | Tumor | UCEC |
| TCGA-AX-A060-01A | 5.24032976691245 | Tumor | UCEC |
| TCGA-AX-A2HH-01A | 4.806093743087877 | Tumor | UCEC |
| TCGA-EO-A3AZ-01A | 4.960534755130202 | Tumor | UCEC |
| TCGA-DF-A2KU-01A | 6.483784798750249 | Tumor | UCEC |
| TCGA-FL-A1YN-11A | 0.5987008880197275 | Normal | UCEC |
| TCGA-B5-A0K6-01A | 4.231468510587118 | Tumor | UCEC |
| TCGA-AP-A0LL-01A | 2.70834263710736 | Tumor | UCEC |
| TCGA-A5-A2K4-01A | 5.488485961384437 | Tumor | UCEC |
| TCGA-D1-A163-01A | 5.871193677188537 | Tumor | UCEC |
| TCGA-D1-A17M-01A | 5.566126717005188 | Tumor | UCEC |
| TCGA-B5-A0K0-01A | 3.3208482385902425 | Tumor | UCEC |
| TCGA-BG-A0M9-01A | 4.24045798941697 | Tumor | UCEC |
| TCGA-BG-A0MT-01A | 4.692049795231723 | Tumor | UCEC |
| TCGA-B5-A0K7-01A | 3.601889574235346 | Tumor | UCEC |
| TCGA-A5-A0VO-01A | 5.035969480790467 | Tumor | UCEC |
| TCGA-AJ-A3BK-01A | 5.762056704930013 | Tumor | UCEC |
| TCGA-D1-A102-01A | 4.420892356062547 | Tumor | UCEC |
| TCGA-AJ-A8CV-01A | 5.2445967237613935 | Tumor | UCEC |
| TCGA-FI-A2D4-01A | 6.2263452519836004 | Tumor | UCEC |
| TCGA-AP-A0LO-01A | 5.131098944762248 | Tumor | UCEC |
| TCGA-A5-A0GR-01A | 3.581335321439093 | Tumor | UCEC |
| TCGA-AX-A0IS-01A | 4.195072527118497 | Tumor | UCEC |
| TCGA-E6-A2P9-01A | 4.826729548377341 | Tumor | UCEC |
| TCGA-BS-A0TJ-01A | 5.943304017912802 | Tumor | UCEC |
| TCGA-AJ-A2QK-01A | 5.593711522305273 | Tumor | UCEC |
| TCGA-AP-A0LS-01A | 5.33562737014391 | Tumor | UCEC |
| TCGA-BK-A139-02A | 5.096654061989738 | Tumor | UCEC |
| TCGA-AP-A0LE-01A | 6.15880829953109 | Tumor | UCEC |
| TCGA-AX-A2HJ-01A | 5.488777700726351 | Tumor | UCEC |
| TCGA-AJ-A3EM-01A | 8.70817425624642 | Tumor | UCEC |
| TCGA-EO-A22T-01A | 5.976431073435796 | Tumor | UCEC |
| TCGA-A5-A0RA-01A | 4.8686819591486294 | Tumor | UCEC |
| TCGA-AJ-A3EL-01A | 5.9929428091806285 | Tumor | UCEC |
| TCGA-D1-A16O-01A | 3.278933272513397 | Tumor | UCEC |
| TCGA-A5-A0GB-01A | 6.584447876275704 | Tumor | UCEC |
| TCGA-D1-A16Q-01A | 4.713124009682662 | Tumor | UCEC |
| TCGA-BG-A222-01A | 4.782083812980246 | Tumor | UCEC |
| TCGA-PG-A916-01A | 3.801874967828084 | Tumor | UCEC |
| TCGA-AX-A1C5-01A | 5.147684128470534 | Tumor | UCEC |
| TCGA-FL-A1YU-11A | 4.4345883923853435 | Normal | UCEC |
| TCGA-PG-A915-01A | 5.133720745284552 | Tumor | UCEC |
| TCGA-AX-A2HD-01A | 5.6367124711269865 | Tumor | UCEC |
| TCGA-BG-A18B-01A | 4.382059610273269 | Tumor | UCEC |
| TCGA-AP-A1E1-01A | 4.924547335747093 | Tumor | UCEC |
| TCGA-A5-A3LP-01A | 4.596699692686904 | Tumor | UCEC |
| TCGA-AX-A2HD-11A | 1.5116750947902973 | Normal | UCEC |
| TCGA-AP-A1DV-01A | 4.363924381262482 | Tumor | UCEC |
| TCGA-BS-A0TI-01A | 5.539028604940113 | Tumor | UCEC |
| TCGA-AX-A2H2-01A | 5.431395044313396 | Tumor | UCEC |
| TCGA-AX-A05S-01A | 4.171987252503479 | Tumor | UCEC |
| TCGA-D1-A162-01A | 5.3703679238775015 | Tumor | UCEC |
| TCGA-D1-A0ZS-01A | 5.670847570900889 | Tumor | UCEC |
| TCGA-BS-A0V6-01A | 4.604441711283595 | Tumor | UCEC |
| TCGA-KP-A3W1-01A | 5.198394923551479 | Tumor | UCEC |
| TCGA-D1-A0ZU-01A | 4.505145891805457 | Tumor | UCEC |
| TCGA-FL-A1YV-11A | 0.35407952726112873 | Normal | UCEC |
| TCGA-D1-A3DA-01A | 3.1924343451873236 | Tumor | UCEC |
| TCGA-AP-A05H-01A | 4.714063713418395 | Tumor | UCEC |
| TCGA-D1-A16N-01A | 6.70179182258894 | Tumor | UCEC |
| TCGA-AP-A05D-01A | 5.644322411052481 | Tumor | UCEC |
| TCGA-BG-A2AE-01A | 3.442645566000639 | Tumor | UCEC |
| TCGA-AX-A1CJ-01A | 4.164945872948269 | Tumor | UCEC |
| TCGA-AP-A5FX-01A | 5.370088084996355 | Tumor | UCEC |
| TCGA-EY-A210-01A | 4.808566464365554 | Tumor | UCEC |
| TCGA-D1-A169-01A | 5.45376068511411 | Tumor | UCEC |
| TCGA-PG-A914-01A | 5.281160941995794 | Tumor | UCEC |
| TCGA-BG-A0M2-01A | 4.1200545746613 | Tumor | UCEC |
| TCGA-D1-A1NW-01A | 4.551619955102899 | Tumor | UCEC |
| TCGA-AJ-A8CW-01A | 5.130425846916371 | Tumor | UCEC |
| TCGA-EY-A1GC-01A | 5.028573064812241 | Tumor | UCEC |
| TCGA-A5-A0GV-01A | 5.1814925654065656 | Tumor | UCEC |
| TCGA-BS-A0UM-01A | 4.369061252165651 | Tumor | UCEC |
| TCGA-B5-A5OD-01A | 4.283333079867412 | Tumor | UCEC |
| TCGA-A5-A0GM-01A | 5.074879964981985 | Tumor | UCEC |
| TCGA-FI-A2CY-01A | 5.939897037596602 | Tumor | UCEC |
| TCGA-EY-A1GR-01A | 6.2989405495767015 | Tumor | UCEC |
| TCGA-B5-A0JR-01A | 5.544171580383524 | Tumor | UCEC |
| TCGA-5S-A9Q8-01A | 3.5641983552768908 | Tumor | UCEC |
| TCGA-AP-A1DQ-01A | 4.812166139213178 | Tumor | UCEC |
| TCGA-AX-A05U-01A | 4.1528511683379365 | Tumor | UCEC |
| TCGA-BS-A0UL-01A | 4.808751229001594 | Tumor | UCEC |
| TCGA-D1-A16E-01A | 4.416662291593371 | Tumor | UCEC |
| TCGA-AX-A0IZ-01A | 4.756047414579396 | Tumor | UCEC |
| TCGA-DI-A1NN-01A | 5.729938885079121 | Tumor | UCEC |
| TCGA-AJ-A23O-01A | 5.390594429882494 | Tumor | UCEC |
| TCGA-AP-A0L8-01A | 4.656110818477177 | Tumor | UCEC |
| TCGA-BK-A26L-01C | 3.261163338656639 | Tumor | UCEC |
| TCGA-BS-A0T9-01A | 2.7172166940714977 | Tumor | UCEC |
| TCGA-A5-A1OJ-01A | 1.677334576832913 | Tumor | UCEC |
| TCGA-BK-A13C-01A | 2.785179538506946 | Tumor | UCEC |
| TCGA-EO-A3KX-01A | 5.349732947441031 | Tumor | UCEC |
| TCGA-D1-A15Z-01A | 3.8690470650372424 | Tumor | UCEC |
| TCGA-D1-A16D-01A | 2.994451164029137 | Tumor | UCEC |
| TCGA-EO-A1Y5-01A | 6.6910750726735095 | Tumor | UCEC |
| TCGA-AJ-A23N-01A | 5.821341690418635 | Tumor | UCEC |
| TCGA-AJ-A3EJ-01A | 5.8191881490729696 | Tumor | UCEC |
| TCGA-EY-A1GL-01A | 1.4962329311980174 | Tumor | UCEC |
| TCGA-B5-A3F9-01A | 4.498522475013723 | Tumor | UCEC |
| TCGA-D1-A16R-01A | 4.541095604674097 | Tumor | UCEC |
| TCGA-B5-A11Z-01A | 2.7211348040086625 | Tumor | UCEC |
| TCGA-AP-A05O-01A | 5.316086960771418 | Tumor | UCEC |
| TCGA-D1-A1NZ-01A | 4.820534143312035 | Tumor | UCEC |
| TCGA-EY-A1GV-01A | 4.602831071320558 | Tumor | UCEC |
| TCGA-D1-A103-01A | 4.692265370862227 | Tumor | UCEC |
| TCGA-EO-A3KU-01A | 5.211917815585626 | Tumor | UCEC |
| TCGA-B5-A0K2-01A | 3.628489164808318 | Tumor | UCEC |
| TCGA-AJ-A2QN-01A | 5.450625302793069 | Tumor | UCEC |
| TCGA-D1-A160-01A | 5.19395774373021 | Tumor | UCEC |

**Supplementary Table 7**: Raw data of patient survival durations from the TCGA

| **id** | **futime** | **fustat** | **MYBL2** | **Type** | **CancerType** |
| --- | --- | --- | --- | --- | --- |
| TCGA-AJ-A3NH-01A | 1 | 1 | 5.66456390596017 | Tumor | UCEC |
| TCGA-SL-A6J9-01A | 2 | 0 | 6.85210927782201 | Tumor | UCEC |
| TCGA-AJ-A8CW-01A | 4 | 0 | 5.13042584691637 | Tumor | UCEC |
| TCGA-AX-A3FZ-01A | 6 | 0 | 5.61120365337852 | Tumor | UCEC |
| TCGA-AX-A3FT-01A | 6 | 0 | 3.92600475030115 | Tumor | UCEC |
| TCGA-SL-A6JA-01A | 7 | 0 | 4.23431740886219 | Tumor | UCEC |
| TCGA-AX-A3FS-01A | 7 | 0 | 5.89868571468181 | Tumor | UCEC |
| TCGA-AX-A3G6-01A | 8 | 0 | 6.94915481223102 | Tumor | UCEC |
| TCGA-AX-A3G4-01A | 9 | 0 | 6.17574481557828 | Tumor | UCEC |
| TCGA-AX-A3G7-01A | 9 | 0 | 5.00075315 | Tumor | UCEC |
| TCGA-AX-A3GI-01A | 12 | 0 | 6.22073071242118 | Tumor | UCEC |
| TCGA-4E-A92E-01A | 13 | 0 | 3.59090514259373 | Tumor | UCEC |
| TCGA-AX-A3FW-01A | 17 | 0 | 4.75319009984717 | Tumor | UCEC |
| TCGA-D1-A0ZR-01A | 17 | 0 | 4.13674927020195 | Tumor | UCEC |
| TCGA-AW-A1PO-01A | 17 | 0 | 5.39682416142231 | Tumor | UCEC |
| TCGA-D1-A165-01A | 20 | 0 | 5.24506070979693 | Tumor | UCEC |
| TCGA-D1-A16R-01A | 21 | 0 | 4.5410956046741 | Tumor | UCEC |
| TCGA-D1-A1NW-01A | 22 | 0 | 4.5516199551029 | Tumor | UCEC |
| TCGA-D1-A17C-01A | 30 | 0 | 3.69639925620962 | Tumor | UCEC |
| TCGA-D1-A1O7-01A | 32 | 0 | 5.50431691826165 | Tumor | UCEC |
| TCGA-AX-A3G8-01A | 36 | 0 | 5.6541683570518 | Tumor | UCEC |
| TCGA-D1-A17F-01A | 39 | 0 | 3.97220893262833 | Tumor | UCEC |
| TCGA-AX-A3G3-01A | 42 | 0 | 6.38550764674605 | Tumor | UCEC |
| TCGA-D1-A1NS-01A | 43 | 0 | 3.93779916971152 | Tumor | UCEC |
| TCGA-D1-A17N-01A | 46 | 0 | 3.80530763022697 | Tumor | UCEC |
| TCGA-D1-A17A-01A | 48 | 0 | 2.19370235762322 | Tumor | UCEC |
| TCGA-AJ-A3EJ-01A | 50 | 1 | 5.81918814907297 | Tumor | UCEC |
| TCGA-H5-A2HR-01A | 53 | 0 | 6.08519583058668 | Tumor | UCEC |
| TCGA-D1-A101-01A | 53 | 0 | 4.87227582306974 | Tumor | UCEC |
| TCGA-D1-A17Q-01A | 54 | 0 | 4.95812390057331 | Tumor | UCEC |
| TCGA-BG-A221-01A | 55 | 0 | 5.96211263917222 | Tumor | UCEC |
| TCGA-D1-A15Z-01A | 58 | 1 | 3.86904706503724 | Tumor | UCEC |
| TCGA-D1-A1O0-01A | 63 | 0 | 7.61091500610348 | Tumor | UCEC |
| TCGA-DF-A2KN-01A | 67 | 0 | 5.47701141669156 | Tumor | UCEC |
| TCGA-D1-A17T-01A | 71 | 0 | 4.3447553329457 | Tumor | UCEC |
| TCGA-E6-A2P8-01A | 72 | 1 | 5.85314157533057 | Tumor | UCEC |
| TCGA-D1-A16F-01A | 80 | 0 | 4.08953136523893 | Tumor | UCEC |
| TCGA-AJ-A8CT-01A | 82 | 0 | 2.43704698287671 | Tumor | UCEC |
| TCGA-AX-A2HK-01A | 90 | 1 | 6.02456103057091 | Tumor | UCEC |
| TCGA-D1-A16X-01A | 111 | 0 | 4.97750381123838 | Tumor | UCEC |
| TCGA-D1-A160-01A | 112 | 0 | 5.19395774373021 | Tumor | UCEC |
| TCGA-D1-A16V-01A | 120 | 1 | 5.61971000069681 | Tumor | UCEC |
| TCGA-D1-A1O8-01A | 120 | 1 | 5.9918428486482 | Tumor | UCEC |
| TCGA-BG-A0W1-01A | 122 | 0 | 3.8901497004602 | Tumor | UCEC |
| TCGA-D1-A3DG-01A | 130 | 1 | 6.54847112528731 | Tumor | UCEC |
| TCGA-D1-A102-01A | 135 | 0 | 4.42089235606255 | Tumor | UCEC |
| TCGA-QS-A8F1-01A | 135 | 1 | 5.73011320100277 | Tumor | UCEC |
| TCGA-AX-A2H2-01A | 145 | 1 | 5.4313950443134 | Tumor | UCEC |
| TCGA-BS-A0TE-01A | 146 | 1 | 6.82636809978073 | Tumor | UCEC |
| TCGA-D1-A0ZS-01A | 148 | 0 | 5.67084757090089 | Tumor | UCEC |
| TCGA-EY-A72D-01A | 179 | 0 | 5.62714650640172 | Tumor | UCEC |
| TCGA-EY-A1G7-01A | 189 | 1 | 6.1457073784528 | Tumor | UCEC |
| TCGA-D1-A175-01A | 202 | 0 | 5.13491111967966 | Tumor | UCEC |
| TCGA-AP-A1DV-01A | 204 | 0 | 4.36392438126248 | Tumor | UCEC |
| TCGA-PG-A917-01A | 212 | 0 | 3.65529897574821 | Tumor | UCEC |
| TCGA-D1-A17B-01A | 213 | 0 | 3.91803175673998 | Tumor | UCEC |
| TCGA-EC-A1QX-01A | 224 | 0 | 5.38280423686498 | Tumor | UCEC |
| TCGA-FI-A2D4-01A | 243 | 1 | 6.2263452519836 | Tumor | UCEC |
| TCGA-PG-A915-01A | 249 | 0 | 5.13372074528455 | Tumor | UCEC |
| TCGA-D1-A2G6-01A | 258 | 1 | 3.53160508989949 | Tumor | UCEC |
| TCGA-FI-A2EX-01A | 284 | 0 | 7.17707103425637 | Tumor | UCEC |
| TCGA-DI-A1NO-01A | 285 | 1 | 5.63775178684956 | Tumor | UCEC |
| TCGA-KP-A3W1-01A | 288 | 0 | 5.19839492355148 | Tumor | UCEC |
| TCGA-BG-A222-01A | 291 | 0 | 4.78208381298025 | Tumor | UCEC |
| TCGA-BK-A6W4-01A | 300 | 0 | 3.93117780123143 | Tumor | UCEC |
| TCGA-BK-A6W3-01A | 305 | 0 | 4.63135531644053 | Tumor | UCEC |
| TCGA-EY-A212-01A | 310 | 1 | 6.63103231400805 | Tumor | UCEC |
| TCGA-D1-A17M-01A | 311 | 0 | 5.56612671700519 | Tumor | UCEC |
| TCGA-AJ-A23O-01A | 312 | 1 | 5.39059442988249 | Tumor | UCEC |
| TCGA-5S-A9Q8-01A | 313 | 0 | 3.56419835527689 | Tumor | UCEC |
| TCGA-D1-A16Q-01A | 324 | 0 | 4.71312400968266 | Tumor | UCEC |
| TCGA-BG-A0MA-01A | 326 | 1 | 5.0399425087445 | Tumor | UCEC |
| TCGA-EO-A1Y5-01A | 332 | 0 | 6.69107507267351 | Tumor | UCEC |
| TCGA-DF-A2KY-01A | 332 | 0 | 5.26743090465947 | Tumor | UCEC |
| TCGA-EY-A1GW-01A | 337 | 1 | 3.71744767317129 | Tumor | UCEC |
| TCGA-D1-A17R-01A | 339 | 1 | 5.12428111790905 | Tumor | UCEC |
| TCGA-A5-A7WJ-01A | 341 | 0 | 5.69738885340028 | Tumor | UCEC |
| TCGA-BG-A3EW-01A | 344 | 0 | 4.91727382341278 | Tumor | UCEC |
| TCGA-EY-A4KR-01A | 346 | 1 | 5.90322029971653 | Tumor | UCEC |
| TCGA-D1-A16I-01A | 350 | 1 | 6.81890145790961 | Tumor | UCEC |
| TCGA-B5-A1MY-01A | 361 | 1 | 5.42529674656366 | Tumor | UCEC |
| TCGA-AX-A06D-01A | 373 | 1 | 3.67927921365937 | Tumor | UCEC |
| TCGA-DI-A2QT-01A | 377 | 1 | 5.25850013021089 | Tumor | UCEC |
| TCGA-BG-A0YV-01A | 382 | 0 | 5.64545345211043 | Tumor | UCEC |
| TCGA-EY-A210-01A | 383 | 0 | 4.80856646436555 | Tumor | UCEC |
| TCGA-AP-A05O-01A | 384 | 0 | 5.31608696077142 | Tumor | UCEC |
| TCGA-AJ-A5DW-01A | 390 | 0 | 4.83553550367148 | Tumor | UCEC |
| TCGA-BK-A56F-01A | 401 | 0 | 3.80121592601736 | Tumor | UCEC |
| TCGA-D1-A1NU-01A | 402 | 0 | 6.51043180935855 | Tumor | UCEC |
| TCGA-KP-A3W0-01A | 404 | 0 | 3.71148146962325 | Tumor | UCEC |
| TCGA-AX-A1C4-01A | 404 | 0 | 6.12059681380979 | Tumor | UCEC |
| TCGA-B5-A0K9-01A | 409 | 1 | 5.35322262672075 | Tumor | UCEC |
| TCGA-AX-A05S-01A | 413 | 1 | 4.17198725250348 | Tumor | UCEC |
| TCGA-BG-A186-01A | 419 | 0 | 3.01023366134546 | Tumor | UCEC |
| TCGA-FI-A2D5-01A | 421 | 1 | 6.19801698373085 | Tumor | UCEC |
| TCGA-BG-A18A-01A | 426 | 0 | 4.23731956988961 | Tumor | UCEC |
| TCGA-K6-A3WQ-01A | 427 | 1 | 3.95666090219881 | Tumor | UCEC |
| TCGA-EY-A548-01A | 430 | 0 | 2.79526023125756 | Tumor | UCEC |
| TCGA-BG-A220-01A | 434 | 0 | 4.76847564708519 | Tumor | UCEC |
| TCGA-EY-A214-01A | 438 | 0 | 5.75183362909103 | Tumor | UCEC |
| TCGA-AJ-A23N-01A | 439 | 1 | 5.82134169041863 | Tumor | UCEC |
| TCGA-AX-A3GB-01A | 440 | 0 | 1.55980518046547 | Tumor | UCEC |
| TCGA-AJ-A3EK-01A | 446 | 0 | 5.95937247295983 | Tumor | UCEC |
| TCGA-B5-A1MW-01A | 447 | 0 | 5.60197610995477 | Tumor | UCEC |
| TCGA-EY-A54A-01A | 447 | 0 | 5.06073601464987 | Tumor | UCEC |
| TCGA-BG-A187-01A | 448 | 0 | 4.7004771844892 | Tumor | UCEC |
| TCGA-D1-A179-01A | 448 | 1 | 5.86369114437499 | Tumor | UCEC |
| TCGA-AJ-A3OL-01A | 449 | 1 | 4.85474337229118 | Tumor | UCEC |
| TCGA-QS-A744-01A | 451 | 0 | 5.16331830091583 | Tumor | UCEC |
| TCGA-BG-A0YU-01A | 451 | 0 | 3.4092328610161 | Tumor | UCEC |
| TCGA-A5-A3LP-01A | 455 | 0 | 4.5966996926869 | Tumor | UCEC |
| TCGA-EY-A1G8-01A | 456 | 1 | 6.43033419927349 | Tumor | UCEC |
| TCGA-D1-A1O5-01A | 458 | 0 | 3.54935154919535 | Tumor | UCEC |
| TCGA-AJ-A3OJ-01A | 467 | 0 | 4.08189178060687 | Tumor | UCEC |
| TCGA-BG-A0RY-01A | 469 | 0 | 3.47090890006581 | Tumor | UCEC |
| TCGA-BG-A18B-01A | 470 | 0 | 4.38205961027327 | Tumor | UCEC |
| TCGA-BG-A18C-01A | 470 | 0 | 6.82967144646082 | Tumor | UCEC |
| TCGA-AJ-A3NG-01A | 475 | 0 | 5.06002698713487 | Tumor | UCEC |
| TCGA-D1-A167-01A | 478 | 0 | 4.96941258593577 | Tumor | UCEC |
| TCGA-EY-A1GV-01A | 481 | 0 | 4.60283107132056 | Tumor | UCEC |
| TCGA-D1-A3DA-01A | 483 | 0 | 3.19243434518732 | Tumor | UCEC |
| TCGA-A5-A0VQ-01A | 485 | 0 | 3.78433673035149 | Tumor | UCEC |
| TCGA-AJ-A3OK-01A | 485 | 0 | 1.41137075217003 | Tumor | UCEC |
| TCGA-EC-A1NJ-01A | 488 | 0 | 4.38477683141807 | Tumor | UCEC |
| TCGA-EY-A1GT-01A | 490 | 0 | 3.96674922099519 | Tumor | UCEC |
| TCGA-DI-A0WH-01A | 496 | 0 | 4.50817125588235 | Tumor | UCEC |
| TCGA-EO-A3KW-01A | 497 | 0 | 5.69311812354515 | Tumor | UCEC |
| TCGA-B5-A121-01A | 504 | 0 | 3.96681561805991 | Tumor | UCEC |
| TCGA-BK-A4ZD-01A | 506 | 0 | 4.92181962551399 | Tumor | UCEC |
| TCGA-B5-A11U-01A | 509 | 0 | 4.64036408744677 | Tumor | UCEC |
| TCGA-EC-A24G-01A | 511 | 0 | 3.74473922510108 | Tumor | UCEC |
| TCGA-A5-AB3J-01A | 512 | 0 | 2.98675266134254 | Tumor | UCEC |
| TCGA-D1-A0ZN-01A | 512 | 0 | 4.0030823634397 | Tumor | UCEC |
| TCGA-AJ-A3TW-01A | 513 | 0 | 6.08391008107284 | Tumor | UCEC |
| TCGA-AJ-A3QS-01A | 513 | 0 | 5.60259351862974 | Tumor | UCEC |
| TCGA-D1-A1NY-01A | 513 | 0 | 5.44399133293055 | Tumor | UCEC |
| TCGA-D1-A3JP-01A | 515 | 0 | 2.8947934730673 | Tumor | UCEC |
| TCGA-EY-A5W2-01A | 516 | 0 | 5.25018849118156 | Tumor | UCEC |
| TCGA-AJ-A3I9-01A | 519 | 1 | 6.03269772905492 | Tumor | UCEC |
| TCGA-PG-A916-01A | 522 | 0 | 3.80187496782808 | Tumor | UCEC |
| TCGA-D1-A3JQ-01A | 526 | 0 | 4.50057508856327 | Tumor | UCEC |
| TCGA-B5-A11Z-01A | 528 | 0 | 2.72113480400866 | Tumor | UCEC |
| TCGA-D1-A16Y-01A | 532 | 0 | 5.40609640444796 | Tumor | UCEC |
| TCGA-A5-A0R7-01A | 535 | 0 | 5.46653953076644 | Tumor | UCEC |
| TCGA-EY-A1GR-01A | 536 | 0 | 6.2989405495767 | Tumor | UCEC |
| TCGA-JU-AAVI-01A | 540 | 1 | 5.44401003957861 | Tumor | UCEC |
| TCGA-AX-A2HH-01A | 540 | 0 | 4.80609374308788 | Tumor | UCEC |
| TCGA-A5-A0GA-01A | 543 | 1 | 5.72934144614021 | Tumor | UCEC |
| TCGA-EY-A1GQ-01A | 544 | 0 | 5.57557959313594 | Tumor | UCEC |
| TCGA-EY-A547-01A | 545 | 0 | 8.28604602247491 | Tumor | UCEC |
| TCGA-D1-A1NZ-01A | 548 | 0 | 4.82053414331203 | Tumor | UCEC |
| TCGA-D1-A177-01A | 548 | 0 | 6.69168596587224 | Tumor | UCEC |
| TCGA-AJ-A3BG-01A | 550 | 0 | 4.26671958720188 | Tumor | UCEC |
| TCGA-AJ-A3BF-01A | 551 | 0 | 5.66576187707868 | Tumor | UCEC |
| TCGA-KP-A3W4-01A | 554 | 1 | 4.35811687478462 | Tumor | UCEC |
| TCGA-D1-A1NX-01A | 555 | 0 | 5.96475164640787 | Tumor | UCEC |
| TCGA-SJ-A6ZJ-01A | 556 | 0 | 4.65516721560075 | Tumor | UCEC |
| TCGA-AX-A3G9-01A | 558 | 0 | 4.49288891786914 | Tumor | UCEC |
| TCGA-B5-A11Y-01A | 560 | 0 | 3.46639191619557 | Tumor | UCEC |
| TCGA-A5-A7WK-01A | 560 | 0 | 5.34981243816369 | Tumor | UCEC |
| TCGA-BG-A0LW-01A | 566 | 0 | 2.14886397575485 | Tumor | UCEC |
| TCGA-D1-A16S-01A | 568 | 0 | 5.49576530123798 | Tumor | UCEC |
| TCGA-EY-A1GP-01A | 573 | 0 | 5.03627893828527 | Tumor | UCEC |
| TCGA-D1-A3DH-01A | 573 | 0 | 6.48468001263635 | Tumor | UCEC |
| TCGA-EY-A215-01A | 574 | 0 | 5.77042675703601 | Tumor | UCEC |
| TCGA-AJ-A3BI-01A | 575 | 0 | 7.12286173118074 | Tumor | UCEC |
| TCGA-EY-A1GO-01A | 586 | 0 | 5.83921849305345 | Tumor | UCEC |
| TCGA-BG-A0M0-01A | 588 | 0 | 4.87750433458742 | Tumor | UCEC |
| TCGA-EY-A1H0-01A | 588 | 0 | 5.37427370085164 | Tumor | UCEC |
| TCGA-EO-A2CH-01A | 590 | 1 | 4.72370356680224 | Tumor | UCEC |
| TCGA-B5-A11W-01A | 593 | 0 | 4.42367355456922 | Tumor | UCEC |
| TCGA-D1-A174-01A | 594 | 0 | 5.06974373268981 | Tumor | UCEC |
| TCGA-B5-A11V-01A | 595 | 0 | 4.63585891003935 | Tumor | UCEC |
| TCGA-B5-A11X-01A | 596 | 0 | 6.66393256037937 | Tumor | UCEC |
| TCGA-A5-A0R8-01A | 596 | 0 | 4.54754969872539 | Tumor | UCEC |
| TCGA-AJ-A2QL-01A | 602 | 0 | 3.92512885674273 | Tumor | UCEC |
| TCGA-D1-A0ZO-01A | 602 | 0 | 3.87517363618206 | Tumor | UCEC |
| TCGA-EY-A549-01A | 605 | 0 | 2.88826827680734 | Tumor | UCEC |
| TCGA-BG-A2AD-01A | 609 | 0 | 5.49589463667153 | Tumor | UCEC |
| TCGA-EY-A2ON-01A | 610 | 1 | 5.24983348748905 | Tumor | UCEC |
| TCGA-BG-A2L7-01A | 612 | 0 | 3.96522459776042 | Tumor | UCEC |
| TCGA-BG-A0LX-01A | 614 | 0 | 5.77918048578423 | Tumor | UCEC |
| TCGA-AJ-A23M-01A | 616 | 1 | 5.80353094739101 | Tumor | UCEC |
| TCGA-BG-A0MU-01A | 617 | 0 | 3.75930696535483 | Tumor | UCEC |
| TCGA-AX-A3G1-01A | 624 | 1 | 6.27238930903249 | Tumor | UCEC |
| TCGA-AJ-A3NE-01A | 628 | 0 | 4.8063908876174 | Tumor | UCEC |
| TCGA-SJ-A6ZI-01A | 631 | 0 | 4.59513342276941 | Tumor | UCEC |
| TCGA-AJ-A3IA-01A | 633 | 0 | 6.42596238102248 | Tumor | UCEC |
| TCGA-AP-A05P-01A | 636 | 0 | 3.11100984982103 | Tumor | UCEC |
| TCGA-BG-A0M2-01A | 637 | 0 | 4.1200545746613 | Tumor | UCEC |
| TCGA-AJ-A2QK-01A | 643 | 0 | 5.59371152230527 | Tumor | UCEC |
| TCGA-BG-A0MT-01A | 644 | 0 | 4.69204979523172 | Tumor | UCEC |
| TCGA-AJ-A3NF-01A | 645 | 0 | 4.71557161864006 | Tumor | UCEC |
| TCGA-PG-A6IB-01A | 648 | 0 | 6.72063720286247 | Tumor | UCEC |
| TCGA-AP-A3K1-01A | 666 | 1 | 5.7369084611229 | Tumor | UCEC |
| TCGA-AP-A052-01A | 669 | 1 | 6.32923596231978 | Tumor | UCEC |
| TCGA-D1-A103-01A | 669 | 0 | 4.69226537086223 | Tumor | UCEC |
| TCGA-EY-A1GS-01A | 670 | 1 | 5.75052202552025 | Tumor | UCEC |
| TCGA-BG-A0M6-01A | 671 | 1 | 5.37410563377075 | Tumor | UCEC |
| TCGA-EO-A3KU-01A | 677 | 0 | 5.21191781558563 | Tumor | UCEC |
| TCGA-AJ-A3EM-01A | 677 | 0 | 8.70817425624642 | Tumor | UCEC |
| TCGA-AJ-A5DV-01A | 679 | 0 | 4.75731665420898 | Tumor | UCEC |
| TCGA-BG-A3PP-01A | 681 | 0 | 5.58529832865316 | Tumor | UCEC |
| TCGA-AJ-A3EL-01A | 684 | 0 | 5.99294280918063 | Tumor | UCEC |
| TCGA-D1-A0ZZ-01A | 686 | 0 | 3.70588656641154 | Tumor | UCEC |
| TCGA-PG-A914-01A | 687 | 0 | 5.28116094199579 | Tumor | UCEC |
| TCGA-QF-A5YS-01A | 689 | 0 | 3.59557487885118 | Tumor | UCEC |
| TCGA-EY-A1GK-01A | 693 | 0 | 4.49880441652962 | Tumor | UCEC |
| TCGA-EY-A2OM-01A | 694 | 0 | 3.9336606426841 | Tumor | UCEC |
| TCGA-D1-A17U-01A | 697 | 0 | 4.58941550832386 | Tumor | UCEC |
| TCGA-AX-A1CR-01A | 701 | 0 | 5.34300056231853 | Tumor | UCEC |
| TCGA-A5-A0GW-01A | 706 | 0 | 6.22181341166701 | Tumor | UCEC |
| TCGA-PG-A5BC-01A | 707 | 0 | 5.79221563558031 | Tumor | UCEC |
| TCGA-AP-A054-01A | 709 | 1 | 6.55861563086754 | Tumor | UCEC |
| TCGA-EY-A1GH-01A | 710 | 0 | 5.35985959538941 | Tumor | UCEC |
| TCGA-EY-A1GI-01A | 710 | 0 | 5.11812635345265 | Tumor | UCEC |
| TCGA-E6-A1LX-01A | 711 | 0 | 6.87687713486384 | Tumor | UCEC |
| TCGA-BG-A0MI-01A | 714 | 1 | 5.05290908903459 | Tumor | UCEC |
| TCGA-QS-A5YR-01A | 719 | 0 | 5.18480163534806 | Tumor | UCEC |
| TCGA-BG-A2AE-01A | 721 | 0 | 3.44264556600064 | Tumor | UCEC |
| TCGA-KP-A3VZ-01A | 722 | 0 | 6.0070661299447 | Tumor | UCEC |
| TCGA-AP-A05N-01A | 726 | 1 | 4.81362127937642 | Tumor | UCEC |
| TCGA-D1-A0ZV-01A | 728 | 0 | 3.66794544113453 | Tumor | UCEC |
| TCGA-BK-A26L-01A | 734 | 0 | 4.53858136127323 | Tumor | UCEC |
| TCGA-BK-A26L-01C | 734 | 0 | 3.26116333865664 | Tumor | UCEC |
| TCGA-D1-A17S-01A | 739 | 0 | 3.17837997965043 | Tumor | UCEC |
| TCGA-BS-A0TA-01A | 740 | 1 | 5.18679268659635 | Tumor | UCEC |
| TCGA-EY-A1GE-01A | 741 | 0 | 1.45971921694767 | Tumor | UCEC |
| TCGA-A5-A0R9-01A | 749 | 0 | 4.13901560970386 | Tumor | UCEC |
| TCGA-A5-A2K5-01A | 752 | 0 | 5.23160152494006 | Tumor | UCEC |
| TCGA-QS-A5YQ-01A | 759 | 0 | 4.05748699021169 | Tumor | UCEC |
| TCGA-BK-A13C-01A | 760 | 0 | 2.78517953850695 | Tumor | UCEC |
| TCGA-AP-A0LV-01A | 763 | 0 | 5.21493004522094 | Tumor | UCEC |
| TCGA-FI-A2D6-01A | 767 | 1 | 6.41186014684549 | Tumor | UCEC |
| TCGA-AX-A2H7-01A | 772 | 1 | 5.02159538116198 | Tumor | UCEC |
| TCGA-A5-A3LO-01A | 776 | 0 | 5.95508986402979 | Tumor | UCEC |
| TCGA-AX-A1C8-01A | 776 | 0 | 5.03498426162623 | Tumor | UCEC |
| TCGA-DF-A2L0-01A | 788 | 1 | 6.30904075525508 | Tumor | UCEC |
| TCGA-AX-A1CC-01A | 788 | 0 | 5.07243459652558 | Tumor | UCEC |
| TCGA-KP-A3W3-01A | 794 | 0 | 6.07220472532305 | Tumor | UCEC |
| TCGA-D1-A17L-01A | 802 | 0 | 2.8496696568929 | Tumor | UCEC |
| TCGA-AX-A1C9-01A | 802 | 0 | 5.52508890473469 | Tumor | UCEC |
| TCGA-EO-A3B1-01A | 820 | 0 | 5.9391108793908 | Tumor | UCEC |
| TCGA-B5-A0KB-01B | 824 | 0 | 6.99668492300787 | Tumor | UCEC |
| TCGA-B5-A11P-01B | 825 | 0 | 4.49380510757886 | Tumor | UCEC |
| TCGA-AP-A0LM-01A | 825 | 0 | 4.86474429742124 | Tumor | UCEC |
| TCGA-EY-A1GF-01A | 826 | 0 | 3.77382898118416 | Tumor | UCEC |
| TCGA-DF-A2KZ-01A | 826 | 0 | 3.51196862632296 | Tumor | UCEC |
| TCGA-EO-A3AZ-01A | 826 | 0 | 4.9605347551302 | Tumor | UCEC |
| TCGA-B5-A0K3-01A | 830 | 1 | 4.97754680872161 | Tumor | UCEC |
| TCGA-EY-A3L3-01A | 832 | 0 | 4.82585196625653 | Tumor | UCEC |
| TCGA-AX-A06H-01A | 833 | 0 | 4.94370613917759 | Tumor | UCEC |
| TCGA-D1-A16G-01A | 834 | 1 | 5.23084677047264 | Tumor | UCEC |
| TCGA-B5-A3FH-01A | 841 | 0 | 3.56728144239744 | Tumor | UCEC |
| TCGA-B5-A0K8-01A | 847 | 0 | 4.48475154620278 | Tumor | UCEC |
| TCGA-AP-A1E3-01A | 847 | 0 | 5.07270900304122 | Tumor | UCEC |
| TCGA-AX-A2HF-01A | 848 | 0 | 5.80399766639613 | Tumor | UCEC |
| TCGA-D1-A17H-01A | 852 | 0 | 3.99501812735832 | Tumor | UCEC |
| TCGA-D1-A17K-01A | 854 | 0 | 6.86975495060253 | Tumor | UCEC |
| TCGA-A5-A0GU-01A | 860 | 0 | 3.53922260446851 | Tumor | UCEC |
| TCGA-A5-A2K4-01A | 871 | 1 | 5.48848596138444 | Tumor | UCEC |
| TCGA-D1-A0ZU-01A | 872 | 0 | 4.50514589180546 | Tumor | UCEC |
| TCGA-A5-A0VO-01A | 875 | 1 | 5.03596948079047 | Tumor | UCEC |
| TCGA-FI-A2EY-01A | 880 | 1 | 4.51531151931114 | Tumor | UCEC |
| TCGA-A5-A0RA-01A | 884 | 0 | 4.86868195914863 | Tumor | UCEC |
| TCGA-EY-A2OQ-01A | 885 | 0 | 4.45346162705866 | Tumor | UCEC |
| TCGA-D1-A161-01A | 892 | 0 | 4.62099921148664 | Tumor | UCEC |
| TCGA-AJ-A2QM-01A | 894 | 0 | 5.30815746649763 | Tumor | UCEC |
| TCGA-EO-A1Y8-01A | 901 | 0 | 5.59420998022844 | Tumor | UCEC |
| TCGA-B5-A3S1-01A | 902 | 0 | 4.52763370786755 | Tumor | UCEC |
| TCGA-AX-A05U-01A | 903 | 0 | 4.15285116833794 | Tumor | UCEC |
| TCGA-D1-A15X-01A | 909 | 0 | 5.95631277405505 | Tumor | UCEC |
| TCGA-D1-A16J-01A | 910 | 0 | 7.32690122765254 | Tumor | UCEC |
| TCGA-D1-A0ZP-01A | 911 | 0 | 9.2894283578085 | Tumor | UCEC |
| TCGA-AX-A06J-01A | 913 | 0 | 3.2158685732225 | Tumor | UCEC |
| TCGA-D1-A15W-01A | 915 | 0 | 3.53414180307644 | Tumor | UCEC |
| TCGA-AX-A2H4-01A | 916 | 1 | 5.16860884994227 | Tumor | UCEC |
| TCGA-A5-A1OK-01A | 917 | 0 | 3.52809165442961 | Tumor | UCEC |
| TCGA-D1-A176-01A | 917 | 0 | 4.86499612931461 | Tumor | UCEC |
| TCGA-D1-A17D-01A | 926 | 0 | 4.33398616946076 | Tumor | UCEC |
| TCGA-FI-A2F8-01A | 933 | 0 | 6.48941913500482 | Tumor | UCEC |
| TCGA-BS-A0U7-01A | 935 | 1 | 4.29175994066583 | Tumor | UCEC |
| TCGA-AX-A2HJ-01A | 935 | 0 | 5.48877770072635 | Tumor | UCEC |
| TCGA-BK-A139-01A | 943 | 1 | 5.11157074206659 | Tumor | UCEC |
| TCGA-BK-A139-01C | 943 | 1 | 3.07752131940589 | Tumor | UCEC |
| TCGA-BK-A139-02A | 943 | 1 | 5.09665406198974 | Tumor | UCEC |
| TCGA-EY-A2OP-01A | 944 | 0 | 5.4789286944161 | Tumor | UCEC |
| TCGA-D1-A16N-01A | 945 | 0 | 6.70179182258894 | Tumor | UCEC |
| TCGA-DI-A2QU-01A | 946 | 1 | 6.0862097431588 | Tumor | UCEC |
| TCGA-AX-A2IN-01A | 947 | 1 | 5.37644394882127 | Tumor | UCEC |
| TCGA-AP-A1DO-01A | 952 | 0 | 5.67256288690456 | Tumor | UCEC |
| TCGA-B5-A1N2-01A | 954 | 1 | 6.11681573274801 | Tumor | UCEC |
| TCGA-BK-A13B-01A | 966 | 0 | 4.07541808377882 | Tumor | UCEC |
| TCGA-A5-A1OG-01A | 973 | 1 | 5.98523129227866 | Tumor | UCEC |
| TCGA-A5-A1OF-01A | 973 | 0 | 6.27466815116695 | Tumor | UCEC |
| TCGA-AX-A1CA-01A | 974 | 0 | 5.70723981369639 | Tumor | UCEC |
| TCGA-B5-A11Q-01A | 976 | 0 | 4.61704938642222 | Tumor | UCEC |
| TCGA-EY-A3QX-01A | 989 | 1 | 4.95564150355103 | Tumor | UCEC |
| TCGA-D1-A163-01A | 991 | 0 | 5.87119367718854 | Tumor | UCEC |
| TCGA-EY-A1GM-01A | 995 | 1 | 5.42200503837796 | Tumor | UCEC |
| TCGA-EY-A1GU-01A | 997 | 1 | 3.39142974597136 | Tumor | UCEC |
| TCGA-A5-A0GB-01A | 997 | 0 | 6.5844478762757 | Tumor | UCEC |
| TCGA-AX-A1C5-01A | 1001 | 0 | 5.14768412847053 | Tumor | UCEC |
| TCGA-A5-A1OJ-01A | 1006 | 0 | 1.67733457683291 | Tumor | UCEC |
| TCGA-AX-A05T-01A | 1010 | 0 | 4.33143889736863 | Tumor | UCEC |
| TCGA-E6-A8L9-01A | 1013 | 0 | 4.8119356883709 | Tumor | UCEC |
| TCGA-EY-A1GL-01A | 1014 | 0 | 1.49623293119802 | Tumor | UCEC |
| TCGA-AP-A0LO-01A | 1016 | 1 | 5.13109894476225 | Tumor | UCEC |
| TCGA-D1-A16O-01A | 1023 | 0 | 3.2789332725134 | Tumor | UCEC |
| TCGA-AJ-A3BK-01A | 1031 | 0 | 5.76205670493001 | Tumor | UCEC |
| TCGA-AX-A1CE-01A | 1037 | 0 | 4.9147539164523 | Tumor | UCEC |
| TCGA-B5-A5OD-01A | 1038 | 0 | 4.28333307986741 | Tumor | UCEC |
| TCGA-AJ-A3BD-01A | 1039 | 0 | 6.81106963149996 | Tumor | UCEC |
| TCGA-BG-A0VX-01A | 1043 | 0 | 4.64312339723309 | Tumor | UCEC |
| TCGA-AX-A2HC-01A | 1044 | 0 | 5.67946635768669 | Tumor | UCEC |
| TCGA-AJ-A2QO-01A | 1045 | 1 | 5.12127202864987 | Tumor | UCEC |
| TCGA-EO-A3AS-01A | 1046 | 0 | 3.56905778982823 | Tumor | UCEC |
| TCGA-AX-A1C7-01A | 1054 | 0 | 4.83436078314895 | Tumor | UCEC |
| TCGA-A5-A2K7-01A | 1063 | 0 | 6.37123373680301 | Tumor | UCEC |
| TCGA-B5-A5OE-01A | 1063 | 0 | 5.9585432259057 | Tumor | UCEC |
| TCGA-BK-A0CA-01A | 1064 | 0 | 4.59894304909423 | Tumor | UCEC |
| TCGA-BK-A0CA-01B | 1064 | 0 | 2.68423774354086 | Tumor | UCEC |
| TCGA-BK-A0CC-01B | 1067 | 0 | 3.64538865857715 | Tumor | UCEC |
| TCGA-BK-A0CC-01A | 1067 | 0 | 5.38763631165935 | Tumor | UCEC |
| TCGA-AJ-A8CV-01A | 1068 | 0 | 5.24459672376139 | Tumor | UCEC |
| TCGA-EO-A3AV-01A | 1069 | 0 | 6.61678659241634 | Tumor | UCEC |
| TCGA-AJ-A2QN-01A | 1069 | 0 | 5.45062530279307 | Tumor | UCEC |
| TCGA-BG-A0M3-01A | 1071 | 0 | 4.62755805427621 | Tumor | UCEC |
| TCGA-AX-A060-01A | 1075 | 0 | 5.24032976691245 | Tumor | UCEC |
| TCGA-DI-A1NN-01A | 1076 | 0 | 5.72993888507912 | Tumor | UCEC |
| TCGA-AP-A1DP-01A | 1081 | 1 | 3.77692532853415 | Tumor | UCEC |
| TCGA-AX-A0IU-01A | 1087 | 0 | 5.78953879770576 | Tumor | UCEC |
| TCGA-BK-A0CB-01A | 1092 | 0 | 3.61836517620011 | Tumor | UCEC |
| TCGA-BK-A0C9-01A | 1092 | 0 | 5.61405423139314 | Tumor | UCEC |
| TCGA-KJ-A3U4-01A | 1095 | 0 | 5.18115983375576 | Tumor | UCEC |
| TCGA-D1-A168-01A | 1098 | 0 | 4.39506750757785 | Tumor | UCEC |
| TCGA-AP-A5FX-01A | 1099 | 1 | 5.37008808499635 | Tumor | UCEC |
| TCGA-A5-A1OH-01A | 1105 | 0 | 5.63301807718911 | Tumor | UCEC |
| TCGA-AP-A0LH-01A | 1106 | 1 | 5.86356284902832 | Tumor | UCEC |
| TCGA-D1-A16B-01A | 1106 | 0 | 4.14882063801176 | Tumor | UCEC |
| TCGA-AX-A1CP-01A | 1107 | 0 | 4.55569154127545 | Tumor | UCEC |
| TCGA-B5-A0K1-01A | 1110 | 0 | 4.83486949474209 | Tumor | UCEC |
| TCGA-DI-A1BY-01A | 1114 | 0 | 4.49093961067961 | Tumor | UCEC |
| TCGA-D1-A16E-01A | 1119 | 0 | 4.41666229159337 | Tumor | UCEC |
| TCGA-A5-A0GR-01A | 1120 | 0 | 3.58133532143909 | Tumor | UCEC |
| TCGA-AP-A05J-01A | 1122 | 0 | 5.62337761402061 | Tumor | UCEC |
| TCGA-AJ-A3NC-01A | 1125 | 0 | 3.2496180918673 | Tumor | UCEC |
| TCGA-A5-A0G5-01A | 1126 | 0 | 5.17014955709343 | Tumor | UCEC |
| TCGA-EY-A1GX-01A | 1127 | 1 | 4.15875176392103 | Tumor | UCEC |
| TCGA-D1-A169-01A | 1135 | 0 | 5.45376068511411 | Tumor | UCEC |
| TCGA-D1-A16D-01A | 1141 | 0 | 2.99445116402914 | Tumor | UCEC |
| TCGA-AX-A1CN-01A | 1143 | 0 | 5.375550708 | Tumor | UCEC |
| TCGA-D1-A162-01A | 1145 | 0 | 5.3703679238775 | Tumor | UCEC |
| TCGA-AX-A0IW-01A | 1149 | 0 | 5.40926804262206 | Tumor | UCEC |
| TCGA-A5-A0R6-01A | 1153 | 0 | 5.6725659413047 | Tumor | UCEC |
| TCGA-EO-A3AY-01A | 1158 | 0 | 6.15791512968907 | Tumor | UCEC |
| TCGA-AX-A2HG-01A | 1163 | 0 | 5.5543746366443 | Tumor | UCEC |
| TCGA-EO-A3KX-01A | 1170 | 0 | 5.34973294744103 | Tumor | UCEC |
| TCGA-PG-A7D5-01A | 1177 | 0 | 5.13657222739032 | Tumor | UCEC |
| TCGA-DI-A1BU-01A | 1195 | 0 | 5.11116217176386 | Tumor | UCEC |
| TCGA-B5-A3FD-01A | 1197 | 1 | 5.9975322580526 | Tumor | UCEC |
| TCGA-D1-A15V-01A | 1201 | 0 | 5.68153599553224 | Tumor | UCEC |
| TCGA-5B-A90C-01A | 1202 | 0 | 6.58764311896251 | Tumor | UCEC |
| TCGA-FI-A2D0-01A | 1213 | 0 | 4.81020934459404 | Tumor | UCEC |
| TCGA-QF-A5YT-01A | 1215 | 0 | 5.27402373616817 | Tumor | UCEC |
| TCGA-AX-A0J0-01A | 1220 | 0 | 6.08510943096217 | Tumor | UCEC |
| TCGA-FI-A3PV-01A | 1249 | 1 | 5.91742409931613 | Tumor | UCEC |
| TCGA-B5-A5OC-01A | 1249 | 0 | 3.1982236060694 | Tumor | UCEC |
| TCGA-2E-A9G8-01A | 1249 | 0 | 5.93489020999061 | Tumor | UCEC |
| TCGA-BG-A0MC-01A | 1263 | 0 | 3.67519227132742 | Tumor | UCEC |
| TCGA-A5-A0VP-01A | 1288 | 0 | 4.13131342896461 | Tumor | UCEC |
| TCGA-BG-A0MO-01A | 1309 | 0 | 2.86619349298015 | Tumor | UCEC |
| TCGA-BG-A0MK-01A | 1317 | 1 | 2.97963299289506 | Tumor | UCEC |
| TCGA-AX-A0IZ-01A | 1333 | 0 | 4.7560474145794 | Tumor | UCEC |
| TCGA-AX-A3FX-01A | 1333 | 1 | 5.97194277995114 | Tumor | UCEC |
| TCGA-B5-A11M-01A | 1362 | 0 | 2.85068995832225 | Tumor | UCEC |
| TCGA-AP-A05H-01A | 1385 | 0 | 4.71406371341839 | Tumor | UCEC |
| TCGA-E6-A2P9-01A | 1386 | 0 | 4.82672954837734 | Tumor | UCEC |
| TCGA-EY-A2OO-01A | 1392 | 0 | 3.64048647008812 | Tumor | UCEC |
| TCGA-AP-A1E1-01A | 1395 | 0 | 4.92454733574709 | Tumor | UCEC |
| TCGA-AX-A062-01A | 1399 | 0 | 4.84568138610587 | Tumor | UCEC |
| TCGA-EO-A22U-01A | 1407 | 0 | 5.17412109181513 | Tumor | UCEC |
| TCGA-EO-A3B0-01A | 1412 | 0 | 6.94377609332125 | Tumor | UCEC |
| TCGA-AX-A2HD-01A | 1414 | 0 | 5.63671247112699 | Tumor | UCEC |
| TCGA-A5-A0GP-01A | 1416 | 0 | 6.22800505436861 | Tumor | UCEC |
| TCGA-AP-A0LJ-01A | 1421 | 0 | 3.48059806968322 | Tumor | UCEC |
| TCGA-AP-A1DQ-01A | 1423 | 1 | 4.81216613921318 | Tumor | UCEC |
| TCGA-A5-A0G3-01A | 1428 | 0 | 5.15466981226422 | Tumor | UCEC |
| TCGA-BS-A0T9-01A | 1428 | 1 | 2.7172166940715 | Tumor | UCEC |
| TCGA-D1-A2G5-01A | 1455 | 0 | 4.9159392648186 | Tumor | UCEC |
| TCGA-AP-A059-01A | 1461 | 0 | 5.65789794304926 | Tumor | UCEC |
| TCGA-B5-A1MX-01A | 1473 | 0 | 5.77124935388168 | Tumor | UCEC |
| TCGA-BG-A0MG-01A | 1477 | 0 | 5.56750893804429 | Tumor | UCEC |
| TCGA-B5-A0K0-01A | 1478 | 0 | 3.32084823859024 | Tumor | UCEC |
| TCGA-AP-A0L8-01A | 1484 | 1 | 4.65611081847718 | Tumor | UCEC |
| TCGA-D1-A2G0-01A | 1484 | 0 | 6.45119841858279 | Tumor | UCEC |
| TCGA-EO-A1Y7-01A | 1495 | 0 | 5.95457241176989 | Tumor | UCEC |
| TCGA-AP-A1DR-01A | 1495 | 0 | 5.68702784571913 | Tumor | UCEC |
| TCGA-AP-A0LT-01A | 1497 | 0 | 6.32026472629194 | Tumor | UCEC |
| TCGA-AX-A064-01A | 1517 | 0 | 5.05422735296663 | Tumor | UCEC |
| TCGA-EO-A3AU-01A | 1543 | 0 | 5.06014160282041 | Tumor | UCEC |
| TCGA-B5-A1MU-01A | 1548 | 1 | 5.42696290645454 | Tumor | UCEC |
| TCGA-BG-A0VV-01A | 1553 | 0 | 4.68984144065759 | Tumor | UCEC |
| TCGA-EO-A3L0-01A | 1554 | 0 | 5.39030221914193 | Tumor | UCEC |
| TCGA-AP-A1E4-01A | 1555 | 0 | 4.75039691068018 | Tumor | UCEC |
| TCGA-EO-A2CG-01A | 1564 | 0 | 5.72046053000829 | Tumor | UCEC |
| TCGA-B5-A1MV-01A | 1565 | 0 | 5.17210135842192 | Tumor | UCEC |
| TCGA-EO-A22Y-01A | 1567 | 0 | 4.46875212462502 | Tumor | UCEC |
| TCGA-BG-A0VT-01A | 1568 | 0 | 3.51187170197908 | Tumor | UCEC |
| TCGA-BG-A0VW-01A | 1582 | 0 | 4.47655918774433 | Tumor | UCEC |
| TCGA-B5-A1MZ-01A | 1598 | 0 | 2.87118169774862 | Tumor | UCEC |
| TCGA-BG-A0VZ-01A | 1601 | 0 | 4.6106869480292 | Tumor | UCEC |
| TCGA-EY-A1GD-01A | 1639 | 0 | 4.50980075601914 | Tumor | UCEC |
| TCGA-A5-A0GV-01A | 1643 | 0 | 5.18149256540657 | Tumor | UCEC |
| TCGA-EY-A1GC-01A | 1647 | 0 | 5.02857306481224 | Tumor | UCEC |
| TCGA-B5-A11J-01A | 1656 | 0 | 4.69968063929818 | Tumor | UCEC |
| TCGA-B5-A0JT-01A | 1682 | 0 | 5.44266072212103 | Tumor | UCEC |
| TCGA-AP-A1DM-01A | 1700 | 0 | 5.54148419253956 | Tumor | UCEC |
| TCGA-BG-A0W2-01A | 1721 | 0 | 3.3079355908803 | Tumor | UCEC |
| TCGA-B5-A0JR-01A | 1731 | 0 | 5.54417158038352 | Tumor | UCEC |
| TCGA-AX-A06F-01A | 1748 | 0 | 5.62524688558007 | Tumor | UCEC |
| TCGA-EO-A22X-01A | 1752 | 0 | 5.35591837703596 | Tumor | UCEC |
| TCGA-B5-A0K4-01A | 1757 | 0 | 4.10861265089777 | Tumor | UCEC |
| TCGA-AP-A05D-01A | 1758 | 0 | 5.64432241105248 | Tumor | UCEC |
| TCGA-B5-A0JX-01A | 1787 | 0 | 5.22417033576248 | Tumor | UCEC |
| TCGA-D1-A2G7-01A | 1793 | 1 | 4.62630421337393 | Tumor | UCEC |
| TCGA-E6-A1M0-01A | 1793 | 0 | 5.339826278 | Tumor | UCEC |
| TCGA-FI-A2EW-01A | 1795 | 0 | 6.04731945205877 | Tumor | UCEC |
| TCGA-B5-A0JU-01B | 1802 | 0 | 5.01950225586261 | Tumor | UCEC |
| TCGA-FI-A2F9-01A | 1803 | 0 | 4.29576999716185 | Tumor | UCEC |
| TCGA-BS-A0V4-01A | 1810 | 0 | 3.77823633344696 | Tumor | UCEC |
| TCGA-BG-A0MQ-01A | 1817 | 0 | 4.52942678442711 | Tumor | UCEC |
| TCGA-B5-A11E-01A | 1820 | 0 | 6.50063230628954 | Tumor | UCEC |
| TCGA-AP-A1E0-01A | 1832 | 0 | 7.01406464671316 | Tumor | UCEC |
| TCGA-A5-A0GM-01A | 1841 | 0 | 5.07487996498199 | Tumor | UCEC |
| TCGA-A5-A0GN-01A | 1841 | 0 | 3.37204828521213 | Tumor | UCEC |
| TCGA-AX-A06L-01A | 1845 | 0 | 4.44674242869402 | Tumor | UCEC |
| TCGA-AX-A0IS-01A | 1858 | 1 | 4.1950725271185 | Tumor | UCEC |
| TCGA-AX-A2H8-01A | 1861 | 0 | 5.06435720621371 | Tumor | UCEC |
| TCGA-DF-A2KR-01A | 1865 | 0 | 2.7330445347584 | Tumor | UCEC |
| TCGA-EO-A22S-01A | 1872 | 0 | 6.95503045710671 | Tumor | UCEC |
| TCGA-AP-A0LP-01A | 1875 | 0 | 4.34076252358731 | Tumor | UCEC |
| TCGA-B5-A11N-01A | 1876 | 0 | 2.61903093949553 | Tumor | UCEC |
| TCGA-EO-A22R-01A | 1878 | 0 | 5.43266501401001 | Tumor | UCEC |
| TCGA-BS-A0TI-01A | 1882 | 0 | 5.53902860494011 | Tumor | UCEC |
| TCGA-BG-A0MS-01A | 1882 | 0 | 5.09166200354107 | Tumor | UCEC |
| TCGA-BG-A0MH-01A | 1930 | 0 | 5.5964278850525 | Tumor | UCEC |
| TCGA-B5-A0K7-01A | 1933 | 0 | 3.60188957423535 | Tumor | UCEC |
| TCGA-BS-A0V7-01A | 1934 | 0 | 2.47719956272489 | Tumor | UCEC |
| TCGA-BG-A0M7-01A | 1937 | 0 | 4.95691143361336 | Tumor | UCEC |
| TCGA-B5-A0JN-01A | 1939 | 0 | 5.93117701447658 | Tumor | UCEC |
| TCGA-AP-A05A-01A | 1944 | 1 | 5.97902664705912 | Tumor | UCEC |
| TCGA-B5-A0K6-01A | 1953 | 0 | 4.23146851058712 | Tumor | UCEC |
| TCGA-AX-A2IO-01A | 1975 | 0 | 6.08266688001699 | Tumor | UCEC |
| TCGA-A5-A0GJ-01A | 2000 | 0 | 4.52253903823508 | Tumor | UCEC |
| TCGA-A5-A2K3-01A | 2012 | 0 | 6.23405826658388 | Tumor | UCEC |
| TCGA-AP-A0LG-01A | 2015 | 0 | 5.9234148285668 | Tumor | UCEC |
| TCGA-BG-A0M8-01A | 2020 | 0 | 4.33337680995711 | Tumor | UCEC |
| TCGA-BS-A0WQ-01A | 2022 | 0 | 2.34695887506753 | Tumor | UCEC |
| TCGA-FI-A2CX-01A | 2031 | 0 | 4.26364867624908 | Tumor | UCEC |
| TCGA-EO-A22T-01A | 2035 | 0 | 5.9764310734358 | Tumor | UCEC |
| TCGA-AX-A06B-01A | 2058 | 0 | 6.13555674491036 | Tumor | UCEC |
| TCGA-BS-A0TJ-01A | 2068 | 0 | 5.9433040179128 | Tumor | UCEC |
| TCGA-FI-A2CY-01A | 2080 | 0 | 5.9398970375966 | Tumor | UCEC |
| TCGA-A5-A0GX-01A | 2088 | 0 | 3.03751561527615 | Tumor | UCEC |
| TCGA-B5-A0JY-01A | 2107 | 0 | 6.25897067117225 | Tumor | UCEC |
| TCGA-BG-A0M4-01A | 2167 | 0 | 3.38337935157063 | Tumor | UCEC |
| TCGA-AX-A2H5-01A | 2167 | 0 | 3.71565416065206 | Tumor | UCEC |
| TCGA-B5-A0JZ-01A | 2173 | 0 | 5.41128402782272 | Tumor | UCEC |
| TCGA-AX-A05Z-01A | 2175 | 0 | 5.69520495262638 | Tumor | UCEC |
| TCGA-AP-A1DH-01A | 2180 | 0 | 6.14067874992652 | Tumor | UCEC |
| TCGA-B5-A3FC-01A | 2185 | 0 | 4.75043132367429 | Tumor | UCEC |
| TCGA-B5-A0K2-01A | 2209 | 0 | 3.62848916480832 | Tumor | UCEC |
| TCGA-BS-A0UT-01A | 2212 | 0 | 3.41207615299683 | Tumor | UCEC |
| TCGA-BS-A0UV-01A | 2228 | 0 | 6.20403859395575 | Tumor | UCEC |
| TCGA-B5-A11S-01A | 2229 | 0 | 4.12638510648962 | Tumor | UCEC |
| TCGA-FI-A2F4-01A | 2230 | 0 | 5.82999545841808 | Tumor | UCEC |
| TCGA-AX-A1CJ-01A | 2233 | 0 | 4.16494587294827 | Tumor | UCEC |
| TCGA-B5-A11L-01B | 2240 | 1 | 4.3592291335835 | Tumor | UCEC |
| TCGA-A5-A0GQ-01A | 2247 | 0 | 4.13114657079916 | Tumor | UCEC |
| TCGA-A5-A0GH-01A | 2254 | 0 | 6.00497937549194 | Tumor | UCEC |
| TCGA-BG-A0M9-01A | 2270 | 0 | 4.24045798941697 | Tumor | UCEC |
| TCGA-B5-A11I-01A | 2303 | 0 | 5.63855974025795 | Tumor | UCEC |
| TCGA-AX-A1CF-01A | 2337 | 0 | 6.2734570158815 | Tumor | UCEC |
| TCGA-B5-A11R-01A | 2349 | 0 | 4.34638936587231 | Tumor | UCEC |
| TCGA-FI-A2D2-01A | 2352 | 1 | 4.13797096404873 | Tumor | UCEC |
| TCGA-AX-A0J1-01A | 2354 | 0 | 5.93076100715303 | Tumor | UCEC |
| TCGA-BS-A0TG-01A | 2371 | 0 | 3.73330966941942 | Tumor | UCEC |
| TCGA-BS-A0TD-01A | 2379 | 0 | 4.62368804202646 | Tumor | UCEC |
| TCGA-FI-A3PX-01A | 2384 | 0 | 6.20740069662177 | Tumor | UCEC |
| TCGA-E6-A1LZ-01A | 2406 | 0 | 6.16269176216993 | Tumor | UCEC |
| TCGA-AX-A063-01A | 2417 | 0 | 4.67777923877843 | Tumor | UCEC |
| TCGA-AX-A1CK-01A | 2441 | 0 | 4.83281282702433 | Tumor | UCEC |
| TCGA-AX-A05W-01A | 2450 | 0 | 4.56617400026651 | Tumor | UCEC |
| TCGA-AX-A2HA-01A | 2475 | 0 | 5.16914880711734 | Tumor | UCEC |
| TCGA-BS-A0UL-01A | 2481 | 0 | 4.80875122900159 | Tumor | UCEC |
| TCGA-BS-A0UJ-01A | 2506 | 0 | 2.13069677143634 | Tumor | UCEC |
| TCGA-AP-A0LN-01A | 2510 | 0 | 3.23638223789008 | Tumor | UCEC |
| TCGA-A5-A0G9-01A | 2540 | 0 | 4.31987201323042 | Tumor | UCEC |
| TCGA-BS-A0V8-01A | 2544 | 0 | 4.80716567648482 | Tumor | UCEC |
| TCGA-AP-A0LL-01A | 2554 | 0 | 2.70834263710736 | Tumor | UCEC |
| TCGA-AX-A1CI-01A | 2600 | 0 | 3.61129972168179 | Tumor | UCEC |
| TCGA-BS-A0TC-01A | 2602 | 0 | 3.74378096628116 | Tumor | UCEC |
| TCGA-B5-A11O-01A | 2609 | 0 | 5.01238167501652 | Tumor | UCEC |
| TCGA-BS-A0UF-01A | 2611 | 0 | 6.08260296561834 | Tumor | UCEC |
| TCGA-B5-A0JV-01A | 2616 | 0 | 5.2477020846032 | Tumor | UCEC |
| TCGA-AP-A056-01A | 2618 | 0 | 5.49161619043181 | Tumor | UCEC |
| TCGA-B5-A0JS-01A | 2627 | 0 | 3.48301071308847 | Tumor | UCEC |
| TCGA-BS-A0UM-01A | 2644 | 0 | 4.36906125216565 | Tumor | UCEC |
| TCGA-BS-A0VI-01A | 2646 | 0 | 3.36850018925772 | Tumor | UCEC |
| TCGA-A5-A0GI-01A | 2682 | 0 | 5.30869591685467 | Tumor | UCEC |
| TCGA-AX-A05Y-01A | 2684 | 0 | 5.7691551225072 | Tumor | UCEC |
| TCGA-B5-A11H-01A | 2687 | 0 | 5.55025721422961 | Tumor | UCEC |
| TCGA-DF-A2KV-01A | 2717 | 0 | 5.21344120177877 | Tumor | UCEC |
| TCGA-BS-A0V6-01A | 2725 | 0 | 4.6044417112836 | Tumor | UCEC |
| TCGA-AP-A1DK-01A | 2767 | 0 | 4.66637992465554 | Tumor | UCEC |
| TCGA-BS-A0U9-01B | 2825 | 0 | 6.87220301069159 | Tumor | UCEC |
| TCGA-B5-A3FB-01A | 2844 | 0 | 4.61006006278762 | Tumor | UCEC |
| TCGA-AP-A0LF-01A | 2845 | 0 | 5.52800518344573 | Tumor | UCEC |
| TCGA-AP-A0LS-01A | 2904 | 0 | 5.33562737014391 | Tumor | UCEC |
| TCGA-B5-A3FA-01A | 2929 | 0 | 4.77398348484143 | Tumor | UCEC |
| TCGA-BS-A0U5-01A | 2935 | 0 | 4.22109337492597 | Tumor | UCEC |
| TCGA-FI-A2EU-01A | 2941 | 0 | 5.70531074465119 | Tumor | UCEC |
| TCGA-BS-A0U8-01A | 2963 | 0 | 4.50586322183889 | Tumor | UCEC |
| TCGA-AP-A0LI-01A | 2972 | 0 | 6.77390249794899 | Tumor | UCEC |
| TCGA-DF-A2KU-01A | 3067 | 0 | 6.48378479875025 | Tumor | UCEC |
| TCGA-B5-A1MS-01B | 3112 | 1 | 4.94641510743241 | Tumor | UCEC |
| TCGA-A5-A0GE-01A | 3205 | 0 | 5.05864782447603 | Tumor | UCEC |
| TCGA-A5-A0G1-01A | 3251 | 1 | 4.55410608330637 | Tumor | UCEC |
| TCGA-DF-A2KS-01A | 3318 | 0 | 6.13801312306852 | Tumor | UCEC |
| TCGA-DI-A2QY-01A | 3349 | 1 | 5.14555812852011 | Tumor | UCEC |
| TCGA-AP-A0LE-01A | 3357 | 0 | 6.15880829953109 | Tumor | UCEC |
| TCGA-A5-A0GD-01A | 3423 | 1 | 5.65588012335781 | Tumor | UCEC |
| TCGA-B5-A11G-01A | 3448 | 0 | 5.1514817080662 | Tumor | UCEC |
| TCGA-BS-A0UA-01A | 3495 | 0 | 4.02071214502667 | Tumor | UCEC |
| TCGA-AP-A0LD-01A | 3589 | 0 | 6.54951768616707 | Tumor | UCEC |
| TCGA-A5-A2K2-01A | 3595 | 0 | 6.36547451224468 | Tumor | UCEC |
| TCGA-A5-A0GG-01A | 3762 | 0 | 5.87485106796183 | Tumor | UCEC |
| TCGA-AP-A0L9-01A | 3815 | 0 | 6.41685212226345 | Tumor | UCEC |
| TCGA-AP-A051-01A | 3948 | 0 | 5.60212833118569 | Tumor | UCEC |
| TCGA-B5-A3F9-01A | 4155 | 0 | 4.49852247501372 | Tumor | UCEC |
| TCGA-A5-A0G2-01A | 4549 | 0 | 6.11600437056258 | Tumor | UCEC |
| TCGA-B5-A11F-01A | 5651 | 0 | 4.42183310419844 | Tumor | UCEC |
| TCGA-B5-A1MR-01A | 6859 | 0 | 6.02441813080677 | Tumor | UCEC |
